# The risk for the development of hypertensive complications in oocyte donation pregnancy: a systematic review and individual participant data meta-analysis (DONOR IPD)

**DOI:** 10.1093/humupd/dmag006

**Published:** 2026-03-31

**Authors:** Kim van Bentem, Marie-Louise van der Hoorn, Manish Banker, Maria de la Calle, Evangelia Elenis, Dina El Demellawy, Nathan S Fox, Yadava Jeve, Diane Korb, Hélène Letur, Yoav Yinon, Antonio Farina, Francesca Rizzello, Kenny A Rodriguez-Wallberg, Michal Simchen, Serena Simeone, Theoni Tarlatzi, Stefano Raffaele Giannubilo, Saskia Le Cessie, Eileen Lashley

**Affiliations:** Department of Gynecology and Obstetrics, Leiden University Medical Center, Leiden, The Netherlands; Department of Gynecology and Obstetrics, Leiden University Medical Center, Leiden, The Netherlands; Banker IVF & Women’s Hospital, Ahmedabad, India; Department of Obstetrics and Gynecology, La Paz Hospital, Madrid, Spain; Department of Women’s and Children’s Health, Uppsala University Hospital, Uppsala, Sweden; Reproductive Center, Women’s Clinic, Uppsala University Hospital, Uppsala, Sweden; Department of Pediatric Pathology, Children’s Hospital of Eastern Ontario, Faculty of Medicine, University of Ottawa, Ottawa, ON, Canada; Department of Obstetrics, Gynecology, and Reproductive Science, Mount Sinai School of Medicine, New York, NY, USA; Birmingham Women’s and Children’s Hospital, Birmingham, United Kingdom; Department of Obstetrics and Gynecology, Robert Debré Hospital, Assistance Publique-Hôpitaux de Paris, Paris, France; Department of Obstetrics and Gynecology, Reproductive Medicine and Fertility Preservation, Foch Hospital, Suresnes, France; Department of Reproductive Medicine and Fertility Preservation, Pau Pyrénées Polyclinic, Pau, France; Department of Obstetrics and Gynecology, Sheba Medical Center, Tel-Hashomer, Faculty of Medical and Health Sciences, Tel Aviv University, Tel Aviv, Israel; Obstetric Unit, IRCCS Azienda Ospedaliero-Universitaria di Bologna, Bologna, Italy; Department of Medical and Surgical Sciences (DIMEC), Alma Mater Studiorum-University of Bologna, Bologna, Italy; Assisted Reproductive Technology Centre, Careggi University Hospital, Florence, Italy; Department of Reproductive Medicine, Division of Gynecology and Reproduction, Karolinska University Hospital & Karolinska Institutet, Stockholm, Sweden; Department of Obstetrics and Gynecology, Sheba Medical Center, Tel-Hashomer, Tel Aviv University, Tel Aviv, Israel; Department of High Risk Pregnancy, Careggi University Hospital, Florence, Italy; Fertility Clinic, Department of Obstetrics and Gynecology, Erasme Hospital, Université Libre de Bruxelles, Brussels, Belgium; Department of Clinical Sciences, Università Politecnica delle Marche, Ancona, Italy; Department of Clinical Epidemiology, Leiden University Medical Center, Leiden, The Netherlands; Department of Gynecology and Obstetrics, Erasmus Medical Center, Rotterdam, The Netherlands

**Keywords:** individual participant data meta-analysis, oocyte donation, pregnancy, hypertensive pregnancy complications, pregnancy induced hypertension, preeclampsia

## Abstract

**BACKGROUND:**

Oocyte donation (OD) is an established ART involving an oocyte donor and recipient with a rising number of treatments. Previous meta-analyses highlight increased risks of hypertensive complications compared to naturally conceived (NC) and IVF/ICSI pregnancies, including pregnancy-induced hypertension (PIH) and preeclampsia (PE), but limitations exist due to study quality and heterogeneity.

**OBJECTIVE AND RATIONALE:**

The DONOR (DONation of Oocytes in Reproduction) individual participant data (IPD) meta-analysis aims to generate clinically relevant and robust evidence regarding the development of hypertensive complications in OD pregnancies compared to autologous pregnancies. IPD meta-analyses offer an advantage over current meta-analyses, as bias is reduced by using IPD of original studies, allowing reliability checks, correction for confounders, and examining causes of heterogeneity by subgroup analyses. Furthermore, using IPD increases statistical power and generalizability of results.

**SEARCH METHODS:**

A literature search was conducted using PubMed, EMBASE, and Cochrane up to March 2024, with a last update performed in February 2025. We included observational studies that compared a cohort of women pregnant after OD beyond 20 weeks of gestation with an autologous pregnancy cohort (NC or IVF/ICSI), and reported on hypertensive pregnancy complications. Risk of bias was assessed using the ROBINS-I tool. Authors of eligible articles were invited to share IPD. The DONOR IPD meta-analyses were executed using both a one- and two-stage approach, adjusted for maternal age, parity, and multiple gestation. Furthermore, sensitivity, meta-regression and subgroup analysis were performed.

**OUTCOMES:**

IPD was requested for 48 cohorts, and provided from 16 cohorts with data of 2747 OD, 4699 IVF/ICSI, and 33 323 NC pregnancies. The one- and two-stage approach comparing OD to autologous pregnancies showed adjusted ORs of respectively 2.62 (95% CI 2.22–3.10) and 2.85 (95% CI 2.30–3.54; *I*^2^ 44%; moderate certainty) for hypertensive complications in total, 2.15 (95% CI 1.73–2.68) and 1.49 (95% CI 0.80–2.80; *I*^2^ 74%; low certainty) for PIH, and 2.28 (95% CI 1.88–2.78) and 2.39 (95% CI 1.94–2.94; *I*^2^ 0%; high certainty) for PE. When the autologous group was split into NC and IVF/ICSI pregnancies, higher risks for hypertensive complications, including PIH and PE, persisted in the OD group. The results of the IPD meta-analyses for HELLP syndrome show a higher risk in OD pregnancy, though with a broad 95% CI. Sensitivity meta-analyses for risk of bias showed comparable results. Subgroup analyses indicated increased risks for hypertensive complications in OD pregnancy, regardless of maternal age, BMI, multiple pregnancy, parity, ethnicity, medical history, and number of transferred embryos. A potential lower risk for hypertensive complications was found when acetylsalicylic acid or heparin is used during OD pregnancy compared to both autologous and NC pregnancy.

**WIDER IMPLICATIONS:**

The DONOR IPD meta-analysis provided a unique opportunity to assess the risk for hypertensive complications in OD compared to autologous pregnancy. The results must increase alertness of health care professionals who are involved in OD health care towards the risk profile of these pregnancies, as the DONOR IPD meta-analysis results in the best evidence-based statement for international guidelines in obstetrics to date. Possibly, preventive treatment with low-dose acetylsalicylic acid is successful in lowering the risk for hypertensive complications, though more evidence is needed to confirm this effect, alongside the underlying pathological mechanism.

**REGISTRATION NUMBER:**

CRD42021267908.

## Graphical Abstract

**Figure:**
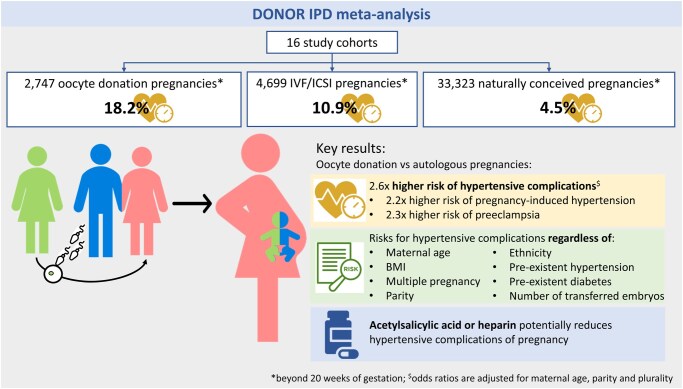

## Introduction

Oocyte donation (OD) is an ART that resembles the method of IVF with the difference in that the oocyte is obtained from a donor. OD has been successfully executed for several decades now (Lutjen *et al.*, 1984), and is anticipated to rise in the forthcoming years. Due to the extension of indications (Bustillo *et al.*, 1984; Sauer *et al.*, 1991; Kavic and Sauer, 2001; Klein and Sauer, 2002; van der Hoorn *et al.*, 2010), such as reception of the oocyte of the partner (ROPA) (Marina *et al.*, 2010), and more importantly a growing population postponing pregnancy, the numbers are still rising (Wyns *et al.*, 2020).

Intriguingly, while OD is increasing in popularity due to high success rates, OD pregnancies are accompanied by a higher incidence of obstetric complications (van der Hoorn *et al.*, 2010). Up to this date, various meta-analyses have indicated an increased risk of hypertensive complications in pregnancy, including pregnancy-induced hypertension (PIH) and preeclampsia (PE), in OD pregnancies compared to autologous IVF or ICSI, and naturally conceived (NC) pregnancies (Pecks *et al.*, 2011; Blazquez *et al.*, 2016; Masoudian *et al.*, 2016; Savasi *et al.*, 2016; Jeve *et al.*, 2016a; Storgaard *et al.*, 2017; Moreno and Miguel, 2019; Keukens *et al.*, 2022). Meta-analyses are, however, limited by the quality and heterogeneity of included studies. To investigate the causal relation between OD pregnancy and the development of hypertensive complications, correction for important confounding factors is of great importance, including advanced maternal age, primiparity, obesity, ethnicity, and multiple gestation (Dekker, 1999; Jacobsson *et al.*, 2004; Duckitt and Harrington, 2005; Gaillard *et al.*, 2011; Mol *et al.*, 2016; van Bentem *et al.*, 2019). In most individual studies included in these meta-analyses, however, a considerable amount of bias remains that could influence this association.

Individual participant data (IPD) meta-analyses offer an advantage over current meta-analyses, as bias may be reduced by using the IPD of the original studies, which enables checking the reliability of the data and examine causes for heterogeneity by subgroup analyses. Furthermore, using IPD increases statistical power, enables adjustment for multiple confounding factors, and improves generalizability of results (Stewart and Parmar, 1993; Riley *et al.*, 2010). Therefore, the primary objective of the current DONOR (DONation of Oocytes in Reproduction) IPD meta-analysis is to generate clinically relevant and robust evidence regarding the development of hypertensive complications in OD pregnancies compared to autologous pregnancies (NC or non-donor IVF/ICSI).

## Methods

### PROSPERO registration and systematic search

The DONOR IPD meta-analysis is conducted according to the registered and published protocol (PROSPERO CRD42021267908) (van Bentem *et al.*, 2022), and the results are stated according to the Preferred Reporting Items for Systematic Review and Meta-Analyses of Individual Participant Data (PRISMA-IPD) statement (Stewart *et al.*, 2015). A final search update in PubMed was performed in March 2024 based on the existing search strategy (van Bentem *et al.*, 2022), using medical subject heading terms for OD, embryo disposition, pregnancy, PIH, IVF, and ICSI. In addition, other electronic databases were searched, including EMBASE and Cochrane. The detailed search strategies are presented in Supplementary Data File S1.

### Eligibility criteria

Inclusion and exclusion criteria for studies were determined before the first literature search in September 2020. We included published and unpublished (e.g. conference abstracts) observational studies that compared a cohort of women pregnant after OD beyond 20 weeks of gestation with an autologous pregnancy cohort (e.g. IVF/ICSI, NC). Studies were excluded if they included only patients with Turner syndrome, used a non-autologous comparison group (e.g. double gamete donation) or were non-comparative at all, or when the primary outcome was not reported. Selection was not restricted to the English language or year of publication.

### Study selection and data collection

Study selection on title and abstract was done by two researchers independently (K.B. and E.L.). Subsequently, the full texts of the selected articles were assessed for compliance with the inclusion criteria. Any disagreement was resolved by discussion and consensus. Furthermore, reference lists of relevant articles were scanned to identify additional studies. When more publications originated from the same center, population, or cohort, duplicates were identified by cross-referencing and contacting the authors for clarification. The corresponding authors of all eligible studies were invited to collaborate in the DONOR IPD meta-analysis and share their IPD. When no response was received, at least three more reminders were sent, and fellow authors were contacted as well. All authors sharing the IPD were given the possibility to sign a data transfer agreement (van Bentem *et al.*, 2022), and were asked to provide clarifications when information in the publications or datasets were unclear or inconsistent. All IPD was merged into a master dataset for analysis, using the data management system Castor EDC (https://www.castoredc.com/). Before analysis, all IPD were checked and cleaned, excluding cases with pregnancy loss before 20 weeks of gestation. If IPD were unavailable from selected studies (e.g. because of non-responsiveness or legal constraints), they were included in the aggregate data meta-analysis when possible. Lastly, we organized an online meeting in March 2023 to strengthen current and future collaboration and clarify plans for all authors, regardless of whether they could share the IPD or not.

### Outcomes

The primary outcome, hypertensive complication of pregnancy, was defined according to the International Society for the Study of Hypertension in Pregnancy (ISSHP) classification (Tranquilli *et al.*, 2014). Pregnancy-induced hypertension (PIH) was defined as *de novo* development of high blood pressure detected after 20 weeks of gestation, with systolic blood pressure ≥140 mmHg and/or diastolic blood pressure ≥90 mmHg. Preeclampsia (PE) was defined as hypertension and the coexistence of one or more of the following: (1) proteinuria (>300 mg/l on dipstick testing, spot urine protein/creatinine >30 mg/mmol, or a urine protein excretion of >300 mg in 24 h); or (2) other maternal organ dysfunction (e.g. renal insufficiency, liver involvement, neurological complications, hematological complications); or (3) uteroplacental dysfunction manifesting in fetal growth restriction (Tranquilli *et al.*, 2014; Mol *et al.*, 2016). As this definition was renewed in 2014, most of the included studies maintained the definition of PE as hypertension with the coexistence of proteinuria. As secondary outcomes, the severity and time to development of PE are taken into account. Severe PE was defined by systolic blood pressure ≥160 mmHg or diastolic ≥110 mmHg, or in the presence of HELLP (hemolysis, elevated liver enzymes, and low platelets) syndrome (Tranquilli *et al.*, 2013b). Early-onset PE was considered when occurring before 34 weeks of gestation (Tranquilli *et al.*, 2013b).

### Risk of bias assessment

The risk of bias was assessed by two reviewers independently (K.B. and E.L.) according to the ROBINS-I tool (Risk Of Bias In Non-randomized Studies–of Interventions) (Sterne *et al.*, 2016). To create risk of bias plots, the risk of bias visualization (robvis) tool was used (McGuinness and Higgins, 2021). Furthermore, a written description of the various domains of bias was made according to a validation checklist developed by Scholten *et al.* (2018). Disagreement was resolved by discussion and consensus.

### Statistical analysis

IPD were extracted from Castor EDC and prepared in Microsoft Excel. Statistical computing was performed in R version 4.2.1 (accessed through Leiden University Medical Center, Leiden, the Netherlands) using the *lme4*, *meta*, *mice, metafor, readxl, dplyr,* table1*, knitr, stats* and *forestplot* R packages. Descriptive statistics were computed to compare baseline characteristics between the groups. NC and IVF/ICSI pregnancies were analyzed both as one autologous pregnancy group and as two separate control groups. For all tests, a two-sided *P* < 0.05 or 95% confidence interval not including the null value was considered statistically significant.

Both a two-stage approach, where effect estimates were calculated for each study separately and then pooled in a random effects meta-analysis, and a one-stage approach, where IPD from all studies were analyzed simultaneously using a logistic mixed-effect model, were performed (Stewart *et al.*, 2012; Debray *et al.*, 2015; Burke *et al.*, 2017). The models were adjusted for confounding factors, including the recipient characteristics, maternal age, parity, and plurality, which is crucial for estimating the causal relation between OD pregnancy and the development of hypertensive complications (van Bentem *et al.*, 2019).

Multiple imputation and the associated sensitivity analysis for best and worst case scenarios, as described in the DONOR IPD protocol (van Bentem *et al.*, 2022) were not needed due to low percentages of missing covariate data; however, the cohort of Banker *et al.* (2016) did not report on parity. Therefore, it was decided to assign nulliparity to all inclusions of Banker *et al.* (2016) instead of imputing as the majority of total inclusions is nulliparous (ranging from 41.1% (Levron *et al.*, 2014) to 96.2% (Elenis *et al.*, 2015)), and multiple imputation would not be accurate enough when the covariate is missing for all inclusions from a single cohort. Heterogeneity between studies was assessed using the between-study effect variation tau^2^ and the percentage of total variation due to heterogeneity *I*^2^ statistic, and random effects meta-analysis models were used to account for heterogeneity. The outcomes were reported as odds ratios (OR) and adjusted odds ratios (aOR) with the corresponding 95% CI.

Pre-planned analyses to study the effects in specific subgroups were performed. This was done by adding an interaction term between mode of conception and subgroup, using the one-stage IPD meta-analysis approach. Statistical significance was found when the CI did not overlap with the original one-stage IPD meta-analyses results, and the interaction term showed a *P*-value <0.05. The analyses included subgroups of multiple pregnancy, maternal age, ethnicity, parity, BMI, indication for OD, donor-recipient familiar relationship, number of transferred embryos, use of salicylic acid or heparin during pregnancy, and higher risk of PE based on medical history. However, the possibility of performing these subgroup analyses depended on the availability of IPD.

A sensitivity analysis was performed, in which IPD data and aggregate data from those studies where IPD could not be obtained were combined in a two-stage meta-analysis, exploring IPD availability bias. Furthermore, the impact of methodological quality was assessed by excluding studies evaluated with a serious or critical risk of bias, using both the one- and two-stage approach. Because the inclusion of studies spanned a long period in which the definition for PE changed over time, meta-regression analyses were performed to investigate the effect of publication year on the relation between OD pregnancies and the different outcomes and the study’s effect sizes.

To evaluate the potential for publication bias, funnel plots were constructed by plotting the standard error of the effect sizes against the log ORs from individual studies from both the two-stage IPD meta-analyses and the aggregate data meta-analyses. Additionally, Egger’s test was applied to quantitatively assess funnel plot asymmetry. A *P*-value <0.05 was considered indicative of potential publication bias.

## Results

### Study selection and IPD retrieval

**Figure 1.**
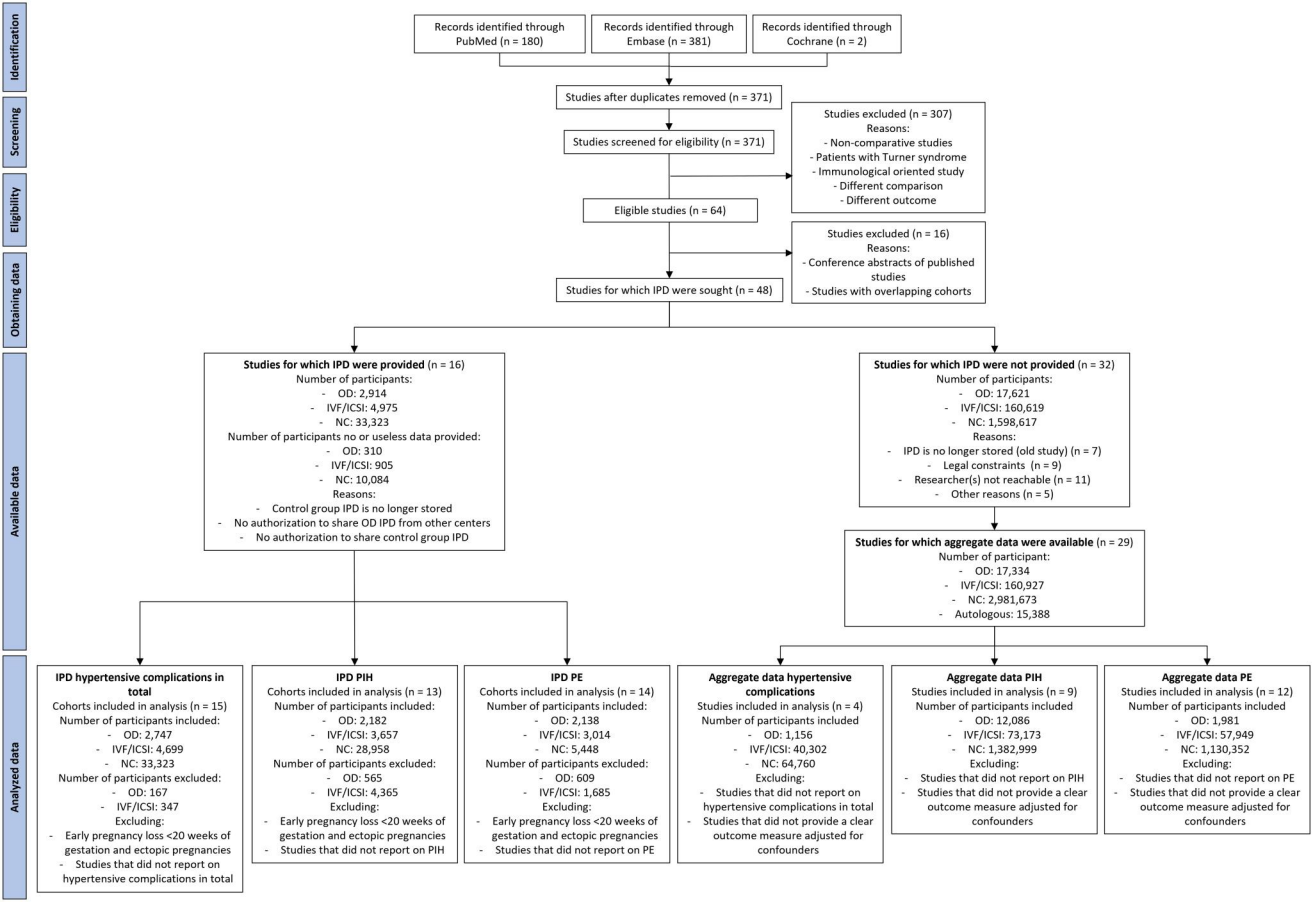
**PRISMA IPD flow diagram of the study selection and data retrieval and analysis strategy**.

The detailed study selection process is presented in the PRISMA flow chart (Fig. 1). The systematic search yielded a total of 563 papers, resulting in 371 studies after removing duplicates. Titles and abstracts were screened, and full texts of relevant articles were assessed for eligibility. Sixty-four studies met the inclusion criteria, and IPD was requested for 48 cohorts as the eligible studies also included six conference abstracts of published studies, and 10 studies with overlapping cohorts. IPD of 32 studies were not available, due to either insufficient contact information (Wolff *et al.*, 1997), no response despite a minimal of three attempts (Henne *et al.*, 2007; Krieg *et al.*, 2008; Shrim *et al.*, 2010; Jackson *et al.*, 2015; Yamada *et al.*, 2015; Sites *et al.*, 2017; Hayashi *et al.*, 2019; Rudenko *et al.*, 2020; Gekka *et al.*, 2021; Zeman *et al.*, 2022), data loss (Salha *et al.*, 1999; Wiggins and Main, 2005; Klatsky *et al.*, 2010; Stoop *et al.*, 2012; Van Dorp *et al.*, 2014; Dior *et al.*, 2018; Vikas *et al.*, 2022), legal constraints (Söderström-Anttila *et al.*, 1998; Le Ray *et al.*, 2012; Malchau *et al.*, 2013; Nejdet *et al.*, 2016; Guilbaud *et al.*, 2017; Kennedy *et al.*, 2019; Luke *et al.*, 2020; Meyer *et al.*, 2020; Koren *et al.*, 2021), or other reasons (Keegan *et al.*, 2007; Rizzo *et al.*, 2016; Woo *et al.*, 2017; Barnard *et al.*, 2019; Modest *et al.*, 2019). Eventually, IPD were provided for 16 fully published studies (Simchen *et al.*, 2009; Tranquilli *et al.*, 2013a; Levron *et al.*, 2014; Sekhon *et al.*, 2014; Elenis *et al.*, 2015; Banker *et al.*, 2016; Letur *et al.*, 2016; Jeve *et al.*, 2016b; Tarlatzi *et al.*, 2017; Fassio *et al.*, 2019; Rodriguez-Wallberg *et al.*, 2019; Serena *et al.*, 2019; Boria *et al.*, 2020; Esteves *et al.*, 2020; Korb *et al.*, 2020; Rizzello *et al.*, 2020), resulting in IPD from 16 cohorts in total with data of 2747 OD, 4699 autologous IVF/ICSI, and 33 323 NC pregnancies. Six cohorts provided IPD of an OD and NC group (Sekhon *et al.*, 2014; Elenis *et al.*, 2015; Jeve *et al.*, 2016b; Boria *et al.*, 2020; Korb *et al.*, 2020; Rizzello *et al.*, 2020), and 11 cohorts provided IPD of an OD and IVF/ICSI group (Sekhon *et al.*, 2014; Elenis *et al.*, 2015; Banker *et al.*, 2016; Letur *et al.*, 2016; Jeve *et al.*, 2016b; Tarlatzi *et al.*, 2017; Serena *et al.*, 2019; Boria *et al.*, 2020; Esteves *et al.*, 2020; Korb *et al.*, 2020; Rizzello *et al.*, 2020). Several researchers were only able to share data of the OD group and not of the control groups. Therefore, the two-stage meta-analysis had to exclude the cohorts of Levron *et al.* (2014), Rodriguez-Wallberg *et al.* (2019), Simchen *et al.* (2009), and Tranquilli *et al.* (2013a). Furthermore, Boria *et al.* (2020) shared too small a number of control IPD, preventing a two-stage approach. As several months passed between the final search update and the execution of the IPD meta-analyses, we performed one final search update in February 2025, showing two more eligible studies of which IPD were not requested (Horta *et al.*, 2024; Donno *et al.*, 2025).

### Study characteristics

The characteristics of all 48 eligible studies are reported in Supplementary Table S1. All studies were retrospective cohort studies, apart from four prospective cohort studies (Banker *et al.*, 2016; Rizzo *et al.*, 2016; Rodriguez-Wallberg *et al.*, 2019; Korb *et al.*, 2020). All report hypertensive complications of pregnancy; six reported only PIH (Wolff *et al.*, 1997; Keegan *et al.*, 2007; Simchen *et al.*, 2009; Shrim *et al.*, 2010; Yamada *et al.*, 2015; Koren *et al.*, 2021), six only PE (Krieg *et al.*, 2008; Le Ray *et al.*, 2012; Nejdet *et al.*, 2016; Rizzo *et al.*, 2016; Modest *et al.*, 2019; Zeman *et al.*, 2022), 28 reported both PIH and PE (Söderström-Anttila *et al.*, 1998; Salha *et al.*, 1999; Wiggins and Main, 2005; Klatsky *et al.*, 2010; Stoop *et al.*, 2012; Malchau *et al.*, 2013; Tranquilli *et al.*, 2013a; Levron *et al.*, 2014; Sekhon *et al.*, 2014; Van Dorp *et al.*, 2014; Jackson *et al.*, 2015; Banker *et al.*, 2016; Letur *et al.*, 2016; Jeve *et al.*, 2016b; Sites *et al.*, 2017; Tarlatzi *et al.*, 2017; Woo *et al.*, 2017; Dior *et al.*, 2018; Barnard *et al.*, 2019; Kennedy *et al.*, 2019; Serena *et al.*, 2019; Boria *et al.*, 2020; Luke *et al.*, 2020; Meyer *et al.*, 2020; Rizzello *et al.*, 2020; Rudenko *et al.*, 2020; Gekka *et al.*, 2021; Vikas *et al.*, 2022), seven reported PIH and/or PE plus HELLP syndrome (Henne *et al.*, 2007; Elenis *et al.*, 2015; Guilbaud *et al.*, 2017; Fassio *et al.*, 2019; Rodriguez-Wallberg *et al.*, 2019; Esteves *et al.*, 2020; Korb *et al.*, 2020), and one reported hypertensive complications unspecified (Hayashi *et al.*, 2019). The number of OD pregnancies varied from 13 to 11 309 per study; in all studies an autologous control group was included. The studies originate from countries of all continents, except African countries. Publication dates range between 1997 and 2022, and all are published in English. The characteristics of the 16 studies that were able to share IPD are depicted in Table 1. Publication dates from these sixteen studies ranged between 2009 and 2020.

**Table 1.** Study characteristics of studies that shared their individual participant data.

Study	Journal	Country	De-sign	Study period	Inclusion criteria	Exclusion criteria	Control group	Participants (n)	Mean maternal age (years)	Outcome	Definition of outcome
Banker (2016)	*J Hum Reprod Sci*	India	PC	2014	All women who conceived after embryo transfer following IVF/ICSI with or without OD	Not stated	Fresh embryo transfer using self-oocytes and thaw embryo transfer using vitrified-warmed embryos	691 fresh IVF; 611 fresh OD; 810 thawed IVF	Fresh IVF: 30.68 ± 3.65; fresh OD: 36.65 ± 5.18; thawed IVF: 32.54 ± 5.04	PIH, PE	Not stated
Boria (2020)	*J Matern Fetal Neonatal Med*	Spain	RC	2012–2016	Twin OD pregnancies delivered after 24 weeks of gestation	Not stated	Twin pregnancies after autologous IVF with a due date within two months before or after that of the case	50 OD 50 IVF	OD: 40 (38.7–43.2) IVF: 36 (34–40)	PIH, PE	PIH = systolic >140 or diastolic blood pressure >90 after 20 weeks; PE = PIH with proteinuria >300 mg/24h
Elenis (2015)	*BMC Pregnancy Childbirth*	Sweden	RC	2005–2008	Singleton OD pregnancies	Women who did not speak or read Swedish	1. Age-matched nulliparae with singleton NC pregnancies and no history of subfertility 2. Heterosexual women with singleton autologous IVF pregnancies	76 OD 150 NC 63 IVF	OD: 35.0 (25–43) NC: 34.0 (19–36) IVF: 33.0 (25–39)	PIH, PE, HELLP, eclampsia	Definitions according to the ICD-10
Esteves (2020)	*Placenta*	Canada	RC	2011–2018	All clinical cases of OD patients with a placental pathology report and complete antenatal history	OD pregnancies without placental pathology report and no relevant clinical antenatal history	ART cases who did not achieve pregnancy through OD	85 OD 270 ART	OD: 41.53 ± 6.26 (28–55) ART: 35.30 ± 4.47 (23–50)	Hypertensive complications	Not stated
Fassio (2019)	*J Clin Med*	Italy	RC	2008–2019	Singleton OD pregnancies >24 weeks, with complete data at delivery	Abortions and therapeutic interruptions of pregnancy	NC singleton pregnancy occurring in a woman without pre-existing, systemic and localized diseases	296 OD 1407 NC	OD: 44 (31–56) NC: 31 (15–49)	PIH, PE, HELLP	PIH = hypertension during pregnancy in previously normotensive patients
Jeve (2016)	*Int J Gynaecol Obstet*	UK	RC	2007–2014	OD pregnancy and delivery of a live neonate after 24 weeks at a teaching hospital in Leicester in the period	Pregnancies after preimplantation genetic diagnosis and surgical sperm retrieval or use of donor sperm	Age-matched IVF and NC pregnancies	45 OD 45 IVF 45 NC	OD: 40.23 ± 5.64 IVF: 39.23 ± 1.71 NC: 39.69 ± 0.71	PIH, PE	PIH = blood pressure >140/90 mmHg after 20 weeks; PE= PIH with proteinuria ≥0.3 g/day
Korb (2020)	*Hum Reprod*	France	PC	2014–2015	Twin pregnancies ≥22 weeks of gestation in the JUMODA cohort, conceived after medically assisted reproduction	Unknown mode of conception	NC twin pregnancies ≥22 weeks of gestation in the JUMODA cohort	329 OD 5890 NC 854 non-IVF 1307 IVF 368 ICSI	OD: 40.5 ± 4.9 NC: 30.6 ± 5.1 Non-IVF: 31.7 ± 4.5 IVF: 33.6 ± 4.7 ICSI: 32.6 ± 4.4	SAMM, PE, HELLP, Eclampsia	PE = hypertension ≥140/90 and proteinuria ≥0.3 g/24 h
Letur (2016)	*Fertil Steril*	France	RC	2005–2012	Singleton OD pregnancies from seven ART centers	Multiple pregnancies, OD treatment abroad	Singleton autologous IVF/ICSI pregnancies at the same center	217 OD 363 controls	OD: 34.4 ± 9.4 Controls: 34.2 ± 4.5	PIH, PE, eclampsia	PIH = blood pressure ≥140/90 mmHg after 20 weeks; PE = repeated ≥140/90 mmHg, with proteinuria ≥0.3 g/day; Eclampsia = generalized seizures in a context of severe preeclampsia (repeated ≥160/110 mmHg, proteinuria ≥3 g/day).
Levron (2014)	*Am J Obstet Gynecol*	Israel	RC	2005–2011	Singleton OD pregnancies with prenatal and delivery care at a tertiary medical center	Congenital or chromosomal abnormalities, multiple pregnancies	Women >38 years with autologous IVF pregnancy in same time period, delivery at the same center	139 OD 126 IVF	OD: 45 (23–57) IVF: 41 (38–46)	PIH, PE, hypertensive complications	PIH = blood pressure ≥140/90 mmHg after 20 weeks; PE = PIH with proteinuria (≥300 mg/24 h or 2+ dipstick).
Rizzello (2020)	*RBMO*	Italy	RC	2009–2017	Singleton OD pregnancies of women giving birth at the University Hospital of Careggi, Florence	Multiple pregnancies	Singleton IVF/ICSI and NC pregnancies	276 OD 925 IVF/ICSI 24 650 NC	OD: 43.5 ± 4.5 IVF/ICSI: 35.6 ± 4.3 NC: 32.8 ± 5.4	Hypertensive complications	Included chronic hypertension and *de novo* hypertension, either PIH or PE.
Rodriguez-Wallberg (2019)	*Gynecol Endocrinol*	Sweden	PC	2007–2014	Women who achieved singleton pregnancies and live births using donor oocytes after IVF with or without ICSI	Women aged 40 yrs or older at time of IVF treatment or having medical conditions	Women undergoing IVF/ICSI with autologous oocytes, matched (1:2) by age, IVF or ICSI, and year of embryo transfer	259 OD 515 IVF/ICSI	OD: 35.0 ± 3.79 IVF/ICSI: 34.8 ± 3.61	PIH, PE, HELLP	Definitions according to the ICD-10
Sekhon (2014)	*Fertil Steril*	United States	RC	2005–2013	OD twin pregnancies	Women aged >50 years, monochorionic-monoamniotic placentation	Age-matched autologous IVF twin pregnancies	56 OD 56 IVF	OD: 43.0 ± 6.0 IVF: 41.9 ± 1.7	PIH, PE	PIH = systolic of ≥140 mmHg or diastolic blood pressure of ≥90 mmHg after 20 weeks; PE = PIH plus proteinuria (≥300 mg/24 h)
Serena (2019)	*Minerva Ginecol*	Italy	RC	2011–2017	Singleton OD pregnancies referred at a high-risk pregnancy unit, gestational age ≥24 weeks at delivery	Sperm or double donation, multiple pregnancies, abortion <24 wks	Singleton IVF and NC pregnancies that gave birth at the same time	290 OD 290 IVF 870 NC	OD: 43.4 ± 2.9 IVF: 37.7 ± 2.4 NC: 33.6 ± 5.5	PIH, PE	PIH = systolic ≥140 mmHg and diastolic blood pressure ≥90 mmHg after 20 week; PE = PIH with proteinuria ≥3 g/24h
Simchen (2009)	*Hum Reprod*	Israel	RC	1999–2008	Twin OD pregnancies of women of 40 years or older	Not stated	Singleton OD pregnancies of similar women, and all women carrying twins in 2007	83 OD singleton 42 OD twin 417 control twin	OD singleton: 49.3 ± 4.7; OD twin: 49.2 ± 4.3 Controls: 31.6 ± 6.5	PIH	PIH = systolic ≥140 mmHg or diastolic blood pressure ≥90 mmHg after 20 weeks
Tarlatzi (2017)	*RBMO*	Belgium	RC	1991–2013	Singleton OD pregnancies, delivery after more than 22 weeks of gestation	Turner syndrome, multiple pregnancies, testicular sperm extraction, preimplantation genetic diagnosis, cryopreserved embryos	Singleton IVF/ICSI pregnancies, delivery after more than 22 weeks of gestation at the same hospital during the same period	144 OD 144 IVF/ICSI	Both groups: 35.64 ± 4.54 (22–43)	PIH, PE	PIH = blood pressure ≥140/90 mmHg after 20 weeks of gestation; PE = PIH with proteinuria ≥0.3 g/day after 20 weeks of gestation
Tranquilli (2013a)	*J Matern Fetal Neonatal Med*	Italy	RC	Not stated	ICSI pregnancies using heterologous oocytes	Not stated	Homologous ICSI and NC pregnancies in women >40 years	26 OD 52 ICSI 52 NC	OD: 42.7 (28–52) ICSI: 37.5 (29–47) NC: 41.5 (40–45)	PIH, PE	PIH = systolic ≥140 mmHg and/or diastolic blood pressure ≥90 mmHg after 20 weeks; PE = PIH with proteinuria ≥300 mg/day or urine protein/creatinine ratio ≥30 mg/mmol

AMA = advanced maternal age; HELLP = hemolysis, elevated liver enzymes, and low platelets; n = number; NC = naturally conceived; OD = oocyte donation; PC = prospective cohort study; PE = preeclampsia; PIH = pregnancy induced hypertension; RC = retrospective cohort study; SAMM = severe acute maternal morbidity, defined as any of the following: maternal death, severe postpartum hemorrhage i.e. transfusion ≥4 units of packed red blood cells, uterine artery embolization, vascular ligation, compressive uterine suture, emergency peripartum hysterectomy, postpartum hemorrhage requiring second line therapy, eclampsia, HELLP syndrome with admission to an intensive care unit, pre-eclampsia (defined as hypertension ≥140/90 and proteinuria ≥0.3 g/24 h) only if it induced preterm delivery for a main maternal indication before 32 gestational weeks; yrs = years.

### Risk of bias assessment

**Figure 2.**
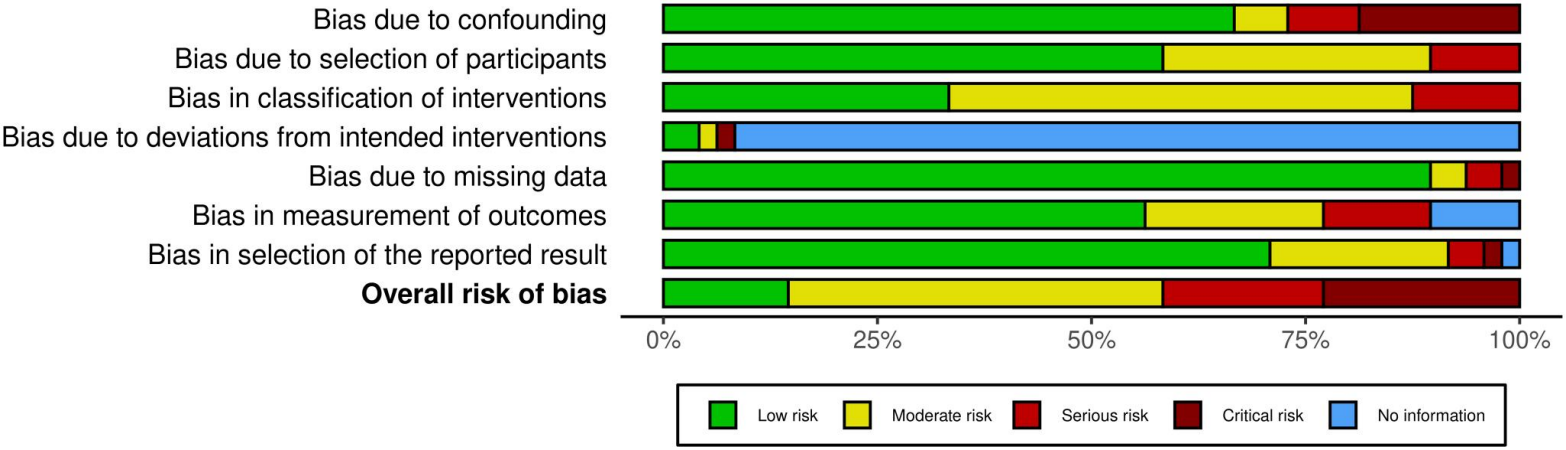
**Summary of the risk of bias from all eligible studies**.

The methodological quality assessment for each eligible study according to the ROBINS-I tool is detailed in Supplementary Data File S2, and Fig. 2 shows the summary for risk of bias. Of the 48 included studies, seven were graded with low risk of bias (Wiggins and Main, 2005; Stoop *et al.*, 2012; Sekhon *et al.*, 2014; Letur *et al.*, 2016; Sites *et al.*, 2017; Dior *et al.*, 2018; Esteves *et al.*, 2020), 21 were graded with moderate risk of bias (Söderström-Anttila *et al.*, 1998; Klatsky *et al.*, 2010; Le Ray *et al.*, 2012; Malchau *et al.*, 2013; Levron *et al.*, 2014; Van Dorp *et al.*, 2014; Elenis *et al.*, 2015; Nejdet *et al.*, 2016; Rizzo *et al.*, 2016; Jeve *et al.*, 2016b; Guilbaud *et al.*, 2017; Kennedy *et al.*, 2019; Modest *et al.*, 2019; Rodriguez-Wallberg *et al.*, 2019; Serena *et al.*, 2019; Boria *et al.*, 2020; Korb *et al.*, 2020; Luke *et al.*, 2020; Meyer *et al.*, 2020; Rizzello *et al.*, 2020; Gekka *et al.*, 2021), 9 were graded with serious risk of bias (Wolff *et al.*, 1997; Salha *et al.*, 1999; Henne *et al.*, 2007; Shrim *et al.*, 2010; Tarlatzi *et al.*, 2017; Woo *et al.*, 2017; Barnard *et al.*, 2019; Koren *et al.*, 2021; Vikas *et al.*, 2022), and 11 were graded with critical risk of bias (Keegan *et al.*, 2007; Krieg *et al.*, 2008; Simchen *et al.*, 2009; Tranquilli *et al.*, 2013a; Jackson *et al.*, 2015; Yamada *et al.*, 2015; Banker *et al.*, 2016; Fassio *et al.*, 2019; Hayashi *et al.*, 2019; Rudenko *et al.*, 2020; Zeman *et al.*, 2022).

### Hypertensive complications in OD compared to autologous pregnancies

The baseline characteristics of the IPD are shown in Table 2. The IPD of 15 cohorts were used for the meta-analyses to compare the incidence of hypertensive complications in total between OD and autologous pregnancies. IPD of Fassio *et al.* (2019) could not be used, as they only provided data on PE, but it remained unclear whether the cases without PE were also without PIH. To compare the incidence of PIH between OD and autologous pregnancies, the IPD of 14 cohorts was used. Data from the cohorts of Jeve *et al.* (2016b), and Fassio *et al.* (2019) were excluded, as they did not provide data on PIH. To assess the incidence of PE between OD and autologous pregnancies, the IPD of fourteen cohorts were used for the meta-analyses. IPD from the cohorts of Jeve *et al.* (2016b), and Rizzello *et al.* (2020) were excluded, as information on PE was lacking. The IPD of 11 cohorts were used for the meta-analyses to compare the incidence of HELLP syndrome between OD and autologous pregnancies. Jeve *et al.* (2016b), Korb *et al.* (2020), Rizzello *et al.* (2020), Tarlatzi *et al.* (2017), and Simchen *et al.* (2009) did not provide data on HELLP syndrome, and thus were excluded in the analyses.

**Table 2.** Baseline characteristics of individual participant data.

	OD	IVF/ICSI	NC
	(*n* = 2747)	(*n* = 5193)	(*n* = 33 323)
	Mean (SD)	Mean (SD)	Mean (SD)

Maternal age (years)	39.8 (6.12)	34.4 (4.82)	32.4 (5.37)
BMI (kg/m^2^)	24.1 (4.20)	23.5(4.48)	22.9 (4.27)
Missing (% (n))	20.0 (550)	14.2 (669)	4.5 (1499)
Gestational age (days)	260 (22.8)	258 (23.9)	270 (19.6)
Missing (% (n))	2.3 (62)	0.4 (17)	0.2 (52)
Birth weight singletons (gram)	3062 (710)	3121 (681)	3233 (571)
Missing (% (n))	3.4 (92)	0.4 (20)	5.8 (1927)

	Median	Median	Median
	(min–max)	(min–max)	(min–max)

Gravidity	1 (1–14)	1 (1–12)	2 (1–22)
Missing (% (n))	46.1 (1267)	36.0 (1736)	22.9 (7630)
Parity	0 (0–6)	0 (0–10)	0 (0–11)
Missing (% (n))	0.2 (5)	0.04 (2)	0.08 (25)

	Percentage (n)	Percentage (n)	Percentage (n)

Caucasian	55.7 (672)	55.6 (784)	89.6 (490)
Missing (% (n))	56.1 (1541)	80.5 (3782)	98.4 (32 776)
Multiple gestation			
Singleton	67.5 (1853)	43.2 (2031)	79.4 (26 457)
Twin	31.7 (870)	55.9 (2629)	20.6 (6861)
Triplet	0.9 (24)	0.8 (37)	0.02 (5)
Quadruplet	0 (0)	0.04 (2)	0 (0)
Mode of delivery			
Vaginally	30.4 (782)	50.5 (2367)	67.3 (21 316)
Caesarean section	69.6 (1793)	49.5 (2321)	32.7 (10 357)
missing (% (n))	6.3 (172)	0.2 (11)	5.0 (1650)

min = minimum; max = maximum; n = number; NC = naturally conceived; OD = oocyte donation; % = percentage.

**Figure 3.**
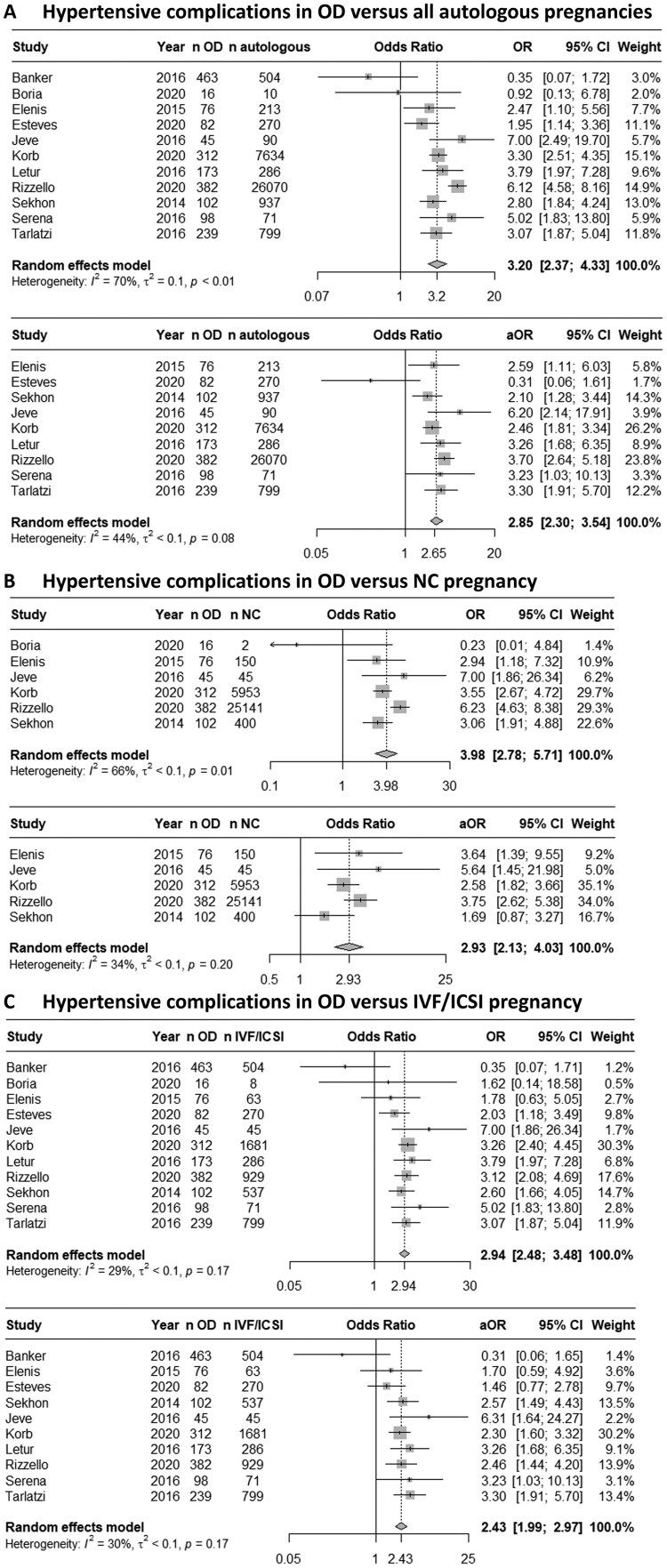
**Results of the two-stage meta-analyses on hypertensive complications in total.** OD = oocyte donation; NC = naturally conceived; n = number; OR = odds ratio; aOR = adjusted odds ratio.

**Figure 4.**
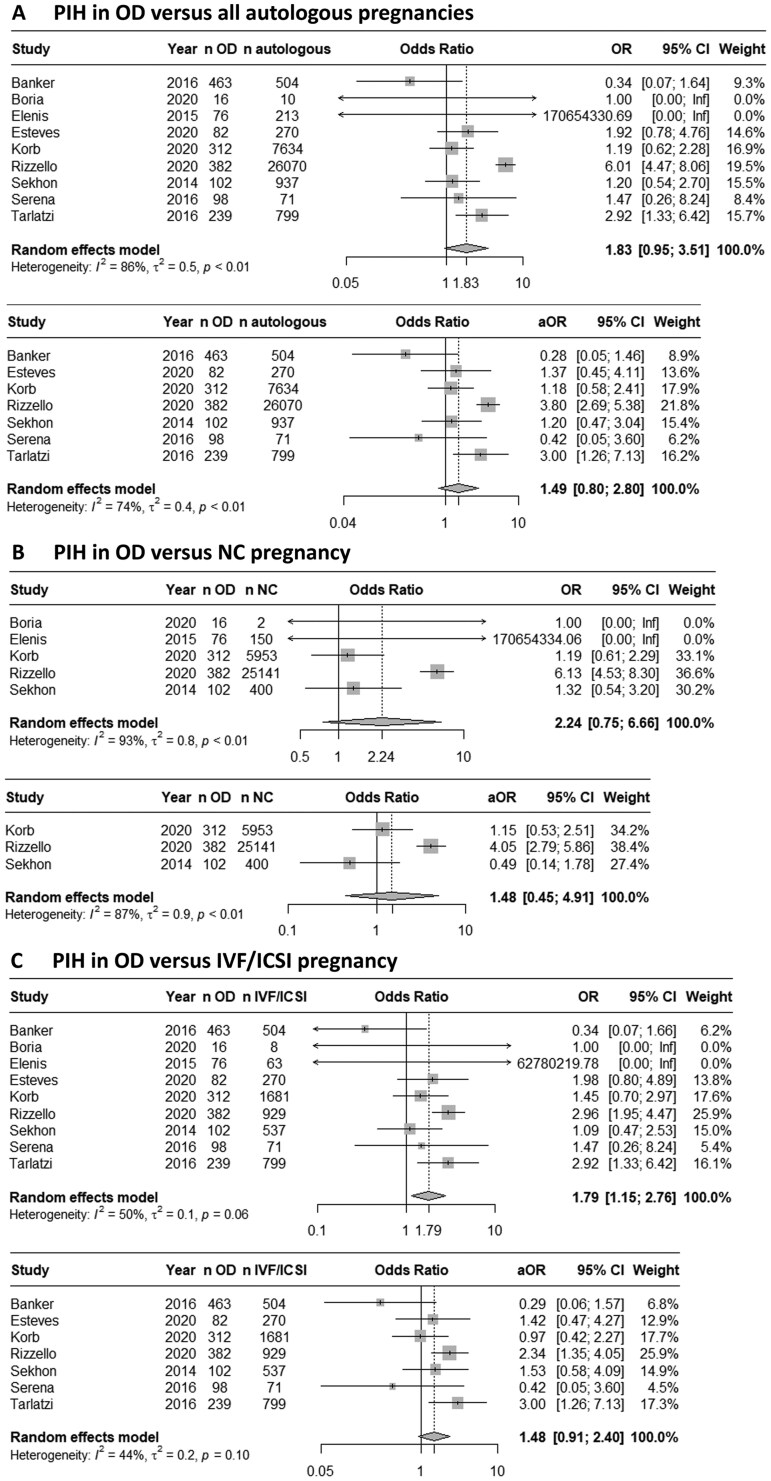
**Results of the two-stage meta-analyses on pregnancy-induced hypertension.** PIH = pregnancy-induced hypertension; OD = oocyte donation; NC = naturally conceived; n = number; OR = odds ratio; aOR = adjusted odds ratio.

**Figure 5.**
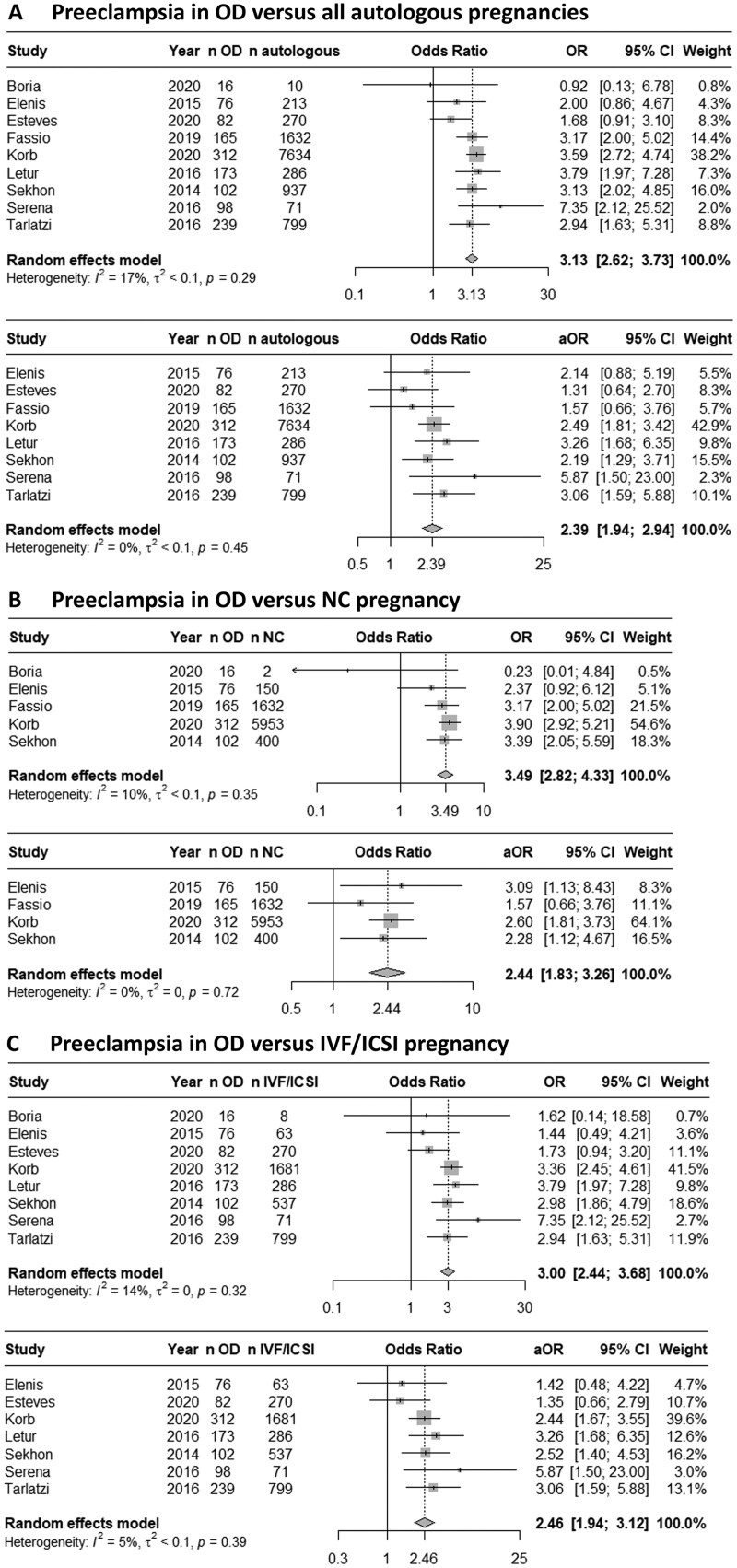
**Results of the two-stage meta-analyses on preeclampsia.** OD = oocyte donation; NC = naturally conceived; n = number; OR = odds ratio; aOR = adjusted odds ratio.

**Figure 6.**
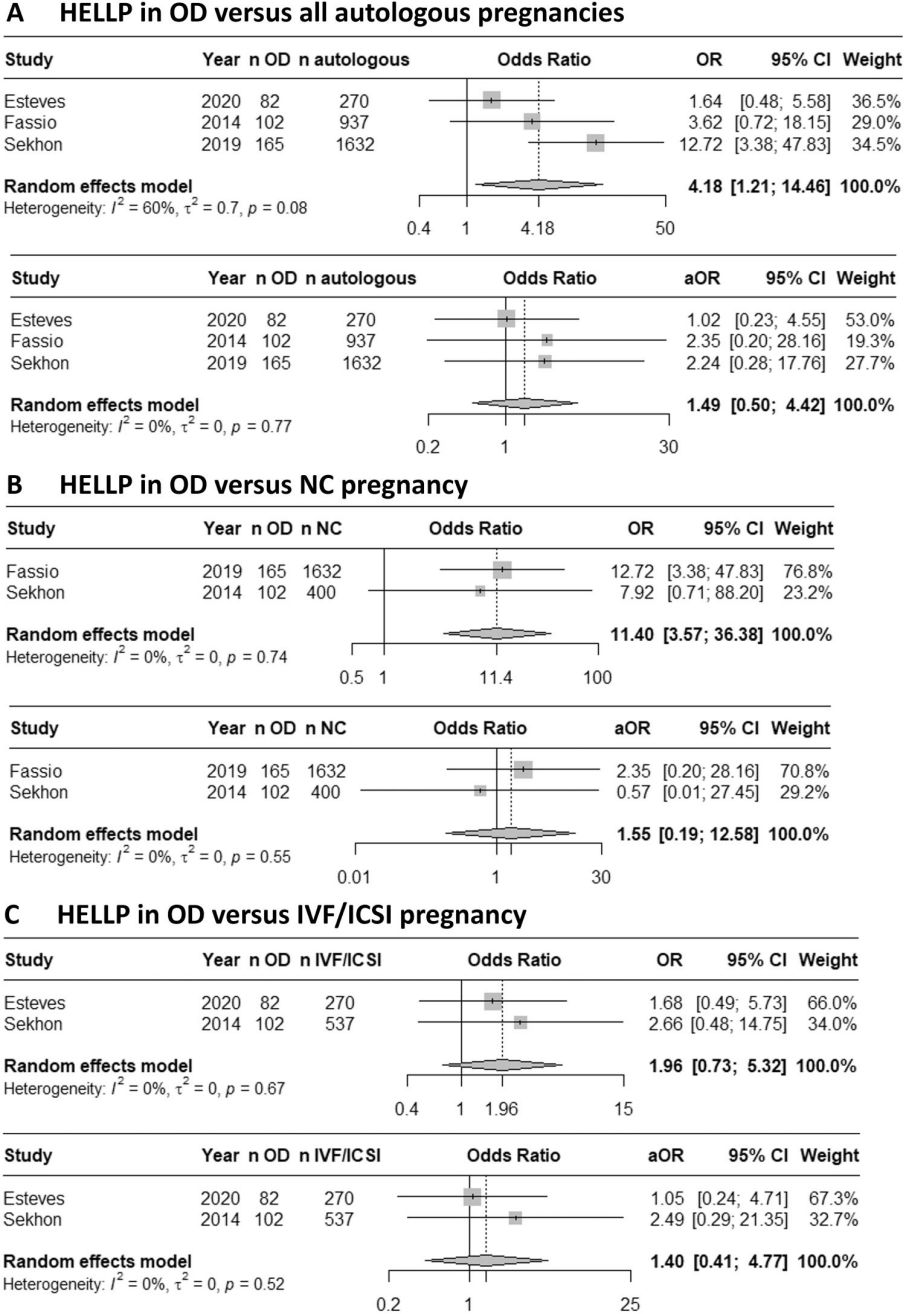
**Results of the two-stage meta-analyses on HELLP syndrome.** HELLP = hemolysis, elevated liver enzymes, and low platelets; OD = oocyte donation; NC = naturally conceived; n = number; OR = odds ratio; aOR = adjusted odds ratio.

Overall, hypertensive complications occurred in 18.2% of OD, 10.9% of IVF/ICSI, and 4.5% of NC pregnancies, with PE accounting for 12%, 7.7%, and 2.2%, respectively. The results of the IPD meta-analyses, using the one-stage approach, are depicted in Table 3. The results of the one-stage approach resemble those from the two-stage approach: the OD pregnancies displayed a significantly higher risk for the development of hypertensive complications (Fig. 3), including PIH (Fig. 4) and PE (Fig. 5), in OD pregnancy compared to both NC and IVF/ICSI pregnancies. The one- and two-stage approach comparing OD to autologous pregnancies showed aORs of respectively 2.62 (95% CI 2.22–3.10) and 2.85 (95% CI 2.30–3.54; *I*^2^ 44%; moderate certainty) for hypertensive complications in total, 2.15 (95% CI 1.73–2.68) and 1.49 (95% CI 0.80–2.80; *I*^2^ 74%; low certainty) for PIH, and 2.28 (95% CI 1.88–2.78) and 2.39 (95% CI 1.94–2.94; *I*^2^ 0%; high certainty) for PE. The results of the two-stage meta-analyses for HELLP syndrome are depicted in Fig. 6, and also show aORs that indicate a higher risk in OD pregnancy, although not significant with broad 95% confidence intervals containing the value 1.

**Table 3.** Results of the individual participant data (IPD) meta-analysis using the one-stage approach.

	n OD	n NC	OR (95% CI)	**aOR** * **(95% CI)**	n OD	n IVF/ICSI	OR (95% CI)	**aOR** * **(95% CI)**	n OD	n autologous	OR (95% CI)	**aOR** * **(95% CI)**
Hypertensive complications	2.385	28.802	4.14 (3.43–5.00)	2.67 (2.15–3.30)	2.385	3.976	2.92 (2.46–3.46)	2.47 (2.04–3.00)	2.472	33.853	3.52 (3.02–4.11)	2.62 (2.22–3.10)
Pregnancy-induced hypertension	2.182	28.958	3.33 (2.56–4.34)	2.10 (1.70–2.60)	2.182	3.657	2.05 (1.57–2.68)	1.77 (1.31–2.40)	2.270	33.694	2.94 (2.33–3.71)	2.15 (1.73–2.68)
Preeclampsia	2.138	5.448	3.44 (2.77–4.26)	2.19 (1.67–2.87)	2.138	3.014	2.97 (2.42–3.63)	2.44 (1.94–3.07)	2.213	9.188	3.09 (2.60–3.67)	2.28 (1.88–2.78)
HELLP syndrome	1.668	2.181	6.95 (2.21–21.81)	1.14 (0.30–4.35)	1.668	1.245	1.98 (0.78–5.00)	1.21 (0.44–3.33)	1.732	3.605	3.21 (1.48–6.94)	1.26 (0.52–3.06)

aOR = adjusted odds ratio; HELLP = hemolysis, elevated liver enzymes and low platelets; n = number; NC = naturally conceived; OD = oocyte donation; OR = odds ratio.

* Adjusted for maternal age, parity, and multiple pregnancy.

### Subgroup analyses

The results of the subgroup analyses are shown in Table 4. Due to a low number (donor-recipient familiar relationship) or high heterogeneity (OD indications) of retrieved IPD, it was not possible to execute all planned subgroup analyses. With regard to the planned subgroup analyses for higher risk of hypertensive complications based on medical history, we did not retrieve sufficient IPD to analyze subgroups based on the presence of renal diseases, systemic lupus erythematosus, and antiphospholipid syndrome. Nevertheless, we did retrieve sufficient IPD to analyze subgroups based on the presence of pre-gestational diabetes and pre-existing hypertension. The subgroup analyses indicated that the risk for hypertensive complications in OD pregnancies remained increased compared to autologous pregnancies, regardless of young maternal age, low BMI, singleton pregnancy, parity, ethnicity, no pre-existent co-morbidities, and only one transferred embryo. These results suggest that OD is an independent risk factor; however, this does not imply that other risk factors are inconsequential. On the contrary, advanced maternal age, high BMI, multiple gestation, and pre-existing co-morbidities are likely to further elevate the absolute risk for hypertensive complications in OD pregnancies. Notably, the risk for hypertensive complications in total was higher in the OD group without the use of acetylsalicylic acid (ASA) or heparin compared to both the autologous and NC control groups. Likewise, the risk for PIH only was higher in the OD group without the use of ASA or heparin compared to the autologous group. However, these findings show wider 95% confidence intervals compared to the original IPD meta-analyses.

**Table 4.** Odds ratios in subgroups using the one-stage approach.

Outcomes		Hypertensive complications			PIH			PE			HELLP syndrome		
Subgroups		*OD vs autologous*	*OD vs NC*	*OD vs IVF/ICSI*	*OD vs autologous*	*OD vs NC*	*OD vs IVF/ICSI*	*OD vs autologous*	*OD vs NC*	*OD vs IVF/ICSI*	*OD vs autologous*	*OD vs NC*	*OD vs IVF/ICSI*
Maternal age	*<35 years*	2.81 (2.09–3.78)	3.16 (2.27–4.40)	2.41 (1.75–3.33)	1.66 (0.98–2.80)	1.80 (1.04–3.13)	1.22 (0.68–2.17)	3.06 (2.19–4.27)	3.16 (2.16–4.61)	2.96 (2.06–4.27)	1.29 (0.21–7.76)	2.87 (0.40–20.69)	0.75 (0.11–5.01)
	*35–40 years*	3.57 (2.73–4.66)	3.93 (2.90–5.32)	3.17 (2.35–4.28)	2.80 (1.86–4.23)	2.69 (1.71–4.25)	2.31 (1.41–3.78)	3.00 (2.18–4.15)	3.40 (2.34–4.94)	2.94 (2.05–4.22)	0.92 (0.18–4.69)	1.77 (0.25–12.38)	0.66 (0.12–3.54)
	*40–45 years*	2.54 (1.97–3.27)	3.05 (2.26–4.12)	2.25 (1.64–3.10)	2.07 (1.45–2.96)	2.31 (1.54–3.47)	1.69 (1.04–2.73)	2.35 (1.68–3.29)	3.00 (1.85–4.87)	2.19 (1.48–3.26)	1.36 (0.28–6.50)	1.15 (0.12–11.24)	1.28 0.21–7.89)
	*>45 years*	2.31 (1.33–4.00)	3.77 (1.59–8.95)	1.54 (0.76–3.13)	4.25 (1.52–1.87)	12.43 (2.08–74.45)	1.40 (0.41–4.76)	1.61 (0.81–3.20)	1.92 (0.53–6.91)	1.55 (0.70–3.40)	–	–	–
Multiple pregnancy	*Singleton*	3.63 (2.91–4.52)	4.50 (3.38–6.00)	2.56 (1.99–3.31)	3.78 (2.79–5.11)	4.19 (2.97–5.91)	2.13 (1.46–3.10)	2.65 (1.98–3.55)	2.36 (1.47–3.78)	2.71 (1.92–3.82)	4.49 (1.24–16.18)	5.87 (1.31–26.18)	2.80 (0.53–14.93
	*Multiplet*	3.20 (2.63–3.88)	3.49 (2.81–4.33)	3.21 (2.58–3.99)	2.08 (1.53–2.83)	2.19 (1.56–3.07)	2.12 (1.48–3.03)	3.23 (2.60–4.03)	3.63 (2.83–4.67)	3.11 (2.44–3.97)	1.96 (0.71–5.39)	2.93 (0.43–19.93)	1.96 (0.68–5.63)
Parity	*Primiparous*	3.43 (2.91–4.04)	3.98 (3.25–4.88)	2.98 (2.49–3.58)	3.07 (2.40–3.93)	3.46 (2.61–4.59)	2.31 (1.73–3.09)	2.87 (2.38–3.46)	3.07 (2.43–3.88)	2.92 (2.35–3.63)	2.82 (1.28–6.22)	5.54 (1.65–18.62)	1.89 (0.73–4.91)
	*Multiparous*	1.55 (1.13–2.13)	2.85 (2.05–3.95)	2.57 (1.77–3.74)	1.86 (1.07–3.22)	2.02 (1.15–3.54)	1.19 (0.62–2.29)	2.83 (1.97–4.07)	2.73 (1.86–4.00)	3.09 (1.99–4.81)	6.02 (0.77–46.83)	10.07 (0.82–123.36)	2.93 (0.24–35.87)
Ethnicity	*Caucasian*	2.63 (1.89–3.68)	2.66 (1.71–4.14)	2.50 (1.74–3.59)	1.39 (0.64–3.04)	1.50 (0.61–3.68)	1.19 (0.52–2.73)	2.87 (1.96–4.21)	2.90 (1.80–4.67)	2.94 (2.07–4.16)	2.51 (0.48–12.97)	5.34 (0.50–56.47)	2.27 (0.43–11.94)
	*Non–Caucasian*	1.90 (1.00–3.62)	2.80 (1.18–6.65)	1.89 (0.94–3.78)	0.63 (0.15–2.65)	1.89 (0.25–4.17)	0.56 (0.14 2.17)	2.61 (1.24–5.50)	2.75 (1.11–6.79)	2.33 (1.18–4.60)	1.24 (0.02–69.61)	0.16 (0.004–6.43)	–
BMI	*<18*	2.37 (0.85–6.60)	2.95 (1.06–8.20)	1.21 (0.37–3.99)	–	–	–	3.20 (0.99–10.33)	3.38 (1.00–11.38)	2.40 (0.66–8.75)	–	–	–
	*18–25*	4.36 (3.61–5.28)	4.98 (4.03–6.16)	3.46 (2.77–4.33)	3.61 (2.68–4.87)	3.36 (0.33–33.72)	2.35 (1.61–3.43)	3.53 (2.84–4.39)	3.92 (3.05–5.03)	3.42 (2.62–4.45)	8.13 (2.51–26.33)	26.61 (3.13–2.27)	3.25 (0.72–14.70)
	*25–30*	3.17 (2.38–4.22)	3.46 (2.54–4.71)	2.38 (1.68–3.36)	2.61 (1.70–4.01)	2.71 (1.72–4.27)	1.51 (0.87–2.62)	3.05 (2.19–4.24)	3.19 (2.22–4.58)	2.88 (1.91–4.33)	2.72 (0.66–11.22)	2.21 (0.47–10.31)	2.75 (0.27–28.19)
	*30–35*	2.97 (1.91–4.64)	3.06 (1.92–4.88)	2.94 (1.72–5.03)	3.70 (2.17–6.30)	4.16 (2.39–7.24)	3.15 (1.50–6.58)	1.85 (1.07–3.56)	1.75 (0.92–3.33)	1.92 (0.95–3.88)	–	–	–
	*>35*	3.71 (1.74–7.94)	3.66 (1.69–7.92)	4.09 (1.55–10.76)	1.03 (0.29–3.55)	1.04 (0.27–3.96)	0.98 (0.16–5.90)	9.37 (3.41–25.73)	9.25 (3.23–26.51)	9.14 (2.86–29.28)	–	–	–
Number of transferred embryos	*1*	20.94 (2.72–161.33)	NA	21.30 (2.75–164.76)	–	NA	–	15.93 (1.97–128.89)	NA	16.38 (2.02–133.16)	–	NA	–
	*≥2*	2.52 (1.75–3.63)	NA	2.36 (1.61–3.44)	1.15 (0.53–2.47)	NA	1.03 (0.46–2.28)	2.95 (2.02–4.30)	NA	2.83 (1.90–4.21)	0.33 (0.04–2.72)	NA	0.30 (0.03–2.76)
Acetylsalicylic acid or heparin use	*No*	4.65* (3.55–6.11)	5.01* (3.74–6.70)	3.13 (2.28–4.29)	4.26* (3.04–5.96)	4.90* (3.42–7.01)	2.33 (1.55–3.50)	3.35 (2.23–5.04)	3.17 (1.92– 5.23)	3.33 (2.14–5.17)	–	–	–
	*Yes*	2.52 (1.64–3.85)	2.58 (1.63–4.08)	1.98 (1.09–3.58)	2.26 (1.47–3.49)	2.70 (1.59–4.56)	1.64 (0.81–3.29)	2.11 (0.68–6.52)	0.91 (0.08–10.39)	2.34 (0.69–7.88)	–	–	–
Pre-gestational diabetes	*No*	3.54 (3.03–4.14)	4.17 (3.45–5.06)	2.94 (2.47–3.49)	2.91 (2.29–3.70)	3.25 (2.47–4.29)	2.08 (1.57–2.74)	1.44 (1.01–2.06)	3.70 (2.89–4.75)	3.06 (2.49–3.76)	–	–	–
	*Yes*	2.65 (1.26–5.57)	3.26 (1.50–7.09)	1.99 (0.83–4.75)	3.87 (1.65–9.07)	4.74 (1.95–11.52)	1.89 (0.65–5.52)	1.24 (0.45–3.39)	1.42 (0.50–4.03)	1.15 (0.37–3.61)	–	–	–
Pre-existing hypertension	*No*	3.83 (3.15–4.66)	4.92 (3.84–6.31)	2.83 (2.28–3.51)	NA	NA	NA	2.57 (1.95–3.39)	2.88 (1.85–4.50)	8.05 (4.03–16.05)	–	–	–
	*Yes*	1.58 (0.65–3.85)	0.33 (0.10–1.08)	2.95 (1.02–8.55)	NA	NA	NA	2.95 (1.05–8.23)	2.09 (0.30–14.51)	3.05 (1.01–9.20)	–	–	–

* Subgroup analyses showing statistical significance, based on no overlapping 95% CI with the original one-stage IPD meta-analysis (see Table 3) results together with a statistical significant interaction term (*P*-value <0.05).

aOR = adjusted odds ratio; HELLP = hemolysis, elevated liver enzymes and low platelets; n = number; NC = naturally conceived; OD = oocyte donation; OR = odds ratio. – = Not enough individual participant data (IPD) available to execute the analysis; NA = Subgroup analysis not applicable.

### Sensitivity analyses

#### Aggregate data meta-analysis

As not all eligible studies were able to share their IPD, we performed a sensitivity analysis by adding the aggregate aORs of these studies together with the two-stage IPD meta-analyses results to check for bias due to missing IPD. Aggregated aORs needed to be adjusted at least for maternal age. In the analysis for all hypertensive complications, the aggregated aORs of four additional studies were added (Malchau *et al.*, 2013; Kennedy *et al.*, 2019; Meyer *et al.*, 2020; Gekka *et al.*, 2021). Furthermore, 8 studies were added to the analysis of PIH (Wolff *et al.*, 1997; Wiggins and Main, 2005; Klatsky *et al.*, 2010; Stoop *et al.*, 2012; Van Dorp *et al.*, 2014; Guilbaud *et al.*, 2017; Dior *et al.*, 2018; Luke *et al.*, 2020), 12 studies for the analysis of PE (Henne *et al.*, 2007; Krieg *et al.*, 2008; Klatsky *et al.*, 2010; Le Ray *et al.*, 2012; Stoop *et al.*, 2012; Malchau *et al.*, 2013; Van Dorp *et al.*, 2014; Nejdet *et al.*, 2016; Rizzo *et al.*, 2016; Guilbaud *et al.*, 2017; Dior *et al.*, 2018; Modest *et al.*, 2019), and 2 additional studies for the analysis of HELLP syndrome (Henne *et al.*, 2007; Guilbaud *et al.*, 2017). The results are shown in Supplementary Data File S3.A, and indicate that the sensitivity analysis for hypertensive complications in total, PE, and HELLP syndrome resembles those from the two-stage IPD meta-analyses. In the analysis for PIH in the OD versus IVF/ICSI group, the results of the sensitivity analysis showed a statistically significant confidence interval, in contrast to the interval shown by the original two-stage meta-analysis.

#### Methodological quality

The IPD of the four studies that were scored as high risk of bias based on the ROBINS-I tool were removed (Simchen *et al.*, 2009; Tranquilli *et al.*, 2013a; Banker *et al.*, 2016; Tarlatzi *et al.*, 2017). The results of all one- and two-stage IPD sensitivity meta-analyses for risk of bias were comparable with the results from all included studies, as depicted in Supplementary Data File S3.B.

#### Publication year

Bubble plots and statistical results are shown in Supplementary Data File S3.C for all analyses, except for HELLP syndrome in OD versus NC and IVF/ICSI pregnancy, as only two studies provided IPD. The ORs and bubble plots do suggest slight increases or decreases in the effect size with each additional year, but these ORs are not statistically significant. Therefore, there is no significant association between the year of publication and the outcome measures when comparing the different study groups.

#### Publication bias

The funnel plots and *P*-values of the Egger’s test are shown in Supplementary Fig. S1. Potential publication bias may be present in all two-stage IPD meta-analyses for PIH, indicating that smaller studies tend to report more negative results than larger studies.

## Discussion

### Summary of evidence and interpretation

The DONOR IPD meta-analysis was realized to generate clinically relevant and robust evidence regarding the development of hypertensive complications, including PIH, PE, and HELLP syndrome, in OD pregnancies compared to autologous NC and IVF/ICSI pregnancies. We were able to retrieve IPD from 16 study cohorts with data from 2747 OD, 4699 autologous IVF/ICSI, and 33 323 NC pregnancies. The one- and two-stage approaches comparing OD to autologous pregnancies resulted in a higher risk for hypertensive complications, including PIH and PE, even after correction for maternal age, parity, and multiple pregnancy. When the autologous group was divided into NC and IVF/ICSI pregnancies, the higher risks in OD pregnancy for hypertensive complications, including PIH and PE, remained. The results of the DONOR IPD meta-analysis provide the most robust evidence until now, as we were able to adjust for confounders and perform subgroup analyses to account for these factors.

To estimate the causal effect of the exposure OD on the development of hypertensive complications in pregnancy, three conditions are required: exchangeability, positivity, and consistency. In this situation, the exposure is unambiguously defined, although the risk of not disclosing the origin of oocytes remains for the non-donor group. Also, the statement of positivity holds, as the patients did or did not develop the outcome. Most challenging is to meet the assumption of exchangeability, which ensures that exchanging the groups (exposed and unexposed) is possible without affecting outcomes. It could also be interpreted as the absence of confounding. As in previous meta-analyses, a considerable amount of bias remains that could influence the studied association; we conducted an IPD meta-analysis. Using IPD increases statistical power, enables adjustment for multiple confounding factors, and improves generalizability. In addition, IPD enables checking the reliability of the data and examining causes for heterogeneity by subgroup analyses. We therefore strongly believe that, as far as possible, we have taken all the assumptions into account.

In addition to the planned subgroup analyses described in the DONOR IPD protocol (van Bentem *et al.*, 2022), we were able to perform subgroup analyses for maternal BMI and number of transferred embryos. No differences in outcome measurements were found in the subgroup analyses for maternal age, BMI, multiple pregnancy, parity, ethnicity, medical history, and number of transferred embryos. These results imply that OD is an independent risk factor for hypertensive complications within these subgroups. Important to note is that, while OD itself contributes to increased risk, additional risk factors, such as advanced maternal age, obesity, and twin pregnancy, may further increase the risk for hypertensive complications in OD pregnancies.

An important hypothesis underlying the increased risk of hypertensive complications in OD pregnancies is the immunological mechanism due to higher fetal-maternal immunogenetic dissimilarity, as the child inherits paternal and donor-derived genes. Reasonably, the maternal immune system needs to react differently to accept the allogeneic fetus, as is supported by previous research (van der Hoorn *et al.*, 2014; Lashley *et al.*, 2015; van Bentem *et al.*, 2020). A shared immunological hypothesis has been described in other clinical scenarios associated with a higher risk of PE, including first pregnancies, use of donor semen, long interpregnancy intervals, change of partner (Rambaldi *et al.*, 2019) and gestational surrogacy (Söderström-Anttila *et al.*, 2016).

A remarkable finding from subgroup analyses is the potential lower risk for hypertensive complications when ASA or heparin is used during OD pregnancy. Nevertheless, the available data remains heterogeneous. Some cohorts reported exclusively on ASA (Tranquilli *et al.*, 2013a) or heparin (Sekhon *et al.*, 2014 (enoxaparin 40–80 mg/day during pregnancy)), while others prescribed ASA in combination with heparin (Serena *et al.*, 2019; Boria *et al.*, 2020 (ASA 100 mg/day plus enoxaparin 40 mg/day during pregnancy; Rizzello *et al.* 2020)). These differences likely reflect local practice variation as well as heterogeneous indications for prophylaxis, such as standard use in IVF protocols, history of thrombosis, thrombophilia, antiphospholipid syndrome, or prevention of PE. Although our results suggest a potential protective effect of ASA and/or heparin, the variability in agents, dosages, timing, and underlying indications hampers definitive conclusions. Previously, the ASPRE trial showed a reduction of PE with the use of ASA (Rolnik *et al.*, 2017), though data from a subgroup analysis of OD pregnancies from the ART pregnancy cohort were missing. Therefore, it remains unclear whether the administration of ASA and/or heparin directly contributes to a reduced incidence of PE in OD pregnancy, or if the observed decrease primarily results from enhanced risk identification within this population, leading to ASA prophylaxis and earlier induction of labor prior to disease onset.

Due to an insufficient number of IPD, it was not possible to complete the planned subgroup analysis for donor-recipient familiar relationship, for the indication of OD, or to execute the planned time-to-event analysis. The lack of baseline characteristics of the oocyte donors, and whether the oocytes originated from related donors (e.g. sisters or relatives) or unrelated donors (e.g. anonymous or via oocyte banks), causes restrictions in the performed analyses. Future studies should therefore aim to report donor characteristics, also including clinic-specific allocation protocols.

Because of high heterogeneity, resulting in low group numbers, of retrieved IPD for OD indication, we were not able to perform this subgroup analysis. The latest European Society of Human Reproduction and Embryology (ESHRE) guideline on premature ovarian insufficiency (POI) mainly focuses on pregnancy outcomes in women who developed POI after cancer therapy, indicating similar risks to women with normal ovarian function (Panay *et al.*, 2025). However, in women with non-iatrogenic POI, underlying biological, cardiovascular, immunological, and possibly genetic disturbances may increase the risk of pregnancy complications, potentially affecting neonatal health. POI may reflect premature oocyte aging from accumulated DNA damage (Ruth *et al.*, 2021), possibly leading to higher rates of chromosomal abnormalities, miscarriage, and birth defects (Laven, 2020). Furthermore, delayed diagnosis of idiopathic POI often leads to unrecognized cardiovascular decline. POI is a known risk factor for cardiovascular disease, and affected women may be more susceptible to hypertensive complications in pregnancy (Roeters van Lennep *et al.*, 2016). Additionally, the frequent overlap with autoimmune disease, such as Addison’s disease, autoimmune thyroiditis, and type 1 diabetes (Domniz and Meirow, 2019), and unrecognized genetic syndromes with obstetric relevance (Heddar *et al.*, 2022) may further increase the risks for pregnancy complications. These additional risk factors associated with POI highlight the need to investigate the risk of hypertensive complications according to OD indication.

Additionally, it would have been interesting to perform a subgroup analysis according to the approach of endometrial preparation for frozen embryo transfer. Recently, several observational studies consistently indicate a higher risk for PE in hormonal replacement therapy cycles compared to ovulatory cycles (Ginström Ernstad *et al.*, 2019; von Versen-Höynck *et al.*, 2019; Asserhøj *et al.*, 2021; Roelens *et al.*, 2022). Possibly, the absence of the corpus luteum in these artificial cycles is related to the development of hypertensive complications. Indeed, the corpus luteum produces vasoactive agents next to progesterone and estrogen, implying an important role in placentation (Singh *et al.*, 2020). For our study, we did not receive enough IPD on the endometrial preparation approach to perform a subgroup analysis, possibly because most women who apply for OD do not exhibit ovulatory cycles. To investigate the effect of this possible confounder, future studies should include only patients with ovulatory cycles, for example, patients who turn to the reception of oocytes from their partner, or patients with inherited diseases as an indication for OD.

For the different IPD meta-analyses, we included the two unexposed groups, NC and non-donor IVF/ICSI pregnancies, to minimize confounding, as ART is associated with a higher risk for obstetric complications (Chih *et al.*, 2021). IVF/ICSI pregnancies have a comparable assisted reproductive technique as performed in OD, though the hormonal treatment is different. In non-donor IVF/ICSI, the women receive hormonal treatment both for the retrieval of the oocyte and before (fresh) embryo transfer. With OD, the donor receives the hormonal treatment for oocyte retrieval, and the recipient only receives hormonal treatment for embryo transfer. However, the disadvantage of analyzing the unexposed groups separately is the decreased number of patients and consequently statistical power. Therefore, we also investigated the incidence of hypertensive complications in OD pregnancies compared to all autologous pregnancies.

In the DONOR IPD meta-analyses, the one- and two-stage approaches generally yielded consistent results across the studied complications and comparison groups. The two-stage approach in the analyses of PIH showed the same trends as the one-stage approach, though with a broader 95% CI, possibly due to lower amounts and publication bias of IPD. Likewise, the aORs in both the one- and two-stage approach for HELLP syndrome in OD pregnancy also showed values towards a higher risk, though due to broad 95% CIs, definite conclusions are not possible (Altman and Bland, 1995).

### Strengths and limitations

Because of the retrieved IPD, we were able to execute meta-analyses adjusted for the most important confounding factors. Furthermore, it was possible to perform various subgroup, sensitivity and meta-regression analyses to explore potential sources of heterogeneity and assess the robustness of the study results. However, despite these strengths, the use of IPD still encounters certain limitations. Firstly, results always remain dependent on how many researchers are willing and able to provide IPD, which could lead to bias. This bias was investigated by a sensitivity analysis using available aggregate data from studies that did not provide IPD, yet not all studies reported clear and adjusted aggregated outcome measures, also limiting this sensitivity analysis. Despite the availability of IPD, the limitations in the quality of primary studies persisted. To address this issue, a sensitivity analysis was conducted to assess the impact of risk of bias, and a meta-regression analysis was performed with a focus on publication year. When a sufficient amount of IPD was obtained, we planned to conduct time-to-event analysis using Kaplan-Meier survival curves to examine the relationship between the mode of conception and the time until the development of hypertensive complications. However, due to insufficient IPD, it was not possible to execute this analysis.

### Implications for clinical practice and future research

We believe that the results of our DONOR IPD meta-analysis will increase alertness of health care professionals that are involved in OD health care towards the risk profile of these pregnancies. For patients in clinical practice, this means that OD pregnancies have about 2.6 times the risk of hypertensive complications, including a 2.2 times higher risk of PIH and a 2.3 times higher risk of PE, compared to autologous pregnancies. Currently, none of the worldwide used guidelines of the National Institute for Health and Clinical Excellence (NICE), the American College of Obstetricians and Gynecologists (ACOG), the International Society for the Study of Hypertension in Pregnancy (ISSHP), or the International Federation of Gynecology and Obstetrics (FIGO) indicate OD as a risk factor for hypertensive complication in pregnancy (Redman, 2011; Tranquilli *et al.*, 2014, 2020; Poon *et al.*, 2019). The DONOR IPD meta-analysis results in the best evidence-based statement so far for international guidelines in obstetrics. In future research, the DONOR IPD will be used to externally validate a model to predict the development of PE in OD pregnancies, which could work as a support tool for the management of OD pregnancies in medical practice (Lafeber *et al.*, 2024). Moreover, the results of this DONOR IPD meta-analysis support further research on the underlying mechanisms of the development of hypertensive complications in OD pregnancies. This topic is part of our ongoing prospective, multicenter DONOR study (van Bentem *et al.*, 2019), in which we hypothesize that the fetal-maternal immunogenetic dissimilarity plays an important role in this development. Moreover, we aim to elucidate the effect of preventive treatment, such as low-dose acetylsalicylic acid, on the development of hypertensive complications, specifically in an OD cohort.

## Conclusion

The DONOR IPD meta-analysis provided a unique opportunity to assess the risk for hypertensive complications in OD pregnancies compared to autologous pregnancies. With using both one- and two-stage meta-analysis approaches, corrected for the most important confounders maternal age, parity and multiple gestation, we found an increased risk for hypertensive complications, including PIH and PE, in OD pregnancies. Supplementary subgroup analyses, taking maternal age, BMI, ethnicity, pre-existing hypertension and diabetes, parity, multiple gestation, and number of transferred embryos into account, also highlighted the independent effect of OD on the development of hypertensive complications. A potential lower risk when using acetylsalicylic acid or heparin during OD pregnancy compared to control groups was noted. These results should increase awareness of hypertensive complications in OD pregnancy among health care professionals, and consequently, patients considering OD should be informed about the increased risks before the start of treatment.

## Supplementary Material

dmag006_Supplementary_Data

## Data Availability

The investigators (or their institutions) who shared the IPD for the DONOR IPD meta-analysis maintain ownership of their IPD. All requests for access to the IPD should be made directly to these investigators. The data dictionary and R code for this IPD meta-analysis are available on request.
