# Supplementary material for: The risk for the development of hypertensive complications in oocyte donation pregnancy: a systematic review and individual participant data meta-analysis (DONOR IPD)
Source: Hum Reprod Update. 2026 Mar 31;32(4):488–508. doi: 10.1093/humupd/dmag006 (PMC13319335; doi:10.1093/humupd/dmag006)
Supplement: dmag006_Supplementary_Data [file dmag006_supplementary_data.docx]

# Supplementary Data

## Manuscript title

The risk for the development of hypertensive complications in oocyte donation pregnancy: a systematic review and individual participant data meta-analysis (DONOR IPD)

## Authors

Kim van Bentem^1^, Marie-Louise van der Hoorn^1^, Manish Banker^2^, Maria de la Calle^3^, Evangelia Elenis^4,5^, Dina El Demellawy^6^, Nathan S Fox^7^, Yadava Jeve^8^, Diane Korb^9^, Hélène Letur^10,11^, Yoav Yinon^12^, Antonio Farina^13,14^, Francesca Rizzello^15^, Kenny A Rodriguez-Wallberg^16^, Michal Simchen^17^, Serena Simeone^18^, Theoni Tarlatzi^19^, Stefano Giannubilo^20^, Saskia Le Cessie^21^, Eileen Lashley^22^

^1^ Department of Gynecology and Obstetrics, Leiden University Medical Center, Leiden, the Netherlands.

^2^ Banker IVF & Women's Hospital, Ahmedabad, India.

^3^ Department of Obstetrics and Gynecology, La Paz Hospital, Madrid, Spain.

^4^ Department of Women’s and Children’s Health, Uppsala University Hospital, Uppsala, Sweden.

^5^ Reproductive Center, Women's Clinic, Uppsala University Hospital, Uppsala, Sweden.

^6^ Department of Pediatric Pathology, Children’s Hospital of Eastern Ontario, Faculty of Medicine, University of Ottawa, Ottawa, Ontario, Canada.

^7^ Department of Obstetrics, Gynecology, and Reproductive Science, Mount Sinai School of Medicine, New York, USA.

^8^ Birmingham Women's and Children's Hospital, Birmingham, United Kingdom.

^9^ Department of Obstetrics and Gynecology, Robert Debré Hospital, Assistance Publique-Hôpitaux de Paris, Paris, France.

^10^ Department of Obstetrics and Gynecology, Reproductive Medicine and Fertility Preservation, Foch Hospital, Suresnes, France.

^11^ Department of Reproductive Medicine and Fertility Preservation, Pau Pyrénées Polyclinic, Pau, France.

^12^ Department of Obstetrics and Gynecology, Sheba Medical Center, Tel-Hashomer, Faculty of Medical and Health Sciences, Tel Aviv University, Tel Aviv, Israel.

^13^ Obstetric Unit, IRCCS Azienda Ospedaliero-Universitaria di Bologna, Bologna, Italy.

^14^ Department of Medical and Surgical Sciences (DIMEC), Alma Mater Studiorum-University of Bologna, Bologna, Italy.

^15^ Assisted Reproductive Technology Centre, Careggi University Hospital Florence, Italy.

^16^ Department of Reproductive Medicine, Division of Gynecology and Reproduction, Karolinska University Hospital & Karolinska Institutet, Stockholm, Sweden.

^17^ Department of Obstetrics and Gynecology, Sheba Medical Center, Tel-Hashomer, Tel Aviv University, Tel Aviv, Israel.

^18^ Department of High Risk Pregnancy, Careggi University Hospital, Florence, Italy.

^19^ Fertility Clinic, Department of Obstetrics and Gynecology, Erasme Hospital, Université Libre de Bruxelles, Brussels, Belgium.

^20^ Department of Clinical Sciences, Università Politecnica delle Marche, Ancona, Italy.

^21^ Department of Clinical Epidemiology, Leiden University Medical Center, Leiden, the Netherlands.

^22^ Department of Gynecology and Obstetrics, Erasmus Medical Center, Rotterdam, the Netherlands.

# Table of contents

[**Supplementary Data File S1. Search terms** 3](#_Toc205213587)

[**Supplementary Table S1. Study characteristics of all eligible studies** 5](#_Toc205213588)

[**Supplementary** **Data File S2. Risk of Bias** 11](#_Toc205213589)

[1. Risk of bias according to the ROBINS-I tool. 11](#_Toc205213590)

[2. Detailed clarification of the risk of bias per included study according to the validation checklist developed by Scholten *et al*. 13](#_Toc205213591)

[**Supplementary Data File S3.** **Sensitivity and meta-regression analyses** 65](#_Toc205213592)

[A. Sensitivity analyses – Addition of aggregated data 65](#_Toc205213593)

[B. Sensitivity analyses – Methodological quality 67](#_Toc205213594)

[C. Meta-regression analyses - Year of publication 69](#_Toc205213595)

[1. Hypertensive complications in total 69](#_Toc205213596)

[2. Pregnancy-induced hypertension 70](#_Toc205213597)

[3. Preeclampsia 71](#_Toc205213598)

[4. HELLP 72](#_Toc205213599)

[**Supplementary Figure S1. Publication bias** 73](#_Toc205213600)

# Supplementary Data File S1. Search terms

PubMed

("Oocyte Donation"[Mesh] OR "Oocyte Donation"[tw] OR "Oocyte Donations"[tw] OR "Ovum Donation"[tw] OR "Ovum Donations"[tw] OR "Egg Donation"[tw] OR "Egg Donations"[tw] OR "Embryo Disposition"[Mesh] OR "Embryo Disposition"[tw] OR "Embryo Abandonment"[tw] OR "Embryo Donation"[tw] OR "Embryo Donations"[tw]) AND ("Hypertension, Pregnancy-Induced"[Mesh] OR "Pregnancy-Induced Hypertension"[tw] OR "Pregnancy Induced Hypertension"[tw] OR "Gestational Hypertension"[tw] OR "Pregnancy Transient Hypertension"[tw] OR "Eclampsia"[tw] OR "Eclampsias"[tw] OR “HELLP Syndrome"[tw] OR "Pre-Eclampsia"[tw] OR "Pre Eclampsia"[tw] OR "Preeclampsia"[tw] OR "Pregnancy Toxemia"[tw] OR "Pregnancy Toxemias"[tw] OR "Edema-Proteinuria-Hypertension Gestosis"[tw] OR "Edema Proteinuria Hypertension Gestosis"[tw] OR "Toxemia of Pregnancy"[tw] OR "Toxemia Of Pregnancies"[tw] OR "EPH Complex"[tw] OR "EPH Gestosis"[tw]) AND (("Pregnancy"[Mesh] OR "Pregnancy"[tw] OR "Pregnancies" OR "Gestation"[tw] OR "Gestations"[tw]) OR ("Fertilization in Vitro"[Mesh] OR "Fertilization in Vitro"[tw] OR "Fertilizations in Vitro"[tw] OR "In Vitro Fertilization"[tw] OR "In Vitro Fertilizations"[tw] OR "Test-Tube Fertilization"[tw] OR "Test Tube Fertilization"[tw]) OR ("Sperm Injections, Intracytoplasmic"[Mesh] OR "Intracytoplasmic Sperm Injection"[tw] OR "Intracytoplasmic Sperm Injections"[tw] OR "ICSI"[tw]) OR ("Insemination, Artificial"[Mesh] OR "Artificial Insemination"[tw] OR "Artificial Inseminations"[tw]))

*March 2024: 180*

Embase

(exp oocyte donation/ OR "Oocyte Donation".mp. OR "Oocyte Donations".mp. OR "Ovum Donation".mp. OR "Ovum Donations".mp. OR "Egg Donation".mp. OR "Egg Donations".mp. OR exp embryo disposition/ OR "Embryo Disposition".mp. OR "Embryo Abandonment".mp. OR "Embryo Donation".mp. OR "Embryo Donations".mp.) AND (exp maternal hypertension/ OR "maternal hypertension".mp. OR "Pregnancy-Induced Hypertension".mp. OR "Pregnancy Induced Hypertension".mp. OR "Gestational Hypertension".mp. OR "Pregnancy Transient Hypertension".mp. OR exp "eclampsia and preeclampsia"/ OR "Eclampsia".mp. OR "Eclampsias".mp. OR "HELLP Syndrome".mp. OR "Pre-Eclampsia".mp. OR "Pre Eclampsia".mp. OR "Preeclampsia".mp. OR exp pregnancy toxemia/ OR "Pregnancy Toxaemia".mp. OR "Pregnancy Toxaemias".mp. OR "Pregnancy Toxemia".mp. OR "Pregnancy Toxemias".mp. OR "Edema-Proteinuria-Hypertension Gestosis".mp. OR "Edema Proteinuria Hypertension Gestosis".mp. OR "Toxemia of Pregnancy".mp. OR "Toxemia Of Pregnancies".mp. OR "Toxaemia of Pregnancy".mp. OR "Toxaemia Of Pregnancies".mp. OR "EPH Complex".mp. OR "EPH Gestosis".mp.) AND (exp pregnancy/ OR "Pregnancy".mp. OR "Pregnancies" OR "Gestation".mp. OR "Gestations".mp. OR exp in vitro fertilization/ OR "in vitro fertilisation".mp. OR "Fertilization in Vitro".mp. OR "Fertilizations in Vitro".mp. OR "In Vitro Fertilization".mp. OR "In Vitro Fertilizations".mp. OR "Fertilisation in Vitro".mp. OR "Fertilisations in Vitro".mp. OR "In Vitro Fertilisation".mp. OR "In Vitro Fertilisations".mp. OR "Test-Tube Fertilization".mp. OR "Test Tube Fertilization".mp. OR "Test-Tube Fertilisation".mp. OR "Test Tube Fertilisation".mp. OR exp intracytoplasmic sperm injection/ OR "Intracytoplasmic Sperm Injection".mp. OR "Intracytoplasmic Sperm Injections".mp. OR "ICSI".ti,ab. OR exp artificial insemination/ OR "Artificial Insemination".mp. OR "Artificial Inseminations".mp.)

*March 2024: 381*

Cochrane

("Oocyte Donation" OR "Oocyte Donations" OR "Ovum Donation" OR "Ovum Donations" OR "Egg Donation" OR "Egg Donations" OR "Embryo Disposition" OR "Embryo Disposition" OR "Embryo Abandonment" OR "Embryo Donation" OR "Embryo Donations"):ti,ab,kw AND ("Pregnancy Induced Hypertension" OR "Gestational Hypertension" OR "Pregnancy Transient Hypertension" OR Eclampsia OR "HELLP Syndrome" OR "Pre Eclampsia" OR preeclampsia OR "Pregnancy Toxemia" OR "Edema Proteinuria Hypertension Gestosis" OR "Toxemia of Pregnancy" OR "EPH Complex" OR "EPH Gestosis"):ti,ab,kw AND ("Pregnancy" OR "Pregnancies" OR "Gestation" OR "Gestations" OR "Fertilization in Vitro" OR "Fertilizations in Vitro" OR "In Vitro Fertilization" OR IVF OR "In Vitro Fertilizations" OR "Test Tube Fertilisation" OR "Test Tube Fertilization" OR "Intracytoplasmic Sperm Injection" OR "Intracytoplasmic Sperm Injections" OR "ICSI" OR "Artificial Insemination" OR "Artificial Inseminations"):ti,ab,kw

*March 2024: 2 trials*

# Supplementary Table S1. Study characteristics of all eligible studies

| **Study** | **Journal** | **Country** | **De-sign** | **Study period** | **Inclusion criteria** | **Exclusion criteria** | **Control group** | **Participants (n)** | **Mean maternal age (yrs)** | **HDP outcome** | **Definition of outcome** |
| --- | --- | --- | --- | --- | --- | --- | --- | --- | --- | --- | --- |
| Banker  2016 | J Hum Reprod Sci | India | PC | 2014 | All women who conceived after embryo transfer following IVF/ICSI with or without OD | Not stated | Fresh embryo transfer using self‑oocytes and thaw embryo transfer using vitrified‑warmed embryos | 691 fresh IVF; 611 fresh OD; 810 thawed IVF | Fresh IVF: 30.68 ±3.65; Fresh OD: 36.65 ±5.18; Thawed IVF: 32.54 ±5.04 | PIH, PE | Not stated |
| Barnard  2019 (conf abstract) | J Reprod Med | United States | RC | 2005-2014 | OD recipients and matched women undergoing autologous oocyte IVF | Not stated | OD and autologous IVF | 27 OD  27 IVF | OD: 41.5±4.8  IVF: 40.3±2.3 | PIH, PE | Not stated |
| Boria  2020 | J Matern Fetal Neonatal Med | Spain | RC | 2012-2016 | Twin OD pregnancies delivered after 24 weeks of gestation | Not stated | Autologous IVF twin pregnancies with a due date two months before or after that of the case | 50 OD  50 IVF | OD: 40 (38.7-43.2)  IVF: 36 (34-40) | PIH, PE | PIH = systolic >140 or diastolic blood pressure >90 after 20 weeks; PE = PIH with proteinuria >300mg/24h |
| Dior  2018 | Arch Gynecol Obstet | Israel | RC | 1995-2012 | OD singleton live births of parturients of ≥45 years of age. | Women who conceived by IVF without OD | NC singleton live births of parturients of ≥45 years of age | 135 OD  270 NC | OD: 47.8 ±2.6 (45–58)  NC: 45.7 ±0.94 (45–49) | PIH, PE | PIH = systolic ≥140 mmHg and/or diastolic blood pressure ≥90 mmHg after 20 weeks; PE = PIH with proteinuria |
| Elenis  2015 | BMC Pregnancy Childbirth | Sweden | RC | 2005-2008 | Singleton OD pregnancies | Women who did not speak or read Swedish | 1. Age-matched nulliparae with singleton NC pregnancies and no history of subfertility  2. Heterosexual women with singleton autologous IVF pregnancies | 76 OD  150 NC  63 IVF | OD: 35.0 (25-43)  NC: 34.0 (19-36)  IVF: 33.0 (25-39) | PIH, PE, HELLP, eclampsia | Definitions according to the ICD-10 |
| Esteves  2020 | Placenta | Canada | RC | 2011-2018 | All clinical cases of OD patients with a placental pathology report and complete antenatal history | OD pregnancies without placental pathology report and no relevant clinical antenatal history | ART cases who did not achieve pregnancy through OD | 85 OD  270 ART | OD: 41.53 ±6.26 (28-55)  ART: 35.30 ±4.47 (23-50) | PIH, PE, HELLP, eclampsia | Not stated |
| Fassio  2019 | J Clin Med | Italy | RC | 2008-2019 | Singleton OD pregnancies >24 weeks, with complete data at delivery | Abortions and  therapeutic interruptions of pregnancy | NC singleton pregnancy  occurring in a woman without pre-existing, systemic and localized diseases | 296 OD  1407 NC | OD: 44 (31–56)  NC: 31 (15–49) | PIH, PE, HELLP | PIH = hypertension during pregnancy in previously normotensive patients |
| Gekka  2021 | Hypertens Pregnancy | Japan | RC | 2013-2017 | OD pregnancies of women aged 40 years or above at delivery | Multifetal preg-nancies, preexistent hypertension, unknown HDP outcome | An autologous IVF and NC group of women aged 40 years or above at delivery | 44 OD  484 IVF  833 NC | OD: 47 yrs (IQR: 45–48)  IVF: 41 yrs (IQR: 40–42)  NC: 41 yrs (IQR: 40–42) | PIH, PE, eclampsia | PIH = blood pressure ≥140/90 mmHg; PE = PIH with proteinuria ≥0.3 g/day; Eclampsia = generalized seizures in a context of severe PE |
| Guilbaud  2017 | Fertil Steril | France | RC | 2010-2014 | All women with OD twin pregnancies who gave birth after 24 weeks of gestation | Women transferred from other maternity units due to maternal or fetal disease; monochorionic, monoamniotic twins | Autologous IVF, and non-IVF twin pregnancies | 102 OD  201 IVF  369 non-IVF | OD: 43.1 ±5.1 (26–59)  IVF: 34.6 ±3.9 (24–51)  Non-IVF: 32.7 ±5.3 (17–50) | PIH, PE, HELLP | PIH = systolic ≥140 mmHg and/or diastolic blood pressure ≥90 mmHg after 20 weeks; PE = hyper-tension with proteinuria ≥300 mg/24h |
| Hayashi  2019 (conf abstract) | J Obstet Gynaecol Res | Japan | RC | Not stated | Women pregnant through OD over 40 years old | Not stated | NC and ART pregnancies of women over 40 years old | 13 OD  52 ART  94 NC | OD: 43-52  NC, ART: un-known, >40 yrs | HDP, unspecified | Not stated |
| Henne 2007 | J Reprod Med | USA | RC | 1997 –2002 | Conception through OD at any IVF center and delivery at Lucille Packard Children’s Hospital | Not stated. | Women >38 years who conceived with autologous oocytes and delivered in the same hospital and period | 69 OD  681 autologous controls | OD: 45.28 ±3.63 (36.77-55.78)  Controls: 41.60 ±1.37 (38.03-48.55) | PE, HELLP | Not stated |
| Jackson  2015 | Fertil Steril | United States | RC | 2000-2010 | First ART pregnancies of women ≥45 years old | Twins and higher order pregnancies | First NC pregnancies of women ≥45 years old. | 185 ART (64 autologous, 120 OD)  193 NC | OD: 47.1 ± 2.2  Autologous ART: 46.7 ±2.0  NC: 45.6 ±0.1 | PIH, PE | Not stated |
| Jeve  2016 | Int J Gynaecol Obstet | UK | RC | 2007-2014 | OD pregnancy and delivery of a live neonate after 24 weeks at a teaching hospital in Leicester in the period | Pregnancies after preimplantation genetic diagnosis and surgical sperm retrieval or use of donor sperm | Age-matched IVF and NC pregnancies | 45 OD  45 IVF  45 NC | OD: 40.23 ±5.64 IVF: 39.23 ±1.71 NC: 39.69 ±0.71 | PIH, PE | PIH = blood pressure >140/90 mmHg after 20 weeks; PE= PIH with proteinuria ≥0.3 g/day |
| Keegan  2007 | Fertil Steril | USA | RC | 1999-2003 | OD pregnancies of patients <35 years and ≥40 years of age | Triplets, frozen embryo transfer, cycles monitored at program satellite offices | Autologous IVF pregnancies in the same age ranges | 19 OD <35 296 IVF <35 171 OD ≥40 192 IVF ≥40 | OD<35: 31.7 ±0.4 IVF<35: 31.0 ±0.2 OD≥40: 43.9 ±0.2 IVF≥40: 41.4 ±0.1 | PIH | PIH = high blood pressure during pregnancy |
| Kennedy  2019 | Hum Reprod | Austra-lia | RC | 2009-2017 | Donated gamete pregnancies delivering after 20 weeks’ gestation | Miscarriage, delivery before 20 weeks, multiple pregnancies | Autologous IVF pregnancies delivering after 20’ weeks gestation. | 578 OD  1,435 donor sperm  239 ED  13,191 IVF | OD: 42.8 (38.5, 45.8); Donor sperm: 37.0 (33.7, 39.5); ED: 42.0 (37.0, 45.8); IVF: 35.1 (32.3, 38.0) | PIH, PE | PIH = high blood pressure after 20 weeks in absence of other pathology or PE; PE = high blood pressure and evidence of multisystem dysfunction after 20 weeks |
| Klatsky 2010 | Obstet Gynecol | USA | RC | 1998-2005 | Singleton and twin OD pregnancies resulting in live birth from both fresh and cryopreserved cycles | Monozygotic twins and if outcome data were not reported | Age- and plurality-matched IVF pregnancies | 77 OD  81 IVF | OD: 40.2 ±3.5  IVF: 39.8 ±4.1 | PIH, PE | PIH = systolic >140 mmHg or diastolic blood pressure >90 mmHg; PE = PIH with proteinuria (>300 mg/24h or ≥1+ protein urine specimen) |
| Korb  2020 | Hum Reprod | France | PC | 2014-2015 | Twin pregnancies ≥22 weeks of gestation, conceived after assisted reproduction | Unknown mode of conception | NC twin pregnancies ≥ 22 weeks of gestation  in the JUMODA cohort | 329 OD  5,890 NC  854 non-IVF  1,307 IVF  368 ICSI | OD: 40.5 ±4.9  NC: 30.6 ±5.1  Non-IVF: 31.7 ±4.5  IVF: 33.6 ±4.7  ICSI: 32.6 ±4.4 | SAMM, PE, HELLP, Eclampsia | PE = hypertension ≥140/90 and proteinuria ≥0.3 g/24 h |
| Koren  2021 (conf abstract) | Fertil Steril | Israel | RC | 2000-2018 | OD singleton, twin and triplet pregnancies | Not stated | IVF and NC singleton, twin and triplet pregnancies | 831 OD  1,926 IVF  467,401 NC | Singletons:  OD: 43 ±4.9  IVF: 34.1 ±4.7  NC: 31.3 ±5.3 | PIH | Not stated |
| Krieg  2008 | Fertil Steril | USA | RC | 2001-2005 | OD treatment at Stanford Infertility Center and delivery at Lucille Packard Children’s Hospital | Not stated | Women >38 years old with autologous IVF in the same center and delivery at the same hospital | 71 OD  108 IVF | OD: 42.7 ±4.40 (30.7-53.0)  IVF: 41.3 ±1.84 (38.0-47.2) | PE | Not stated |
| Le Ray  2012 | Human Reprod | France | RC | 2008-2010 | Women >43 years old giving birth after OD | Not stated. | Autologous IVF and non-IVF pregnancies | 236 non-IVF  40 IVF  104 OD | Non-IVF: 44.1 ±1.4  IVF: 44.0 ±1.4  OD: 46.2 ±2.9 | PE | PE = blood pressure ≥140/90 mmHg and proteinuria ≥0.3 g/24 |
| Letur  2016 | Fertil Steril | France | RC | 2005-2012 | Singleton OD pregnancies from seven ART centers | Multiple pregnancies, OD treatment abroad | Singleton autologous IVF/ICSI pregnancies at the same center | 217 OD  363 controls | OD: 34.4 ±9.4 Controls: 34.2 ±4.5 | PIH, PE, eclampsia | PIH = blood pressure ≥140/90 mmHg after 20 weeks; PE = repeated ≥140/90 mmHg, with proteinuria ≥0.3 g/day; Eclampsia = generalized seizures in a context of severe preeclampsia (repeated ≥160/110 mmHg, proteinuria ≥3 g/day). |
| Levron  2014 | Am J Obstet Gynecol | Israel | RC | 2005-2011 | Singleton OD pregnancies with prenatal and delivery care at a tertiary medical center | Congenital or chromosomal abnormalities, multiple pregnancies | Women >38 years with autologous IVF pregnancy in same time period, delivery at the same center | 139 OD  126 IVF | OD: 45 (23-57) IVF: 41 (38-46) | PIH, PE | PIH = blood pressure ≥140/90 mmHg after 20 weeks; PE = PIH with proteinuria (≥300 mg/24 hours or 2+ dipstick); HDP = presence of PIH or PE |
| Luke  2020 | Am J Obstet Gynecol | United States | RC | 2004-2013 | Women in eight States who underwent IVF resulting in a live birth after pregnancy, categorized by oocyte source (autologous vs OD) and embryo state (fresh vs thawed) | Multiple pregnancies, sperm donation and women in the non-IVF group with an indication of infertility treatment. | 10:1 sample of births from non-IVF singleton deliveries | 11,309 OD  72,273 IVF  1,382,311 non-IVF | OD: 42.2 ±4.6  IVF: 35.2 ±4.2  Non-IVF: 28.6 ±5.9 | PIH, PE | PIH = blood pressure ≥140 mmHg systolic or ≥90 mmHg diastolic after 20 weeks; PE = PIH with proteinuria (≥300 mg/24h, or protein/ creatinine ratio ≥0.3, or urine protein dipstick 1+) |
| Malchau  2013 | Fertil Steril | Den-mark | RC | 1995-2010 | OD pregnancies from the Danish IVF register, both singletons and twins | OD abroad | Age- and year-of-birth-matched IVF, ICSI and NC pregnancies | 375 OD  17,592 IVF  8,967 ICSI  33,852 NC | Singletons:  OD: 36.8 ±5.2  IVF: 34.0 ±4.0  ICSI: 33.2 ±4.0  NC: 30.2 ±4.8 | PIH, PE | HDP = chronic hypertension, PIH, and/or PE; No definition on PIH and PE stated |
| Meyer  2020 | RBMO | Israel | RC | 2011-2018 | Singleton OD pregnancies >24 weeks’ gestation of women aged 45-47 years who gave birth at a tertiary medical center | Unknown conception mode, multiple gestation, ovulation induction, fetal reduction | Singleton autologous IVF and NC pregnancies >24 weeks’ gestation of women aged 45-47 years who gave birth at a tertiary medical center | 159 OD  68 IVF  73 NC | OD: 46.00 (45.54-46.42)  IVF: 45.56 (45.25-46.20)  NC: 45.47 (45.15-45.94) | PIH, PE | PIH = blood pressure ≥140 mmHg systolic or ≥90 mmHg diastolic after 20 weeks; PE = PIH with proteinuria (≥300 mg/24h, or protein/ creatinine ratio ≥0.3, or urine protein dipstick 1+) |
| Modest  2019 | J Assist Reprod Genet | United States | RC | 2000-2015 | OD deliveries at or after 20 weeks’ gestation from a large tertiary care hospital | <18 years of age, IVF cycles ending in ectopic pregnancy, miscarriage, or induced abortion | Autologous IVF and non-IVF deliveries | 262 OD  3,501 IVF  65,321 non-IVF | OD: 42.3 (39.1–45.1)  IVF: 35.6 (32.6–38.8)  Non-IVF: 31.9 (28.6–35.0) | PE, ischemic placental disease | PE = blood pressure ≥140/90 during delivery admission, and either PE symptoms, seizures, or abnormal laboratory values before delivery |
| Nejdet  2016 | Acta Obstet Gynecol Scand | Sweden | RC | 2003-2012 | Singleton OD pregnancies after fresh and thawed cycles | Multiple pregnancies | Singleton IVF, ICSI and NC pregnancies | 388 OD  26,696 IVF/ICSI  999,804 NC | Split categories:  OD 16.5% ≥40 yrs  IVF/ICSI 8.5% ≥40 yrs  NC 3.5% ≥40 yrs | PE, eclampsia | PE and eclampsia were defined according to the International Classification of Diseases 10 codes O.14/O.15 |
| Rizzello  2020 | RBMO | Italy | RC | 2009-2017 | Singleton OD pregnancies of women giving birth at the University Hospital of Careggi, Florence | Multiple pregnancies | Singleton IVF/ICSI and NC pregnancies | 276 OD  925 IVF/ICSI  24,650 NC | OD: 43.5 ±4.5  IVF/ICSI: 35.6 ±4.3  NC: 32.8 ±5.4 | PIH, PE | HDP included chronic hypertension and *de novo* hypertension, either PIH or PE |
| Rizzo  2016 | Ultra-sound Obstet Gynecol | Italy | PC | 2007-2014 | Nulliparous singleton pregnancies achieved by IVF with autologous or donor oocyte | No ultrasound recordings, pre-existing maternal diseases, lack in follow-up | Nulliparous singleton NC pregnancies | 109 OD  307 IVF  498 NC | OD: 41.4 (36-50)  IVF: 39.9 (31-43)  NC: 32.3 (19-42) | PE | PE was defined according to the guidelines of the International Society for the Study of Hypertension in Pregnancy (2001) |
| Rodriguez-Wallberg  2019 | Gynecol Endo-crinol | Sweden | PC | 2007-2014 | Women who achieved singleton pregnancies and live births using donor oocytes after IVF with or without ICSI | Women aged 40 yrs or older at time of IVF treatment or having medical conditions | Women undergoing IVF/ICSI with autologous oocytes, matched (1:2) by age, IVF or ICSI, and year of embryo transfer | 259 OD  515 IVF/ICSI | OD: 35.0 ±3.79  IVF/ICSI: 34.8 ±3.61 | PIH, PE, HELLP | Definitions according to the ICD-10 |
| Rudenko  2020 | Pathobiology | Russia | RC | Not stated | Women whose pregnancy occurred as a result of IVF with a donor oocyte in a surrogate motherhood program or OD. | Severe extragenital pathologies, pregnancy complications with well-studied pathogenesis | Autologous IVF pregnancies | 42 OD  47 SM  21 IVF | OD: 42.7 ±3.91  SM: 29.4 ±3.19  IVF: 33.97 ±4.10 | PIH, PE | Not stated |
| Salha  1999 | Hum Reprod | England | RC | 1992-1997 | Conception through donated gametes (donor sperm, OD, ED) | Patients with pre-existing medical condition that might predispose to the develop-ment of PE | Conception with autologous gametes, matched for age, parity and demographic background | 22 OD  33 IUI with donor sperm  12 ED  27 controls | OD: 38.1 (27-42)  OD controls: 37.6  Don sperm: 30.9  ED: 36.7 | PIH, PE | PIH = blood pressure ≥140/90 mmHg after 20 weeks or diastolic >20 mm Hg above booking level; PE = PIH with proteinuria ≥0.5 g/day after 20 weeks |
| Sekhon  2014 | Fertil Steril | United States | RC | 2005-2013 | OD twin pregnancies | Women aged >50 years, monochorionic-monoamniotic placentation | Age-matched autologous IVF twin pregnancies | 56 OD  56 IVF | OD: 43.0 ±6.0  IVF: 41.9 ±1.7 | PIH, PE | PIH = systolic of ≥140 mmHg or diastolic blood pressure of ≥90 mmHg after 20 weeks; PE = PIH plus proteinuria (≥300 mg/24h) |
| Serena  2019 | Minerva Ginecol | Italy | RC | 2011-2017 | Singleton OD pregnancies referred at a High-Risk Pregnancy Unit, gestational age ≥24 weeks at delivery | Sperm or double donation, multiple pregnancies, abortion <24 wks | Singleton IVF and NC pregnancies that gave birth at the same time | 290 OD  290 IVF  870 NC | OD: 43.4 ±2.9  IVF: 37.7 ±2.4  NC: 33.6 ±5.5 | PIH, PE | PIH = systolic ≥140 mmHg and diastolic blood pressure ≥90 mmHg after 20 week; PE = PIH with proteinuria ≥3 g/24h |
| Shrim  2010 | J Perinat Med | Canada | RC | 2001-2007 | Singleton OD pregnancies | No matched control, teenage pregnancies | Singleton autologous pregnancies matched by age and time of delivery | 20 OD  60 control | OD: 38.3  Controls: 38.4 | PIH | Not stated |
| Simchen  2009 | Hum Reprod | Israel | RC | 1999-2008 | Twin OD pregnancies of women of 40 years or older | Not stated | Singleton OD pregnancies of similar women, and all women carrying twins in 2007 | 83 OD singleton  42 OD twin  417 control twin | OD singleton: 49.3 ±4.7; OD twin: 49.2 ±4.3  Controls: 31.6 ±6.5 | PIH | PIH = systolic ≥140 mmHg or diastolic blood pressure ≥90 mmHg after 20 weeks |
| Sites  2017 | Fertil Steril | United States | RC | 2005-2010 | Singleton and twin OD pregnancies at >20 weeks of gestation | Data from Connecticut and Michigan | Autologous ART singleton and twin pregnancies | 1,696 OD  14,241 ART | Singleton, fresh:  OD: 41.78 ±4.35  ART: 35.28 ±4.08 | PIH, PE, eclampsia | Definitions according to the ICD-9 |
| Söderström-Anttila  1998 | Human Reprod | Finland | RC | 1992-1996 | Women with OD conception, who delivered a liveborn or stillborn infant at ≥24 weeks or ≥500 g | Not stated | Autologous IVF pregnancies, delivered at ≥24 weeks | 51 OD  97 IVF | OD: 33.5 ±4.7  IVF 33.4 ±3.7 | PIH, PE | PIH = blood pressure ≥140/90 mmHg after 20 weeks; PE = PIH with proteinuria ≥0.5 g/day |
| Stoop  2012 | Reprod Biol Endo-crinol | Belgium | RC | 1999-2008 | OD pregnancies resulting in offspring after more than 20 weeks of gestation | Preimplantation genetic diagnosis, testicular sperm extraction or use of donor sperm | Matched autologous IVF pregnancies, conceived in the same period and center | 148 OD  148 IVF | OD: 36.3 ±4.5  IVF: 36.2 ±4.5 | PIH, PE | PIH = blood pressure ≥140/90 mmHg after 20 weeks. PE = PIH with proteinuria ≥0.3 g/day |
| Tarlatzi  2017 | RBMO | Belgium | RC | 1991-2013 | Singleton OD pregnancies, delivery after more than 22 weeks of gestation | Turner syndrome, multiple pregnancies, testicular sperm extraction,  preimplantation genetic diagnosis,  cryopreserved embryos | Singleton IVF/ICSI pregnancies, delivery after more than 22 weeks of gestation at the same hospital during the same period | 144 OD  144 IVF/ICSI | Both groups: 35.64 ±4.54 (22-43) | PIH, PE | PIH = blood pressure ≥140/90 mmHg after 20 weeks of gestation; PE = PIH with proteinuria ≥0.3g/day after 20 weeks of gestation |
| Tranquilli  2013 | J Matern Fetal Neonatal Med | Italy | RC | Not stated | ICSI pregnancies using heterologous oocytes | Not stated | Homologous ICSI and NC pregnancies in women >40 years | 26 OD  52 ICSI  52 NC | OD: 42.7 (28-52) ICSI: 37.5 (29-47)  NC: 41.5 (40-45) | PIH, PE | PIH = systolic ≥140 mmHg and/or diastolic blood pressure ≥90 mmHg after 20 weeks; PE = PIH with proteinuria ≥300 mg/day or urine protein/ creatinine ratio ≥30 mg/mmol |
| Van Dorp  2014 | Eur J Obstet Gynecol Reprod Biol | Nether-lands | RC | 1992-2009 | All women who underwent OD treatment in the Erasmus MC Medical Centre | Cycles without embryo transfers | Matched autologous IVF subjects | 110 OD  311 IVF | OD: 36.8 (32.5-39.2)  IVF 37.2 (33.5-39.1) | PIH, PE | PIH = diastolic blood pressure ≥90 mm Hg. PE = PIH with proteinuria (>300 g of protein  in the urine per day). |
| Wiggins  2005 | Am J Obstet Gynecol | USA | RC | 1999-2004 | OD pregnancies | Not stated | Autologous IVF pregnancies | 50 OD  50 IVF | OD: 37.7 ±3.6 (31-50)  IVF: 41.9 ±5.1 (30-45) | PIH, PE | PIH = systolic ≥140 mmHg or diastolic blood pressure ≥90 mmHg after 20 weeks; = PIH with proteinuria ≥300 mg/day |
| Wolff  1997 | Obstet Gynecol | USA | RC | 1988-1996 | OD pregnancies | Not stated | NC pregnancies of women ≥38 years | 46 OD  49 NC | OD 41.5 ±1.8  NC 42.7 ±2.2 | PIH | Not stated |
| Woo  2017 | Fertil Steril | United States | RC | 1990-2014 | Gestational surrogate pregnancies from commissioned embryos | Missing data on outcome, multiples, multifetal selective reduction, vanishing twin | All antecedent NC pregnancies of the included women | 182 surrogacy  312 NC | Surrogacy: 33.0 ±4.7  NC: - | PIH, PE | Not stated |
| Yadav  2022 | J Reprod Infertil | India | RC | 2011-2017 | All women between 20-45 year who conceived through OD | OD with siblings as donors prior to this period | Spontaneous conception in the same time period | 102 OD  306 NC | OD: 35.13 ±5.03  NC: 31.75 ±4.47 | PIH, PE | Not stated |
| Yamada  2015 (conf abstract) | Int J Gynaecol Obstet | Japan | RC | 2014 | Pregnancies achieved through infertility treatments (e.g. IUI, IVF/ICSI, OD) | Cases of stillbirth, multiple pregnancy, unsure treatment | NC pregnancies | 1,373 pregnancies in total | Not stated | PIH | Not stated |
| Zeman  2022 (conf abstract) | Fertil Steril | Argenti-na | RC | 2019-2021 | OD pregnancies, that performed combined first trimester screening for PE, FGR and aneuploidies | Multiple pregnancies, high aneuploidies risk, ART in other IVF center | IVF and NC pregnancies, that performed combined first trimester screening for PE, FGR and aneuploidies | 64 ART  362 NC | 42.1 fresh OD  43.5 frozen OD  36.8 fresh IVF  34.5 frozen IVF  33.3 NC | PE | Not stated |

RC = retrospective cohort study; PC = prospective cohort study; ART = assisted reproductive technique; OD = oocyte donation; ED = embryo donation; IVF/ICSI = in vitro fertilization/intracytoplasmic sperm injection; NC = naturally conceived; ART = assisted reproductive technique; SM = surrogate motherhood; PIH = pregnancy induced hypertension; PE = preeclampsia; HELLP = hemolysis, elevated liver enzymes, and low platelets; HDP= Hypertensive disorders of pregnancy; SAMM = severe acute maternal morbidity, defined as any of the following: maternal death, severe postpartum hemorrhage i.e. transfusion ≥4 units of packed red blood cells, uterine artery embolisation, vascular ligation, compressive uterine suture, emergency peripartum hysterectomy, postpartum hemorrhage requiring second line therapy, eclampsia, HELLP syndrome with admission to an intensive care unit, pre-eclampsia (defined as hypertension ≥140/90 and proteinuria ≥0.3 g/24 h) only if it induced preterm delivery for a main maternal indication before 32 gestational weeks; AMA = advanced maternal age; IQR = interquartile range; n = number; yrs = year

# Supplementary Data File S2. Risk of Bias

1. Risk of bias according to the ROBINS-I tool. Conf abstr = conference abstract.


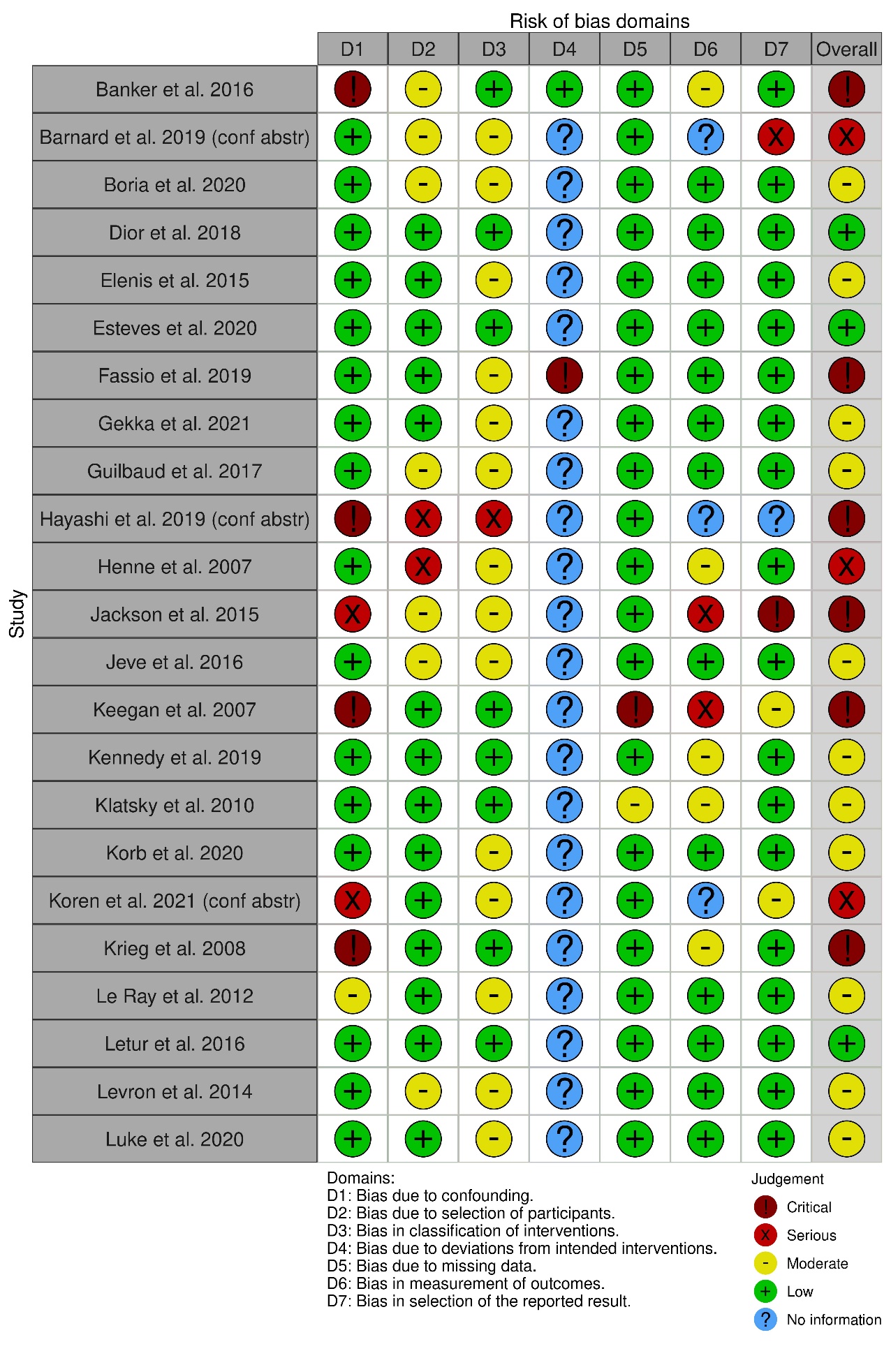


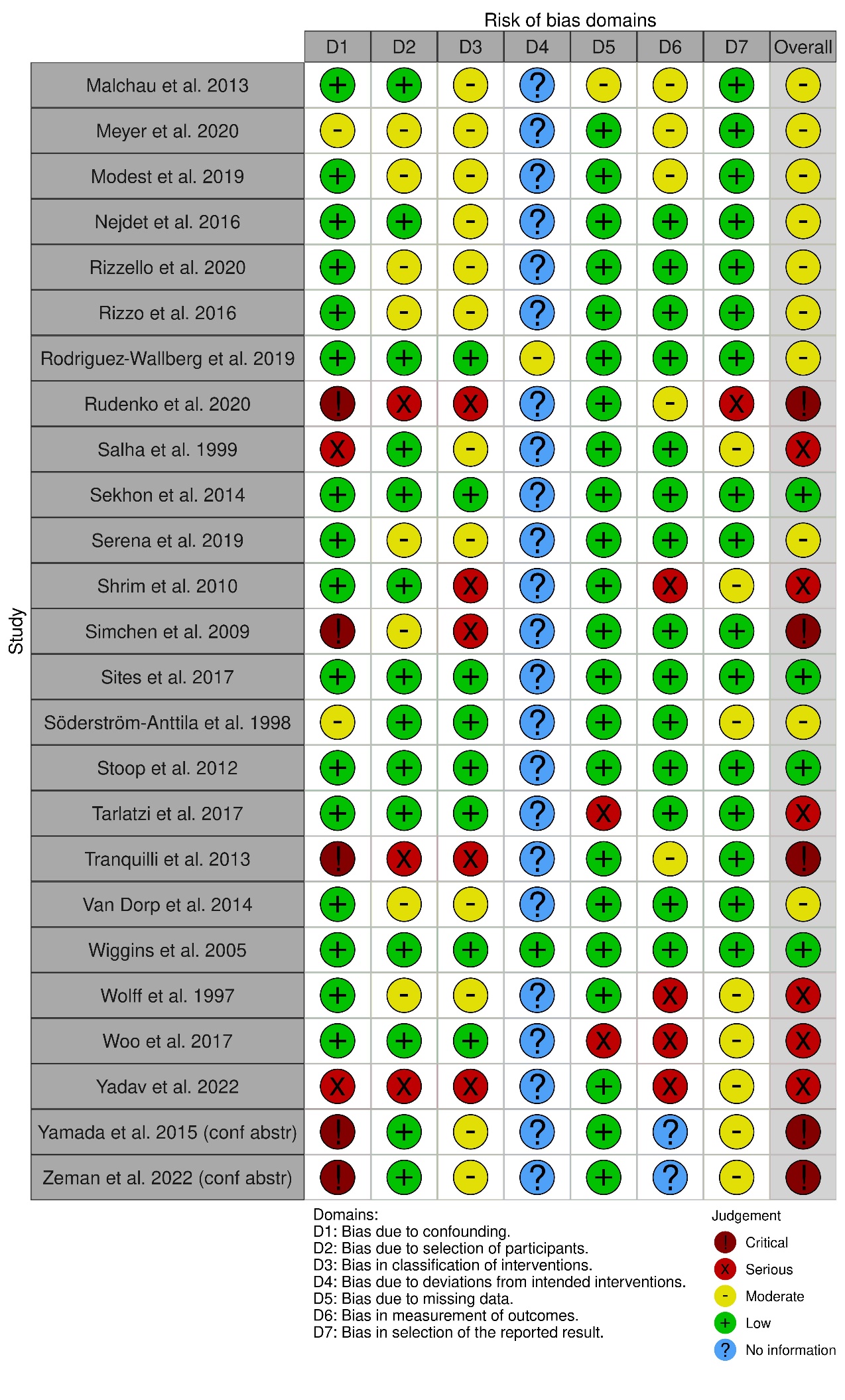


## 2. Detailed clarification of the risk of bias per included study according to the validation checklist developed by Scholten *et al*.

| **Banker, *et al.* 2016** | | |
| --- | --- | --- |
| **Bias due to confounding** | | |
| **Confounding factor** | **Adjusted** | **Explanation** |
| Maternal age | No |  |
| Multiple gestation | No |  |
| Other | No |  |
| **Information bias** | | |
| **Information of interest** | | **Acquired by** |
| Exposure: mode of conception | | All the women who conceived following an embryo transfer at the study center during the study duration were included. |
| Outcome: hypertensive disorders of pregnancy | | No definition of the outcome stated. All the patients were provided the same antenatal care and were advised delivery at a well‑equipped obstetric setup at the fertility center. However, obstetric care after 12 weeks was provided by an obstetrician of their choice as this was not provided at the fertility facility. Hence, there is no information whether women pregnant after OD were equally treated as the IVF women.  Two approaches were used to collect the data:  • At the time of referral, each obstetrician was provided with a form for pregnancy monitoring and to record the outcome. The form was sent through the post and with the patient. In addition, the same was communicated to the respective obstetrician by telephone. They were requested to complete the form and send to the center within a month of abortion, ectopic pregnancy, or delivery;  • In cases where the obstetrician could not be contacted, the details regarding outcome were obtained from the subjects themselves through a phone call. |
| **Selection bias** | | |
| Selection of participants | | The study included all the patients who had a positive pregnancy test after embryo transfer carried out between January 1, 2014, and December 31, 2014, following IVF/ICSI in one infertility center. |
| Loss of follow-up or missing data | | No loss of follow-up or missing data. |

| **Barnard, *et al.* 2019 (conference abstract)** | | |
| --- | --- | --- |
| **Bias due to confounding** | | |
| **Confounding factor** | **Adjusted** | **Explanation** |
| Maternal age | Yes | Patients were matched on age (+/- 5 years). |
| Multiple gestation | Yes | Patients were matched on plurality. |
| Other | Yes | Patients were matched on parity. |
| **Information bias** | | |
| **Information of interest** | | **Acquired by** |
| Exposure: mode of conception | | Database of Mayo Clinic in Rochester, Minnesota. However, the IVF cohort could have contained women pregnant after OD that did not reveal the origin of their conception. |
| Outcome: hypertensive disorders of pregnancy | | No definition or collection method of the outcome is stated. There is no information whether women pregnant after OD were equally treated as women pregnant with autologous IVF pregnancies. |
| **Selection bias** | | |
| Selection of participants | | OD recipients who delivered at twenty weeks of gestation or greater were matched to women who underwent IVF with autologous oocytes at Mayo Clinic in Rochester, Minnesota, from 2005 to 2014. |
| Loss of follow-up or missing data | | No loss of follow-up or missing data. |

| **Boria, *et al.* 2020** | | |
| --- | --- | --- |
| **Bias due to confounding** | | |
| **Confounding factor** | **Adjusted** | **Explanation** |
| Maternal age | Yes | A multivariate analysis with logistic regression models was conducted in order to adjust for maternal age. |
| Multiple gestation | Yes | Only twin pregnancies were included. |
| Other | No |  |
| **Information bias** | | |
| **Information of interest** | | **Acquired by** |
| Exposure: mode of conception | | Women were classified into two groups: OD and autologous IVF. However, the IVF cohort could have contained women pregnant after OD that did not reveal the origin of their conception. |
| Outcome: hypertensive disorders of pregnancy | | PIH is defined as systolic blood pressure >140 or diastolic blood pressure >90 after 20 weeks of pregnancy; PE is defined as PIH with proteinuria >300mg/24h. Clinical records were reviewed for the registration of social and demographic characteristics, obstetrical and perinatal complications. There is no information whether women pregnant after OD were equally treated as women pregnant with autologous IVF pregnancies. |
| **Selection bias** | | |
| Selection of participants | | Participants were included from the Department of Obstetrics in the University Hospital La Paz, a tertiary care referral center in Madrid, Spain. Data were collected from 50 consecutive women with twin pregnancies after OD who delivered after 24 weeks of gestation from 1 January 2012 to 31 December 2016 in University Hospital La Paz. For every case, a control was selected among woman with a twin pregnancy after autologous IVF with a due date within 2 months before or after that of the case. |
| Loss of follow-up or missing data | | No loss of follow-up or missing data. |

| **Dior, *et al.* 2018** | | |
| --- | --- | --- |
| **Bias due to confounding** | | |
| **Confounding factor** | **Adjusted** | **Explanation** |
| Maternal age | Yes | Multivariate logistic regression models were performed controlling for possible confounders, including maternal age. |
| Multiple gestation | Yes | Only singleton pregnancies were included. |
| Other | Yes | Multivariate logistic regression models were performed controlling for possible confounders, including parity. |
| **Information bias** | | |
| **Information of interest** | | **Acquired by** |
| Exposure: mode of conception | | The population was divided by the mode of conception into two groups: OD and natural conception. A comparison ratio of 1:2 was used between the study group and the control group. Data concerning fertility treatments were further confirmed through the infertility outpatient clinic of each participating hospital. |
| Outcome: hypertensive disorders of pregnancy | | PIH is defined by systolic blood pressure ≥140 mmHg and/or diastolic blood pressure ≥90 mmHg on at least two occasions, 4h apart, developing after 20 weeks’ gestation in previously normotensive women in the absence of proteinuria. PE is defined by systolic blood pressure ≥140 mmHg and/or diastolic blood pressure ≥90 mmHg with proteinuria of ≥300 mg in 24h, or two readings of at least ++ on dipstick analysis of midstream or catheter urine specimens if no 24h collection was available. Data were retrieved from the delivery room management software. There is no information whether women pregnant after OD were equally treated as women pregnant with NC pregnancies. |
| **Selection bias** | | |
| Selection of participants | | Singleton live births of parturients ≥ 45 years of age at four major hospitals in Jerusalem, Israel was conducted. Women who conceived IVF without OD were excluded from the study. Data were collected during the years 1995–2012. |
| Loss of follow-up or missing data | | No loss of follow-up or missing data. |

| **Elenis, *et al.* 2015** | | |
| --- | --- | --- |
| **Bias due to confounding** | | |
| **Confounding factor** | **Adjusted** | **Explanation** |
| Maternal age | Yes | Multivariable regression analyses were adjusted for maternal age (<35, ≥35 years). |
| Multiple gestation | Yes | Only singleton pregnancies were included. |
| Other | Yes | Multivariable regression analyses were adjusted for BMI (<25 or ≥25 kg/m2), nicotine Use (Yes/No), gestational age (weeks), and chronic diseases (Yes/No). A sensitivity analysis for parity was executed. |
| **Information bias** | | |
| **Information of interest** | | **Acquired by** |
| Exposure: mode of conception | | All OD and IVF pregnancies were included from the same study cohort. The medical information on fertility treatments was retrieved from medical records at each hospital. However, the time frame and community of the NC pregnancies is not stated. Therefore, the NC cohort could have contained women pregnant after OD that did not reveal the origin of their conception. |
| Outcome: hypertensive disorders of pregnancy | | PIH, PE, eclampsia, HELLP syndrome were defined according to the ICD-10. All medical data analyzed were retrieved from the Swedish Medical Birth Register, a Swedish population-based register. There is no information whether women pregnant after OD were equally treated as women pregnant with non-donor pregnancies. |
| **Selection bias** | | |
| Selection of participants | | The present study is part of the “Swedish multicenter study on gamete donation”, a cohort study of donors and recipients of donated gametes receiving treatment at seven fertility clinics performing donation treatment in Sweden. During the period 2005–2008, consecutive couples starting donation treatment were approached regarding participation. The Index group comprises of women who conceived a singleton pregnancy through OD. Women who did not speak and/or read Swedish were excluded. Two control groups were included: Nulliparous women (Control group A) with NC singleton pregnancies, singleton deliveries and no history of subfertility found in the medical register; and heterosexual women who conceived singleton pregnancies using autologous IVF treatment at one of the same seven hospitals. |
| Loss of follow-up or missing data | | No loss of follow-up or missing data. |

| **Esteves, *et al.* 2020** | | |
| --- | --- | --- |
| **Bias due to confounding** | | |
| **Confounding factor** | **Adjusted** | **Explanation** |
| Maternal age | Yes | Multivariable regression was used, adjusting for maternal age. |
| Multiple gestation | Yes | Multivariable regression was used, adjusting for type of gestation. |
| Other | Yes | Multivariable regression was used, adjusting for parity and BMI. |
| **Information bias** | | |
| **Information of interest** | | **Acquired by** |
| Exposure: mode of conception | | All OD and IVF pregnancies were included from the same study cohort. Antenatal records from the department of Maternal Fetal Medicine (MFM) clinic database of multiples, MFM clinic database of ART patients, and ART patient data retrieved from the BORN NIDAY Perinatal database were used to search for inclusions. |
| Outcome: hypertensive disorders of pregnancy | | No definition stated. Data was collected from medical records. There is no information whether women pregnant after OD were equally treated as women pregnant with ART pregnancies. |
| **Selection bias** | | |
| Selection of participants | | High risk pregnancies achieved by OD and non-OD-ART between October 1, 2011 and June 1, 2018 who delivered at The Ottawa Hospital in Canada and had a placental pathology report and complete antenatal history, were included. As well, twice the number of control autologous IVF cases were included. The study excluded patients who received OD, but had no placental pathology report and those who had no relevant clinical antenatal history i.e., missing antenatal records. |
| Loss of follow-up or missing data | | No loss of follow-up or missing data. |

| **Fassio, *et al.* 2019 (overlapping cohort with Masturzo *et al.* 2019)** | | |
| --- | --- | --- |
| **Bias due to confounding** | | |
| **Confounding factor** | **Adjusted** | **Explanation** |
| Maternal age | Yes | Multivariate regression analysis adjusted for maternal age. |
| Multiple gestation | Yes | Only singleton pregnancies included. |
| Other | Yes | Multivariate regression analysis adjusted for BMI, parity and comorbidities. |
| **Information bias** | | |
| **Information of interest** | | **Acquired by** |
| Exposure: mode of conception | | OD pregnancies were identified in the hospital’s discharge database and in computerized records of pregnant women referred to the Prenatal Screening Center, where information on type of conception is mandatorily recorded. Low-risk pregnancies were selected from the TOCOS archives. In this study, low-risk pregnancy is defined as a spontaneous singleton pregnancy occurring in a woman without pre-existing, systemic and localized diseases. However, as OD is only legal from 2014 in Italy and even after women go abroad due to restrictions, the NC cohort could have contained women pregnant after OD that did not reveal the origin of their conception. |
| Outcome: hypertensive disorders of pregnancy | | No clear definition stated, only that PIH is defined as hypertension during pregnancy in previously normotensive patients. Data was collected from medical records and the TOCOS database. There is no information whether women pregnant after OD were equally treated as women pregnant with NC pregnancies. |
| **Selection bias** | | |
| Selection of participants | | The study was performed at Ospedale Sant’Anna, a tertiary-care hospital, in Torino, Italy; the low-risk controls were obtained from the archives of the Torino Cagliari Observational Study, dedicated to women with chronic kidney disease (period 2009-2016). Sant’Anna Hospital is one of the largest European tertiary care obstetric facilities. The selection of the cases of pregnancy in women undergoing OD included all patients who delivered at the Sant’Anna Hospital between January 2008 and February 2019. |
| Loss of follow-up or missing data | | No loss of follow-up or missing data. |

| **Fox, *et al.* 2014 (overlapping cohort with Sekhon *et al.* 2014)** | | |
| --- | --- | --- |
| **Bias due to confounding** | | |
| **Confounding factor** | **Adjusted** | **Explanation** |
| Maternal age | Yes | Multivariable regression adjusted for advanced maternal age. |
| Multiple gestation | Yes | Only twin pregnancies were included. |
| Other | Yes | Multivariable regression adjusted for race, parity, obesity, gestational diabetes, chorionicity, conception type, multifetal reduction, spontaneous reduction, BMI. |
| **Information bias** | | |
| **Information of interest** | | **Acquired by** |
| Exposure: mode of conception | | Of all included pregnancies, risk factors were assessed for PE, including OD. OD and non-OD pregnancies were included from the same cohort, but no information is given on fertility treatment. The NC cohort could have contained women pregnant after OD that did not reveal the origin of their conception. |
| Outcome: hypertensive disorders of pregnancy | | Patients with twin pregnancies were categorized in two groups based on presence or absence of PE (case-control study). PE is defined as hypertension (systolic blood pressure level of 140 mm Hg or higher, or diastolic blood pressure level of 90 mm Hg or higher) with proteinuria (300 mg or more in a 24-hour urine collection). Charts were reviewed for the presence or absence of PE prior to delivery. There is no information whether women pregnant after OD were equally treated as women pregnant with autologous pregnancies. |
| **Selection bias** | | |
| Selection of participants | | All patients with twin pregnancies >20 weeks delivered by a single maternal-fetal medicine practice between June 2005 and June 2012 were included. Patients with monochorionic-monoamniotic placentation and chronic hypertension were excluded. |
| Loss of follow-up or missing data | | No loss to follow-up or missing data. |

| **Gekka, *et al.* 2021** | | |
| --- | --- | --- |
| **Bias due to confounding** | | |
| **Confounding factor** | **Adjusted** | **Explanation** |
| Maternal age | Yes | Multivariable analysis adjusted for maternal age. |
| Multiple gestation | Yes | Multifetal pregnancies were excluded. |
| Other | Yes | Multivariable analysis adjusted for type of conception, parity, BMI, gestational diabetes, adenomyosis, birth weight. |
| **Information bias** | | |
| **Information of interest** | | **Acquired by** |
| Exposure: mode of conception | | With regard to OD pregnancies, data on the country or region from where they received an oocyte, the ethnicity and age of the oocyte donors, and the number of transplanted embryos was retrieved. The NC cohort could have contained women pregnant after OD that did not reveal the origin of their conception. |
| Outcome: hypertensive disorders of pregnancy | | PIH is defined as blood pressure ≥140/90 mmHg at least twice, separated by more than 6 hours, and no proteinuria, PE as repeated measurements of ≥140/90 mmHg, with proteinuria ≥0.3 g/day, and eclampsia as generalized seizures in a context of severe PE (repeated measurements ≥160/110 mmHg, with proteinuria ≥3 g/day). Data was retrospectively extracted from hospital records. There is no information whether women pregnant after OD were equally treated as women pregnant with autologous pregnancies. |
| **Selection bias** | | |
| Selection of participants | | All women aged 40 years or above at delivery who delivered at Aiiku Hospital, one of the largest tertiary hospitals specializing in perinatal care in the metropolitan area of Tokyo, from 1 January 2013 to 31 December 2017 were included. Multifetal pregnancies were excluded. For mothers with multiple deliveries in the hospital during the study period, only their first delivery was considered. Three subgroups according to pregnancy type (OD, IVF, NC) were conducted. |
| Loss of follow-up or missing data | | Of all eligible patients, three were excluded who had hypertension before pregnancy, and one patient with no known HDP outcome. |

| **Guilbaud, *et al.* 2017** | | |
| --- | --- | --- |
| **Bias due to confounding** | | |
| **Confounding factor** | **Adjusted** | **Explanation** |
| Maternal age | Yes | Multivariable regression analysis adjusted for maternal age. |
| Multiple gestation | Yes | Only twin pregnancies included. |
| Other | Yes | Multivariable regression analysis adjusted for parity, mother's geographic origin, and chorionicity. |
| **Information bias** | | |
| **Information of interest** | | **Acquired by** |
| Exposure: mode of conception | | The included women were classified into three groups: OD, IVF, and non-IVF twin pregnancies. However, the non-OD cohort could have contained women pregnant after OD that did not reveal the origin of their conception, as there was no access to data related to the IVF procedure, which was performed elsewhere, sometimes abroad. |
| Outcome: hypertensive disorders of pregnancy | | PIH is defined as systolic blood pressure ≥140 mmHg and/or diastolic blood pressure ≥90 mmHg after 20 weeks’ gestation. PE is defined by hypertension associated with proteinuria ≥300 mg/24h. The obstetric medical file of each included woman was reviewed. There is no information whether women pregnant after OD were equally treated as women pregnant with IVF and non-IVF pregnancies. There is no information whether women pregnant after OD were equally treated as women pregnant with autologous pregnancies. |
| **Selection bias** | | |
| Selection of participants | | This single-center retrospective study took place at a French tertiary university maternal-fetal medicine center with 5,200 births per year. It included all women with twin pregnancies who gave birth after 24 weeks of gestation from January 1, 2010, to October 31, 2014. Women  transferred during pregnancy from another maternity unit because of maternal disease or fetal disease and women with monochorionic monoamniotic pregnancies were excluded. |
| Loss of follow-up or missing data | | No loss of follow-up or missing data. |

| **Hayashi, *et al.* 2019 (conference abstract)** | | |
| --- | --- | --- |
| **Bias due to confounding** | | |
| **Confounding factor** | **Adjusted** | **Explanation** |
| Maternal age | Roughly | Only women over 40 years old were included. However, the abstract does not state anything about the correction for confounders. |
| Multiple gestation | No |  |
| Other | No |  |
| **Information bias** | | |
| **Information of interest** | | **Acquired by** |
| Exposure: mode of conception | | All women of the OD group underwent the embryo transfer at oversea countries. The non-OD cohorts could have contained women pregnant after OD that did not reveal the origin of their conception. |
| Outcome: hypertensive disorders of pregnancy | | No definition of the outcome HDP stated. Retrospective data investigation. There is no information whether women pregnant after OD were equally treated as women pregnant with autologous pregnancies. |
| **Selection bias** | | |
| Selection of participants | | Women, who were over 40 years-old were enrolled and divided into three groups: NC, ART, OD. No information on period or center of inclusion is given in the abstract. |
| Loss of follow-up or missing data | | No loss of follow-up or missing data. |

| **Henne, *et al.* 2007** | | |
| --- | --- | --- |
| **Bias due to confounding** | | |
| **Confounding factor** | **Adjusted** | **Explanation** |
| Maternal age | Yes | Multivariate logistic regression was adjusted for maternal age. |
| Multiple gestation | Yes | Multivariate logistic regression was adjusted for multiple gestation. |
| Other | Yes | Multivariate logistic regression was adjusted for embryonic status (fresh or cryopreserved) and parity. |
| **Information bias** | | |
| **Information of interest** | | **Acquired by** |
| Exposure: mode of conception | | The OD pregnancies were conceived at any fertility center, but no information was given on OD treatment and mode of conception of the autologous pregnancies. The non-exposed cohort could have contained women pregnant after OD that did not reveal the origin of their conception. |
| Outcome: hypertensive disorders of pregnancy | | No definition on PE and HELLP was stated. The obstetric charts of all inclusions were examined. There is no information whether women pregnant after OD were equally treated as women pregnant with non-donor pregnancies. |
| **Selection bias** | | |
| Selection of participants | | All OD pregnancies that delivered between October 1 1997 and May 31 2002 at Lucille Packard Children’s Hospital, Stanford, California were identified. The control group were autologous pregnancies regardless of mode of conception of women aged >38 yrs in the same centre and time period. |
| Loss of follow-up or missing data | | No loss of follow-up or missing data. |

| **Jackson, *et al.* 2015** | | |
| --- | --- | --- |
| **Bias due to confounding** | | |
| **Confounding factor** | **Adjusted** | **Explanation** |
| Maternal age | Roughly | Though only women ≥45 years old were included, the ART group differed significantly from the NC group with regard to maternal age. |
| Multiple gestation | Yes | Only singletons included. |
| Other | No | No adjustment for confounders. Only first pregnancies were included. |
| **Information bias** | | |
| **Information of interest** | | **Acquired by** |
| Exposure: mode of conception | | Prenatal records and chart audits were thoroughly reviewed, as ART information regarding mode of conception is routinely documented in the patient's medical record. NC pregnancy was specified or assumed if a specific ART method was not documented. Therefore, the NC cohort could have contained women pregnant after OD that did not reveal the origin of their conception. |
| Outcome: hypertensive disorders of pregnancy | | No definition on PIH or PE is stated. Data was identified from a department electronic database. There is no information whether women pregnant after OD were equally treated as women pregnant with IVF and non-IVF pregnancies. |
| **Selection bias** | | |
| Selection of participants | | All women ≥45 years old, who delivered at Cedars-Sinai Medical Center between January 2000 and October 2010 were included. The included women were classified into an ART, split in autologous and OD, and a NC group. Twins and higher order  gestations were excluded. |
| Loss of follow-up or missing data | | No loss of follow-up or missing data. |

| **Jeve, *et al.* 2016** | | |
| --- | --- | --- |
| **Bias due to confounding** | | |
| **Confounding factor** | **Adjusted** | **Explanation** |
| Maternal age | Yes | Age-matched control group. Multivariable regression analysis adjusted for maternal age. |
| Multiple gestation | Yes | Multivariable regression analysis adjusted for multiple pregnancy. |
| Other | Yes | Multivariable regression analysis adjusted for BMI, smoking status, gestational age, parity and chronic diseases. |
| **Information bias** | | |
| **Information of interest** | | **Acquired by** |
| Exposure: mode of conception | | For every woman details of assisted conception was collected at booking visit. The control pregnancies were recorded immediately after OD pregnancies in the maternity electronic database. However, the non-exposed cohort could have contained women pregnant after OD that did not reveal the origin of their conception. |
| Outcome: hypertensive disorders of pregnancy | | PIH is defined as blood pressure >140/90 mmHg on two or more occasions at least 6 hours apart, without proteinuria, and presenting after 20 weeks of pregnancy. PE is defined as blood pressure >140/90 mm Hg on two or more occasions at least 6 hours apart, with proteinuria of ≥0.3 g/day, after 20 weeks of pregnancy. Data were collected from booking, prenatal, and intrapartum records, and the maternity electronic database system. All women in the study group were seen in a dedicated prenatal clinic. |
| **Selection bias** | | |
| Selection of participants | | All OD pregnancies with delivery of a live neonate after 24 weeks between January 1, 2007 and December 31, 2014 at a teaching hospital in Leicester, UK, were identified. The control groups were autologous IVF/ICSI pregnancies and NC pregnancies from the same center and period. Pregnancies after preimplantation genetic diagnosis, or after surgical sperm retrieval or use of donor sperm were not included in the study. |
| Loss of follow-up or missing data | | No loss of follow-up or missing data. |

| **Keegan, *et al.* 2007** | | |
| --- | --- | --- |
| **Bias due to confounding** | | |
| **Confounding factor** | **Adjusted** | **Explanation** |
| Maternal age | No | Though there was exclusion of patients aged between 35 and 39 years, this does not imply correction for maternal age. |
| Multiple gestation | No | No multivariable analysis. |
| Other | No | No multivariable analysis. |
| **Information bias** | | |
| **Information of interest** | | **Acquired by** |
| Exposure: mode of conception | | OD and autologous IVF procedures were executed at the center of inclusion. Therefore, the mode of conception is reliable. |
| Outcome: hypertensive disorders of pregnancy | | PIH is defined as high blood pressure during pregnancy for the participants. Obstetric data was collected through questionnaires mailed to the patients. There is no information whether women pregnant after OD were equally treated as women pregnant with non-donor pregnancies. |
| **Selection bias** | | |
| Selection of participants | | Women aged <35 yrs or ≥40 years, who underwent OD or autologous IVF procedures at a university based fertility program that resulted in a fresh embryo transfer with live birth outcome between 1999 and 2003. Cycles resulting in triplet pregnancies, of frozen embryo transfer cycles, or cycles monitored at Program satellite offices were not included. Also excluded were cycles in which the patient age was between 35 and 39 years. |
| Loss of follow-up or missing data | | There was a significant number of loss to follow up (questionnaire response rate was 60%) that could introduce bias. |

| **Kennedy, *et al.* 2019** | | |
| --- | --- | --- |
| **Bias due to confounding** | | |
| **Confounding factor** | **Adjusted** | **Explanation** |
| Maternal age | Yes | Multivariable regression analysis adjusted for maternal age. |
| Multiple gestation | Yes | Only singletons included. |
| Other | Yes | Multivariable regression analysis adjusted for BMI, fertilization via ICSI, and parity. |
| **Information bias** | | |
| **Information of interest** | | **Acquired by** |
| Exposure: mode of conception | | Participating IVF clinics provided de-identified datasets of all IVF-conceived pregnancies, which were divided in donor sperm, OD, embryo donation, and autologous IVF groups. |
| Outcome: hypertensive disorders of pregnancy | | PIH is defined as high blood pressure after 20 weeks of gestation in the absence of other pathology or a diagnosis of PE. PE is defined as high blood pressure and evidence of multisystem dysfunction after 20 weeks of gestations. Data was extracted from a clinical database. There is no information whether women pregnant after donor IVF were equally treated as women pregnant with autologous IVF pregnancies. |
| **Selection bias** | | |
| Selection of participants | | All singleton pregnancies delivering after 20 weeks’ gestation that resulted from IVF donor cycles, across 20 clinics from two large IVF providers in Australia. The control group is defined as autologous IVF cycles resulting in a singleton pregnancy delivering after 20 weeks’ gestation during the same time period at the same IVF sites. Pregnancies resulting in miscarriage, delivery before 20 weeks and multiple pregnancies were excluded. |
| Loss of follow-up or missing data | | There was loss of follow-up of 31 women, unlikely to introduce bias. |

| **Klatsky, *et al.* 2010** | | |
| --- | --- | --- |
| **Bias due to confounding** | | |
| **Confounding factor** | **Adjusted** | **Explanation** |
| Maternal age | Yes | Age-matched control group. Multivariable logistic regression analysis adjusted for age. |
| Multiple gestation | Yes | Plurality-matched control group. Multivariable logistic regression analysis adjusted for multiple gestation. |
| Other | Yes | Multivariable logistic regression analysis adjusted for embryonic origin, and parity. |
| **Information bias** | | |
| **Information of interest** | | **Acquired by** |
| Exposure: mode of conception | | The OD and IVF inclusions were randomly selected from an IVF database, assuring the mode of conception. Though the medical centre of conception was known, no information is stated on the centre of delivery for both groups. |
| Outcome: hypertensive disorders of pregnancy | | PIH is defined as two elevated blood  pressures >140 mmHg systolic or >90 mmHg diastolic, separated by 6 hours. PE was diagnosed as PIH with proteinuria (>300 mg/24h or ≥1+ protein on a catheter-collected urine specimen). Obstetricians of each participant were contacted and asked to provide data or allow access to their records to report specifically on whether the pregnancy was complicated by PIH or PE. There is no information whether women pregnant after OD were equally treated as women pregnant with non- donor pregnancies. |
| **Selection bias** | | |
| Selection of participants | | The OD study population was selected from both singleton and twin pregnancies, that resulted in live births between 1998 and 2005. Women in the study were selected at random from an IVF database maintained at the Weill Cornell medical school in New York, New York, and were matched one-to-one on the basis of age and plurality to controls who delivered after IVF using autologous oocytes during the same year. Women were excluded if they had monozygotic twins or if outcome data were not reported by the obstetrical provider. |
| Loss of follow-up or missing data | | There was a response rate of 79%. |

| **Korb, *et al.* 2020** | | |
| --- | --- | --- |
| **Bias due to confounding** | | |
| **Confounding factor** | **Adjusted** | **Explanation** |
| Maternal age | Yes | The SAMM (severe acute maternal morbidity) and severe hypertensive complications analyses were adjusted for maternal age. |
| Multiple gestation | Yes | Only twin pregnancies were included. |
| Other | Yes | The SAMM (severe acute maternal morbidity) analysis was adjusted for occupation, geographic region of maternal birth, body mass index before pregnancy, parity and previous caesarean, pre-existing chronic condition. |
| **Information bias** | | |
| **Information of interest** | | **Acquired by** |
| Exposure: mode of conception | | The twin pregnancies were classified by use of IVF treatment or not, and IVF conception was additionally categorized by oocyte source (autologous or donor) and ICSI or not. However, the NC cohort could have contained women pregnant after OD that did not reveal the origin of their conception. |
| Outcome: hypertensive disorders of pregnancy | | PE is defined as hypertension ≥140/90 and proteinuria ≥0.3 g/24h, but only if it induced  preterm delivery for a main maternal indication before 32 gestational weeks. Research nurses collected data about maternal characteristics, medical history, pregnancy complications, maternal complications, neonatal health and maternity unit characteristics. Immediately after delivery, obstetricians completed a detailed web-based questionnaire about delivery. There is no information whether women pregnant after medically assisted reproduction were equally treated as women pregnant with NC pregnancies. |
| **Selection bias** | | |
| Selection of participants | | A NC, non-IVF, IVF, ICSI, and OD cohort were collected through the prospective cohort study of twin pregnancies JUmeaux Mode d’Accouchement (JUMODA) which took place in France from 10 February 2014 through 1 March 2015. All French maternity units performing more than 1500 annual deliveries were invited to participate, regardless of their academic, public or private status or level of care, and 176 of the 191 eligible units (92%) did so. Women whose mode of conception was unknown were excluded. |
| Loss of follow-up or missing data | | 75 of 8823 pregnancies were excluded, because of unknown mode of conception, unlikely to introduce bias. |
| **Koren, *et al.* 2021 (conference abstract)** | | |
| **Bias due to confounding** | | |
| **Confounding factor** | **Adjusted** | **Explanation** |
| Maternal age | No | A multivariable logistic regression analysis adjusted for maternal age is not stated in the conference abstract for the outcome PIH. |
| Multiple gestation | Yes | Data were analyzed separately for singletons, twins, and triplets. |
| Other | No |  |
| **Information bias** | | |
| **Information of interest** | | **Acquired by** |
| Exposure: mode of conception | | The pregnancies were divided into three groups according to mode of conception: OD, IVF, and NC. The non-OD cohorts could have contained women pregnant after OD that did not reveal the origin of their conception. |
| Outcome: hypertensive disorders of pregnancy | | No definition for PIH stated in the conference abstract. Data was extracted from a computerized database. There is no information whether women pregnant after OD were equally treated as women pregnant with IVF and NC pregnancies. |
| **Selection bias** | | |
| Selection of participants | | Pregnancies from a retrospective big data cohort based on computerized data of Maccabi healthcare services and conducted from 2000 through 2018 were included. |
| Loss of follow-up or missing data | | No loss of follow-up or missing data. |

| **Krieg, *et al.* 2008** | | |
| --- | --- | --- |
| **Bias due to confounding** | | |
| **Confounding factor** | **Adjusted** | **Explanation** |
| Maternal age | No | Only controls >38 yrs were included. However, the maternal age was still significantly different between the groups. No adjustment for maternal age in multivariable logistic regression. |
| Multiple gestation | Yes | Multivariable logistic regression adjusted for multiple gestation. |
| Other | No |  |
| **Information bias** | | |
| **Information of interest** | | **Acquired by** |
| Exposure: mode of conception | | All ART data were retrieved from Standford Reproductive Endocrinology and Infertility Center, assuring the mode of conception. |
| Outcome: hypertensive disorders of pregnancy | | No definition of PE is stated. Data were retrieved from obstetric records. There is no information whether women pregnant after OD were equally treated as women pregnant with non- donor pregnancies. |
| **Selection bias** | | |
| Selection of participants | | All OD pregnancies that conceived between 2001-2005 at the Stanford Reproductive Endocrinology and Infertility Centre and delivered at Lucille Packard Children’s Hospital were identified. The control group were autologous IVF pregnancies in women >38 yrs that conceived and delivered at the same centres and time period. |
| Loss of follow-up or missing data | | No loss of follow-up or missing data. |

| **Le Ray, *et al.* 2012** | | |
| --- | --- | --- |
| **Bias due to confounding** | | |
| **Confounding factor** | **Adjusted** | **Explanation** |
| Maternal age | Roughly | Only women >43 years were included. |
| Multiple gestation | Yes | Multivariable logistic regression was adjusted for type of pregnancy (singleton or twin). |
| Other | Yes | Multivariable logistic regression was adjusted for parity and mode of conception. |
| **Information bias** | | |
| **Information of interest** | | **Acquired by** |
| Exposure: mode of conception | | The included women were classified into three groups: no IVF, IVF without OD, and OD. Dual donations (sperm and oocytes) were also recorded. Because of the potential information bias due to underreporting in the medical files, NC pregnancies were combined with those obtained without IVF but with other ART techniques. However, the NC cohort could have contained women pregnant after OD that did not reveal the origin of their conception, as there was no access to data related to the IVF procedure, which was performed elsewhere, sometimes abroad. |
| Outcome: hypertensive disorders of pregnancy | | PE is defined by blood pressure ≥140/90 mmHg and proteinuria ≥0.3 g/24h. Data were collected from obstetric records, and all records of such pregnancies were individually reviewed. There is no information whether women pregnant after OD were equally treated as women pregnant with non-donor pregnancies. |
| **Selection bias** | | |
| Selection of participants | | This study took place at Port Royal Maternity Hospital in Paris (France), a level-III university hospital center that handles around 3200 deliveries per year. It included all women who gave birth after their 43^rd^ birthday from 1 January 2008 through 31 December 2010. |
| Loss of follow-up or missing data | | No loss of follow-up or missing data. |

| **Letur, *et al.* 2016** | | |
| --- | --- | --- |
| **Bias due to confounding** | | |
| **Confounding factor** | **Adjusted** | **Explanation** |
| Maternal age | Yes | Controls were matched for maternal age. Multivariable logistic regression analysis adjusted for maternal age. |
| Multiple gestation | Yes | Only singleton pregnancies were included. |
| Other | Yes | Controls were matched for parity, embryo transfer date and transfer technique. Multivariable logistic regression analysis adjusted for body mass index, previous pregnancy, cycle rank, number of transferred embryos, pregnancy origin (frozen embryo replacements or not). |
| **Information bias** | | |
| **Information of interest** | | **Acquired by** |
| Exposure: mode of conception | | All ART data were retrieved from the participating fertility centers, assuring mode of conception. This study did not include pregnancies originating from OD performed abroad, because most of them were not registered in a French ART center and the risk of bias could be high. |
| Outcome: hypertensive disorders of pregnancy | | PIH occurring after 20 weeks of gestation is defined as blood pressure ≥140/90 mmHg at least twice, separated by more than 6 hours, and no proteinuria. PE is defined as repeated ≥140/90 mmHg, with proteinuria ≥0.3 g/day. Eclampsia is defined as generalized seizures in a context of severe PE (repeated ≥160/110 mmHg, with proteinuria ≥3 g/day). Each center's coordinator consulted each woman's medical chart to collect data. This information was then entered anonymously on a designated form and transmitted to the study group. There is no information whether women pregnant after OD were equally treated as women pregnant with non-donor pregnancies. |
| **Selection bias** | | |
| Selection of participants | | All singleton OD pregnancies between February 2005 and September 2012 from seven French centres. The control groups were singleton autologous IVF/ICSI pregnancies achieved at the same center. For each OD pregnancy the first two subsequent IVF/ICSI pregnancies were obtained as a control. Multiple pregnancies and OD treatment performed abroad were excluded. |
| Loss of follow-up or missing data | | There was a small number of loss to follow up, unlikely to introduce bias. |

| **Levron, *et al.* 2014** | | |
| --- | --- | --- |
| **Bias due to confounding** | | |
| **Confounding factor** | **Adjusted** | **Explanation** |
| Maternal age | Yes | Multivariable logistic regression analysis adjusted for maternal age, gravidity, parity and presence of chronic hypertension. |
| Multiple gestation | Yes | Only singleton pregnancies included. |
| Other | Yes | Multivariable logistic regression analysis adjusted for gravidity, parity and presence of chronic hypertension. |
| **Information bias** | | |
| **Information of interest** | | **Acquired by** |
| Exposure: mode of conception | | The medical files of prenatal and delivery care of all inclusions were examined on mode of conception. However, the non-OD group could have contained women pregnant after OD that did not reveal the origin of their conception, as there was information on data related to the IVF procedure given. |
| Outcome: hypertensive disorders of pregnancy | | PIH is defined as blood pressure ≥140/90 mm Hg measured on 2 occasions at least 4 hours apart, occurring after 20 weeks of gestation in a previously normotensive woman. PE was diagnosed when PIH was accompanied by proteinuria (≥300 mg/24 hours or 2+ dipstick). HDP is defined as the presence of PIH or PE. Pregnancy outcomes were abstracted from obstetric electronic charts. There is no information whether women pregnant after OD were equally treated as women pregnant with non-donor pregnancies. |
| **Selection bias** | | |
| Selection of participants | | All women who had conceived through OD and received prenatal care and delivered at a single tertiary medical center between 2005 and 2011. The control groups were matched IVF pregnancies in women >38 years in the same centre and time period. Multiple pregnancies were excluded. |
| Loss of follow-up or missing data | | No loss of follow-up or missing data. |

| **Luke, *et al.* 2020** | | |
| --- | --- | --- |
| **Bias due to confounding** | | |
| **Confounding factor** | **Adjusted** | **Explanation** |
| Maternal age | Yes | Multivariable logistic regression adjusted for maternal age. |
| Multiple gestation | Yes | Only singleton pregnancies included. |
| Other | Yes | Multivariable logistic regression adjusted for fertility group, race and ethnicity, education, parity, State of residence, year of birth, and infant sex, overall and by early-onset (<34 weeks) hypertensive disorders of pregnancy, and by late-onset (≥34 weeks). |
| **Information bias** | | |
| **Information of interest** | | **Acquired by** |
| Exposure: mode of conception | | The IVF pregnancies were categorized by oocyte source (autologous vs donor) and embryo state (fresh vs thawed) using data from a national IVF database. However, the NC cohort could have contained women pregnant after OD that did not reveal the origin of their conception. |
| Outcome: hypertensive disorders of pregnancy | | PIH is defined per ACOG guidelines as blood pressure ≥140 mmHg systolic or ≥90 mmHg diastolic on two separate occasions at least four hours apart after 20 weeks of pregnancy when previous blood pressure was normal. PE is defined as PIH with ≥300mg urine protein excretion in a 24-hour period or a protein/creatinine ratio of greater than or equal to 0.3 or urine protein dipstick 1+. The data were extracted from the birth certificates. There is no information whether women pregnant after OD were equally treated as women pregnant with IVF and NC pregnancies. |
| **Selection bias** | | |
| Selection of participants | | Women in 8 States who underwent IVF resulting in a live birth during 2004 through 2013 were linked to their infant’s birth certificates. A 10:1 sample of births from non-IVF deliveries were selected for comparison. |
| Loss of follow-up or missing data | | No loss of follow-up or missing data. |

| **Malchau, *et al.* 2013** | | |
| --- | --- | --- |
| **Bias due to confounding** | | |
| **Confounding factor** | **Adjusted** | **Explanation** |
| Maternal age | Yes | Multivariable logistic regression analyses adjusted for maternal age. |
| Multiple gestation | Yes | Subroup analyses for singletons and twins. |
| Other | Yes | Multivariable logistic regression analyses adjusted for parity, child sex and year of birth. |
| **Information bias** | | |
| **Information of interest** | | **Acquired by** |
| Exposure: mode of conception | | All women pregnant after OD or IVF/ICSI were identified from the IVF register. However, the NC cohort could have contained women pregnant after OD that did not reveal the origin of their conception. Also, it is unclear whether the NC group was also matched for region of Denmark. |
| Outcome: hypertensive disorders of pregnancy | | HDP is defined as chronic hypertension, PIH, and PE. No definition on PIH and PE was given. Data on perinatal outcomes were extracted from the Medical Birth Register and Hospital Discharge Register. There is no information whether women pregnant after OD were equally treated as women pregnant with non-donor pregnancies. |
| **Selection bias** | | |
| Selection of participants | | All OD pregnancies between 1995 and 2010 from the Danish IVF register were identified. The control groups were IVF/ICSI pregnancies and NC pregnancies. This latter control group was matched by date and year of birth. Women who had an OD treatment abroad were excluded. |
| Loss of follow-up or missing data | | No information is given about loss of follow up. The total number of OD women with an outcome is stated in tables, but this number is lower than the total number of OD women included. |

| **Masturzo , *et al.* 2019 ((overlapping cohort with Fassio *et al.* 2019)** | | |
| --- | --- | --- |
| **Bias due to confounding** | | |
| **Confounding factor** | **Adjusted** | **Explanation** |
| Maternal age | Yes | Binary logistic regression was used to calculate the rate of early-onset PE stratified according to maternal age. |
| Multiple gestation | Yes | Binary logistic regression was stratified according to number of fetuses (one or two). |
| Other | Yes | Binary logistic regression was adjusted for BMI. |
| **Information bias** | | |
| **Information of interest** | | **Acquired by** |
| Exposure: mode of conception | | All procedures of OD had been performed abroad, because the Italian law did not allow ART with donated gametes at the time of the study development. Therefore, the NC cohort could have contained women pregnant after OD that did not reveal the origin of their conception. |
| Outcome: hypertensive disorders of pregnancy | | PE is defined as systolic blood pressure ≥140 mmHg and/or diastolic blood pressure ≥90 mmHg occurred on at least two occasions 4h apart, developed after 20 weeks of gestation in previously normotensive women. There should also be proteinuria ≥ 300mg in 24h or two readings of at least ++ on dipstick analysis of midstream or catheter urine specimens if no 24h collection was available. All data were obtained from the hospital records. There is no information whether women pregnant after OD were equally treated as women pregnant with non-donor pregnancies. |
| **Selection bias** | | |
| Selection of participants | | Women pregnant through OD, who received prenatal care until delivery at the Departments of Obstetrics and Gynecology of 3 tertiary Referral Hospitals located in Northern Italy (Sant’Anna  University Hospital in Turin, Buzzi Hospital in Milan  and Spedali Civili in Brescia) between January 2008 and December 2017. The control group consisted of NC pregnancies examined at the same hospitals with the same criteria for in- and exclusion. |
| Loss of follow-up or missing data | | No loss of follow-up or missing data. |

| **Meyer, *et al.* 2020** | | |
| --- | --- | --- |
| **Bias due to confounding** | | |
| **Confounding factor** | **Adjusted** | **Explanation** |
| Maternal age | Roughly | In order to compare groups of similar ages, the analysis was restricted to women aged 45–47 years at delivery. However, maternal age was still significantly different between the study groups. |
| Multiple gestation | Yes | Only singleton pregnancies included. |
| Other | Yes | Multivariable logistic regression analysis adjusted for term BMI, chronic hypertension, and use of low molecular weight heparin during pregnancy. |
| **Information bias** | | |
| **Information of interest** | | **Acquired by** |
| Exposure: mode of conception | | Mode of conception was acquired from the study hospital’s computerized medical database. However, the NC cohort could have contained women pregnant after OD that did not reveal the origin of their conception. |
| Outcome: hypertensive disorders of pregnancy | | PIH is defined per ACOG guidelines as blood pressure ≥140 mmHg systolic or ≥90 mmHg diastolic on two separate occasions at least four hours apart after 20 weeks of pregnancy when previous blood pressure was normal. PE is defined as PIH with ≥300mg urine protein excretion in a 24-hour period or a protein/creatinine ratio of greater than or equal to 0.3 or urine protein dipstick 1+. Data were collected from delivery room medical records. All women of 44 years or older were followed in the high-risk clinic and treated with low dose aspirin. However, most women in the cohort had their pregnancy follow-up outside of the study hospital, in which case the decision to treat or not to treat with aspirin was at the discretion of the treating physician. |
| **Selection bias** | | |
| Selection of participants | | All women aged 45–47 years at time of delivery, who gave birth to singletons between 21 March 2011 and 2 May 2018 at a tertiary university-affiliated medical centre were included. Only women who gave birth at 24 weeks’ gestation or later were included, stratified by the mode of conception: OD, IVF and NC. Cases in which the mode of conception was not clearly described in the medical records, multiple gestation pregnancies, pregnancies following ovulation induction, and pregnancies with fetal reduction were excluded. |
| Loss of follow-up or missing data | | Eighteen excluded cases are clearly stated with reason of exclusion. |
| **Modest, *et al.* 2019** | | |
| **Bias due to confounding** | | |
| **Confounding factor** | **Adjusted** | **Explanation** |
| Maternal age | Yes | Multivariable logistic regression analysis was adjusted for maternal age. |
| Multiple gestation | Yes | Sensitivity analysis restricted to singleton pregnancies. |
| Other | Yes | Multivariable logistic regression analysis was adjusted for parity, and marital status. |
| **Information bias** | | |
| **Information of interest** | | **Acquired by** |
| Exposure: mode of conception | | Oocyte source was abstracted through electronic medical records at Boston IVF, the study hospital’s affiliated infertility treatment center, or through the birth certificate data from the Massachusetts Department of Public Health. Pregnancies not identified as a result of donor IVF were considered to be from autologous IVF. Deliveries that were not identified as resulting from IVF by the Boston IVF data or birth certificate data were considered non-IVF pregnancies. Therefore, the IVF and non-IVF cohort could have contained women pregnant after OD that did not reveal the origin of their conception or did not have the right documentation. |
| Outcome: hypertensive disorders of pregnancy | | PE is defined as the presence of elevated blood pressure (≥140/90) during the delivery admission, and either symptoms of PE (headache, visual changes, severe right upper abdominal pain), seizures, or abnormal laboratory values (proteinuria, alanine aminotransferase/aspartate aminotransferase ≥ 80 units per liter, or platelets <100,000) before delivery. Demographic and outcome data were either self-reported during hospital registration or recorded by a clinician at delivery. There is no information whether women pregnant after OD were equally treated as women pregnant with non-donor pregnancies. |
| **Selection bias** | | |
| Selection of participants | | All deliveries of live-born infants and intrauterine fetal demise at or after 20 weeks of gestation from January 1, 2000, to June 1, 2015, at Beth Israel Deaconess Medical Center, a large tertiary care hospital. Pregnancies of mothers less than 18 years of age, and IVF cycles that ended in an ectopic pregnancy, miscarriage, or induced abortion were excluded. Three exposure groups were evaluated: OD, IVF, non-IVF. |
| Loss of follow-up or missing data | | No loss of follow-up or missing data. |
| **Nejdet, *et al.* 2016** | | |
| **Bias due to confounding** | | |
| **Confounding factor** | **Adjusted** | **Explanation** |
| Maternal age | Yes | Multivariable logistic regression analysis adjusted for maternal age, year of birth, parity, smoking, BMI, years of subfertility, IVF/ICSI, fresh/ frozen embryos and number of embryos transferred. |
| Multiple gestation | Yes | Only singleton pregnancies included. |
| Other | Yes | Multivariable logistic regression analysis adjusted for year of birth, parity, smoking, BMI, years of subfertility, IVF/ICSI, fresh/frozen embryos, and number of embryos transferred. |
| **Information bias** | | |
| **Information of interest** | | **Acquired by** |
| Exposure: mode of conception | | All ART pregnancies were identified through data from IVF clinics, assuring the mode of conception. However, the NC cohort could have contained women pregnant after OD that did not reveal the origin of their conception. |
| Outcome: hypertensive disorders of pregnancy | | PE and eclampsia were defined according to the International Classification of Diseases 10 codes O.14 and O.15. Outcome data was collected through the Swedish Medical Birth Registry. There is no information whether women pregnant after OD were equally treated as women pregnant with non-donor pregnancies. |
| **Selection bias** | | |
| Selection of participants | | All singleton OD pregnancies between 2003 and 2012 from Swedish IVF clinics were identified. The control groups were IVF/ICSI pregnancies and NC pregnancies. The latter group was identified during the same time period from the Swedish Medical Birth Registry. |
| Loss of follow-up or missing data | | No loss of follow-up or missing data. |

| **Rizzello, *et al.* 2020** | | |
| --- | --- | --- |
| **Bias due to confounding** | | |
| **Confounding factor** | **Adjusted** | **Explanation** |
| Maternal age | Yes | Multivariable logistic regression analysis adjusted for maternal age. |
| Multiple gestation | Yes | Only singleton pregnancies included. |
| Other | Yes | Multivariable logistic regression analysis adjusted for maternal BMI, parity and maternal smoking. |
| **Information bias** | | |
| **Information of interest** | | **Acquired by** |
| Exposure: mode of conception | | No information is given on the origin of the ART data. The NC cohort could have contained women pregnant after OD that did not reveal the origin of their conception. |
| Outcome: hypertensive disorders of pregnancy | | HDP included chronic hypertension and *de novo* hypertension, either PIH or PE. PIH is defined as blood pressure that is ≥140 mmHg systolic and/or 90 mmHg diastolic after 20 weeks of gestation. PE was diagnosed by the presence of de novo hypertension after 20 weeks’ gestation accompanied by proteinuria and/or evidence of maternal acute kidney injury, liver dysfunction, neurological features, hemolysis or thrombo-cytopenia, or fetal growth restriction. Clinical information for all women was obtained from an electronic medical records database. There is no information whether women pregnant after OD were equally treated as women pregnant with non-donor pregnancies. |
| **Selection bias** | | |
| Selection of participants | | Women who conceived through OD, IVF/ICSI or NC, and giving birth at the University Hospital of  Careggi, Florence, from January 2009 to May 2017. |
| Loss of follow-up or missing data | | No loss of follow-up or missing data. |

| **Rizzo, *et al.* 2016** | | |
| --- | --- | --- |
| **Bias due to confounding** | | |
| **Confounding factor** | **Adjusted** | **Explanation** |
| Maternal age | Yes | Multivariable logistic regression analysis adjusted for maternal age. |
| Multiple gestation | Yes | Only singleton pregnancies included. |
| Other | Yes | Multivariable logistic regression analysis adjusted for placental volume, and mean UtA-PI MoM. |
| **Information bias** | | |
| **Information of interest** | | **Acquired by** |
| Exposure: mode of conception | | No information is given on the origin of the ART data. The NC cohort could have contained women pregnant after OD that did not reveal the origin of their conception. |
| Outcome: hypertensive disorders of pregnancy | | PE is defined according to the guidelines of the International Society for the Study of Hypertension in Pregnancy (2001). Data was extracted from medical records. There is no information whether women pregnant after OD were equally treated as women pregnant with non-donor pregnancies. |
| **Selection bias** | | |
| Selection of participants | | Pregnant women in this study were recruited from those attending ultrasound screening at 11+0 to 13+6 weeks’ gestation, from January 2007 to January 2014, as part of a prospective project on placental development. Consecutive nulliparous singleton pregnancies achieved by IVF with autologous or donor oocytes were considered. Further criteria of inclusion were: successful ultrasound recordings of placental volume and UtA Doppler velocity waveforms, absence of pre-existing maternal diseases, absence of fetal structural or chromosomal anomalies, and exhaustive follow-up. As a control group, nulliparous singleton pregnancies attending the same antenatal clinic during the same study period that fulfilled the above-reported criteria were selected. |
| Loss of follow-up or missing data | | Six pregnancies were lost to follow-up and therefore excluded, unlikely to introduce bias. |

| **Rodriguez-Wallberg, *et al.* 2019** | | |
| --- | --- | --- |
| **Bias due to confounding** | | |
| **Confounding factor** | **Adjusted** | **Explanation** |
| Maternal age | Yes | Age-matched control group. |
| Multiple gestation | Yes | Only singleton pregnancies included. |
| Other | Yes | Multivariable logistic regression analysis adjusted for BMI, smoking, and parity. Matching on type of treatment (IVF/ICSI), and year of embryo transfer. |
| **Information bias** | | |
| **Information of interest** | | **Acquired by** |
| Exposure: mode of conception | | All ART treatments were performed at the Reproductive Medicine Unit, Karolinska University Hospital, assuring the mode of conception. Data on IVF/ICSI treatments using OD or autologous oocytes were obtained from the Reproductive Medicine’s electronic database. |
| Outcome: hypertensive disorders of pregnancy | | Obstetric outcome variables were  extracted through their ICD-10. Delivery data are prospectively entered in a medical registry database with a >95% completeness. There is no information whether women pregnant after OD were equally treated as women pregnant with non-donor pregnancies. However, Swedish maternal healthcare is standardized and also free of charge. |
| **Selection bias** | | |
| Selection of participants | | Women who achieved pregnancies and live births using donor oocytes after IVF with or without ICSI, compared 1:2 to control women undergoing IVF/ICSI with autologous oocytes. All ART treatments were performed at the Reproductive Medicine Unit, Karolinska University Hospital between January 1 2007 and December 31 2014 with a strict policy of single embryo transfer. |
| Loss of follow-up or missing data | | No loss of follow-up or missing data. |

| **Rudenko, *et al.* 2020** | | |
| --- | --- | --- |
| **Bias due to confounding** | | |
| **Confounding factor** | **Adjusted** | **Explanation** |
| Maternal age | No | No multivariable regression analysis. |
| Multiple gestation | No |  |
| Other | No |  |
| **Information bias** | | |
| **Information of interest** | | **Acquired by** |
| Exposure: mode of conception | | No information is given on the origin of the ART data. |
| Outcome: hypertensive disorders of pregnancy | | Primary outcome was structural and immunohistochemical features of placentas, but PIH and PE rates were also documented. Though, nothing is stated about how clinical data was retrieved. |
| **Selection bias** | | |
| Selection of participants | | The main group involved women whose pregnancy occurred as a result of OD in a surrogate motherhood program or OD. The comparison group consisted of autologous IVF patients. Inclusion criteria were singleton pregnancy, emergency or planned delivery  by Cesarean section, or natural birth after 22 weeks of pregnancy. Exclusion criteria were severe extragenital pathologies, as well as pregnancy complications with well-studied pathogenesis. No information on center and period of inclusion. |
| Loss of follow-up or missing data | | No loss of follow-up or missing data. |

| **Salha, *et al.* 1999** | | |
| --- | --- | --- |
| **Bias due to confounding** | | |
| **Confounding factor** | **Adjusted** | **Explanation** |
| Maternal age | Yes | Matched for age. |
| Multiple gestation | No |  |
| Other | Yes | Matched for parity and demographic background. |
| **Information bias** | | |
| **Information of interest** | | **Acquired by** |
| Exposure: mode of conception | | Infertility records were reviewed to divide the study patients into three groups depending on the origin of the donated gametes: IUI with donor sperm, OD, and ED. However, the non-exposed cohort could have contained women pregnant after OD that did not reveal the origin of their conception. |
| Outcome: hypertensive disorders of pregnancy | | PIH is defined as blood pressure ≥140/90 mmHg measured on two or more occasions at least 6 h apart without proteinuria after 20 weeks gestation or a diastolic pressure >20 mm Hg above the booking level. PE is defined as blood pressure ≥140/90 mmHg measured on two or more occasions at least 6 h apart with proteinuria ≥0.5 g/day after 20 weeks gestation. Medical records were reviewed to obtain outcome data. There is no information whether women pregnant after OD were equally treated as women pregnant with non- donor pregnancies. |
| **Selection bias** | | |
| Selection of participants | | Women who conceived with donated gametes and delivered at ≥24 weeks gestation either at St James's University Hospital, Leeds or the Leeds General Infirmary between 1992 and 1997. The control group consisted of patients from the birth register who conceived with their own gametes and delivered at either SJUH or LGI during the study period. |
| Loss of follow-up or missing data | | No loss of follow-up or missing data. |

| **Sekhon, *et al.* 2014 (overlapping cohort with Fox *et al.* 2014)** | | |
| --- | --- | --- |
| **Bias due to confounding** | | |
| **Confounding factor** | **Adjusted** | **Explanation** |
| Maternal age | Yes | The control group was matched by age. |
| Multiple gestation | Yes | Only twin pregnancies were included. |
| Other | Yes | The control group was closely matched by parity, pre-pregnancy BMI, and their obstetric and gynecologic history. |
| **Information bias** | | |
| **Information of interest** | | **Acquired by** |
| Exposure: mode of conception | | Patients who underwent OD and age-matched controls who underwent IVF using autologous oocytes were compared. Controls were obtained by identifying the next patient with an IVF twin delivery whose age was within 2 years of the age of the case patient. The fertility treatment was provided from several ART centers in the New York area, and patients presented for obstetric care in the practice after an intrauterine twin pregnancy had been documented. |
| Outcome: hypertensive disorders of pregnancy | | PIH is defined as a systolic blood pressure level of ≥140 mmHg or a diastolic blood pressure level of ≥90 mmHg any time after 20 weeks. PE is defined as PIH plus proteinuria (≥300 mg in a 24-hour urine collection). The electronic patient database was used to collect detailed information on pregnancy outcomes, for all patients. The management of twin pregnancies did not differ according to whether the patient underwent OD. |
| **Selection bias** | | |
| Selection of participants | | All patients with twin pregnancies >24 weeks delivered by a single maternal–fetal medicine practice between June 2005 and June 2013 were included. Patients with monochorionic-monoamniotic placentation and >50 years of age were excluded. |
| Loss of follow-up or missing data | | No loss to follow-up or missing data. |

| **Serena, *et al.* 2019 (overlapping cohort with Simeone, *et al*. 2016)** | | |
| --- | --- | --- |
| **Bias due to confounding** | | |
| **Confounding factor** | **Adjusted** | **Explanation** |
| Maternal age | Yes | Multivariable logistic regression adjusted for maternal age. |
| Multiple gestation | Yes | Only singleton pregnancies included. |
| Other | No |  |
| **Information bias** | | |
| **Information of interest** | | **Acquired by** |
| Exposure: mode of conception | | No information is given on the retrieval of fertility treatment data. The NC cohort could have contained women pregnant after OD that did not reveal the origin of their conception. |
| Outcome: hypertensive disorders of pregnancy | | PIH is defined as systolic blood pressure ≥140 mmHg and diastolic blood pressure ≥90 mmHg occurring after 20 weeks in previously normotensive women. PE is defined as PIH with proteinuria ≥3 g over 24-hour interval occurring after 20 weeks in previously normotensive women. No information on how outcome data was retrieved, but probably from the hospital records. There is no information whether women pregnant after OD were equally treated as women pregnant with non-donor pregnancies. |
| **Selection bias** | | |
| Selection of participants | | All singleton pregnancies obtained by OD referred at High-Risk Pregnancy Unit in Careggi Hospital, Florence, between January 2011 and August 2017, with a gestational age ≥24 weeks at delivery. The control group included singleton IVF and NC pregnancies that gave birth at the same time. Double gamete donation pregnancies or sperm donation pregnancies, multiple pregnancies and spontaneous abortion <24 weeks were excluded. |
| Loss of follow-up or missing data | | No loss of follow-up or missing data. |

| **Shrim, *et al.* 2010** | | |
| --- | --- | --- |
| **Bias due to confounding** | | |
| **Confounding factor** | **Adjusted** | **Explanation** |
| Maternal age | Yes | Age-matched control group. |
| Multiple gestation | Yes | Only singleton pregnancies included. |
| Other | No |  |
| **Information bias** | | |
| **Information of interest** | | **Acquired by** |
| Exposure: mode of conception | | The control group is not properly defined by mode of conception. Furthermore, the control group could have contained women pregnant after OD that did not reveal the origin of their conception. |
| Outcome: hypertensive disorders of pregnancy | | No definition of PIH is stated. Data was retrieved from a database. There is no information whether women pregnant after OD were equally treated as women pregnant with non-donor pregnancies. |
| **Selection bias** | | |
| Selection of participants | | All singleton pregnancies between the years 2001–2007 were retrieved from the McGill Obstetrics and Neonatal Database. Obstetric and neonatal outcomes were compared between different maternal age groups, with and without OD. Each woman with OD pregnancy was matched with the consecutive three women with singleton pregnancy that gave birth on the same day (or the following day if matched controls were not  found on the same day). Teenage pregnancies were excluded. |
| Loss of follow-up or missing data | | Four pregnancies achieved with OD in which maternal age was 49 years were excluded because no matched controls could be found. |

| **Simchen, *et al.* 2009** | | |
| --- | --- | --- |
| **Bias due to confounding** | | |
| **Confounding factor** | **Adjusted** | **Explanation** |
| Maternal age | No | There was no adjustment for maternal age, though there was a significant difference in maternal age between the twin OD and autologous twin group. However, in the comparison of OD twins and OD singletons, maternal age did not differ between the groups. |
| Multiple gestation | Yes | Comparison of twin OD and autologous twin pregnancies, next to comparison of singleton and twin OD pregnancies. |
| Other | No |  |
| **Information bias** | | |
| **Information of interest** | | **Acquired by** |
| Exposure: mode of conception | | No information is given on the origin of the ART data. Furthermore, the mode of conception of the autologous twin control group is unknown. However, the control twin group could have contained women pregnant after OD that did not reveal the origin of their conception. |
| Outcome: hypertensive disorders of pregnancy | | PIH is defined according to the ACOG guidelines (2002) as systolic blood pressure of ≥140 mmHg or diastolic blood pressure of ≥90 mmHg that occurs after 20 weeks of gestation in a woman with previously normal blood pressure. Data was extracted from a computerized database, entered by the obstetrician or midwife responsible for the laboring woman’s care.There is no information whether women pregnant after OD were equally treated as women pregnant with non-donor pregnancies. |
| **Selection bias** | | |
| Selection of participants | | Women who were at least 40 years of age or older at the time of delivery, who conceived after OD and whose pregnancy continued to delivery between 1999 and 2008 were included. As controls, all women carrying twins who gave birth at Sheba Medical Center between 1 January 2007 and 31 December 2007 were included. |
| Loss of follow-up or missing data | | One twin OD pregnancy was excluded, because of fetal loss of one fetus. |

| **Simeone, *et al.* 2016 (overlapping cohort with Serena, *et al*. 2019)** | | |
| --- | --- | --- |
| **Bias due to confounding** | | |
| **Confounding factor** | **Adjusted** | **Explanation** |
| Maternal age | No | Only including women >35 yrs, no significant difference between the groups. |
| Multiple gestation | Roughly | Subgroup analysis only including singletons. |
| Other | Roughly | Subgroup analysis excluding chronic hypertension. |
| **Information bias** | | |
| **Information of interest** | | **Acquired by** |
| Exposure: mode of conception | | No information is given on the retrieval of fertility treatment data. |
| Outcome: hypertensive disorders of pregnancy | | PE is defined as blood pressure of ≥140 mmHg systolic or ≥90 mm Hg diastolic on at least two occasions and at least 4–6 h apart after the 20th week of gestation in women known to be normotensive beforehand with proteinuria ≥300 mg every 24 h. No information on how outcome data was retrieved, but probably from the hospital records. There is no information whether women pregnant after OD were equally treated as women pregnant with non-donor pregnancies. |
| **Selection bias** | | |
| Selection of participants | | All OD pregnancies between 2009-2011 from women aged >35 yrs in the High Risk Pregnancy-AOU Careggi University Hospital, Italy, were identified. The control groups were IVF/ICSI pregnancies in women aged >35 yrs. |
| Loss of follow-up or missing data | | There was a small number of patients excluded for analysis in the OD group, unlikely to introduce bias. |

| **Sites, *et al.* 2017** | | |
| --- | --- | --- |
| **Bias due to confounding** | | |
| **Confounding factor** | **Adjusted** | **Explanation** |
| Maternal age | Yes | Multivariable logistic regression analysis adjusted for maternal age. |
| Multiple gestation | Yes | Subgroup analyses for singleton and twin pregnancies. |
| Other | Yes | Multivariable logistic regression analysis adjusted for birth year, infant sex, maternal race, diabetes (pregestational or gestational), hypertension (chronic or gestational), and parity. |
| **Information bias** | | |
| **Information of interest** | | **Acquired by** |
| Exposure: mode of conception | | Data from the States Monitoring ART Collaborative were used, assuring the mode of conception. |
| Outcome: hypertensive disorders of pregnancy | | PIH, PE, and eclampsia are defined according to the ICD-9 codes. Linked ART surveillance, birth certificate, and maternal hospitalization discharge data were used. There is no information whether women pregnant after OD were equally treated as women pregnant with non-donor pregnancies. |
| **Selection bias** | | |
| Selection of participants | | Resident singleton and twin live births to women occurring in Massachusetts at >20 weeks of estimated gestational age from 2005 through 2010 were included. Births resulting from fresh embryo transfer were compared with those from cryopreserved-warmed embryo transfer, stratified by use of autologous or donor oocytes. At the time of this analysis, only Massachusetts had linked hospital discharge data; therefore, data from Connecticut and Michigan were excluded. |
| Loss of follow-up or missing data | | No loss of follow-up or missing data. |

| **Söderström-Anttila, *et al.* 1998** | | |
| --- | --- | --- |
| **Bias due to confounding** | | |
| **Confounding factor** | **Adjusted** | **Explanation** |
| Maternal age | No | No adjustment, though, no significant difference in maternal age between the OD and IVF group. |
| Multiple gestation | Yes | Subgroup analysis for singleton pregnancies. |
| Other | Yes | Subgroup analysis for primiparous and multiparous women. |
| **Information bias** | | |
| **Information of interest** | | **Acquired by** |
| Exposure: mode of conception | | ART data was retrieved from the participating fertility clinic, assuring the mode of conception. |
| Outcome: hypertensive disorders of pregnancy | | PIH is defined as blood pressure ≥140/90 mmHg on two or more occasions at least 6 h apart after 20 weeks of gestation. PE is defined as PIH with proteinuria ≥0.5 g/day after 20 weeks of gestation. Data was retrieved from the obstetric notes from the hospital of delivery, and a questionnaire was sent to all patients. Obstetric and intrapartum care in both the OD and IVF group was received at different hospitals with regard to region, and primary, secondary or tertiary care. |
| **Selection bias** | | |
| Selection of participants | | All OD pregnancies that conceived between October 1991 and December 1996 at the Infertility clinic of the family Federation of Finland were identified. Delivery took place in different centres in Finland and Sweden. For every OD patient, two IVF patients who had undergone embryo transfer immediately before and after and who gave birth at ≥24 weeks of gestation were selected. |
| Loss of follow-up or missing data | | All OD patients answered the questionnaire, but of the IVF patients 95% responded, unlikely to introduce bias. |

| **Stoop, *et al.* 2012** | | |
| --- | --- | --- |
| **Bias due to confounding** | | |
| **Confounding factor** | **Adjusted** | **Explanation** |
| Maternal age | Yes | Matching for maternal age. |
| Multiple gestation | Yes | Matching and multivariable logistic regression adjusting for plurality. |
| Other | Yes | Matching for parity and ethnicity. In addition, multivariable logistic regression adjusting for paternal age, age of the oocyte donor, and the number of embryos transferred. |
| **Information bias** | | |
| **Information of interest** | | **Acquired by** |
| Exposure: mode of conception | | Data from all OD and IVF pregnancies were included from the same study cohort of the Centre of Reproductive Medicine, University Hospital Brussels. |
| Outcome: hypertensive disorders of pregnancy | | PIH is defined as blood pressure levels ≥140/90 mmHg on two or more occasions at least 6 h apart, without proteinuria, after 20 weeks. PE is defined as repeated BP levels ≥140/90 mm Hg with proteinuria ≥0.3 g/day after 20 weeks of gestation. For all pregnancies, written data regarding obstetrical and neonatal outcome were collected. A questionnaire was send to all patients with an ongoing pregnancy at seven weeks of gestation and to their obstetrician around the estimated delivery date. Some gynaecologists needed to be contacted by phone in order to retrieve the outcome. There is no information whether women pregnant after OD were equally treated as women pregnant with ART pregnancies. |
| **Selection bias** | | |
| Selection of participants | | All pregnancies that occurred between January 1999 and December 2008, that had  been obtained after OD and resulted in offspring after more than 20 weeks of gestation, were included in this study. Matched controls were selected from the patient population that underwent IVF with autologous oocytes during the same period in the same center. Pregnancies after preimplantation genetic diagnosis or after testicular sperm extraction or use of donor sperm were excluded. |
| Loss of follow-up or missing data | | There was a small number of loss to follow up because of the lack of appropriate matches, unlikely to introduce bias. |

| **Tarlatzi, *et al.* 2016** | | |
| --- | --- | --- |
| **Bias due to confounding** | | |
| **Confounding factor** | **Adjusted** | **Explanation** |
| Maternal age | Yes | Age-matched control group. |
| Multiple gestation | Yes | Only singleton pregnancies included. |
| Other | Yes | Matching for parity and gravidity. |
| **Information bias** | | |
| **Information of interest** | | **Acquired by** |
| Exposure: mode of conception | | All OD and IVF/ICSI pregnancies were included from the fertility clinic of the Erasme Hospital of the Free University of Brussels, assuring mode of conception. |
| Outcome: hypertensive disorders of pregnancy | | PIH is defined as blood pressure levels ≥140/90 mm Hg, without proteinuria, after 20 weeks of gestation. PE is defined as blood pressure levels ≥140/90 mm Hg on two measurements at least 6 h apart with proteinuria ≥0.3g/day after 20 weeks of gestation. All patients in the control group delivered at the maternity ward of the Erasme Hospital, and data were extracted from an electronic database. Some OD patients delivered in the same unit or elsewhere. When patients delivered in other units, a questionnaire was sent to their gynecologist. In some cases, the files were incomplete, so patients, their physicians, or both, were contacted to retrieve the missing information; however, the dataset remained incomplete. There is no information whether women pregnant after OD were equally treated as women pregnant with non-donor pregnancies. |
| **Selection bias** | | |
| Selection of participants | | All women with singleton pregnancies achieved after OD who gave birth after 22 weeks’ gestation between 1991 and 2013 from the fertility clinic of the Erasme Hospital of the Free University of Brussels were identified. The control group was extracted from women with singleton pregnancies achieved after IVF/ICSI who gave birth after 22 weeks’ gestation with a delivery at the Erasme Hospital during the same period. Exclusion criteria were women with Turner syndrome, cycles with cryopreserved embryos, multiple pregnancies, the application of testicular sperm extraction and cycles with preimplantation genetic diagnosis. |
| Loss of follow-up or missing data | | Due to the matching procedure, cases that were available for analysis were excluded. This percentage (48%) is likely to introduce bias. |

| **Tranquilli, *et al.* 2013** | | |
| --- | --- | --- |
| **Bias due to confounding** | | |
| **Confounding factor** | **Adjusted** | **Explanation** |
| Maternal age | No | No adjustment for confounders. |
| Multiple gestation | No |  |
| Other | No |  |
| **Information bias** | | |
| **Information of interest** | | **Acquired by** |
| Exposure: mode of conception | | All OD procedures were performed abroad because this method was not allowed in Italy. Also the non-exposed cohort could have contained women pregnant after OD that did not reveal the origin of their conception. |
| Outcome: hypertensive disorders of pregnancy | | PIH is defined as de novo hypertension appearing after 20 weeks’ gestation with a systolic blood pressure ≥140 mmHg and/or a diastolic blood pressure ≥90 mmHg. PE is defined as PIH with proteinuria ≥300 mg/day or a spot urine protein/creatinine ratio ≥30 mg/mmol. No information on the derivation of data is given. There is no information whether women pregnant after OD were equally treated as women pregnant with non-donor pregnancies. |
| **Selection bias** | | |
| Selection of participants | | Women with documented infertility who became pregnant ICSI with OD were analyzed. These pregnancies were compared to the two consecutive recorded birth obtained by autologous ICSI, and two consecutive subsequent NC deliveries in women older than 40 years. There is no information on the center of inclusion or time period. |
| Loss of follow-up or missing data | | No loss of follow-up or missing data. |

| **Van Dorp, *et al.* 2014** | | |
| --- | --- | --- |
| **Bias due to confounding** | | |
| **Confounding factor** | **Adjusted** | **Explanation** |
| Maternal age | Yes | OD and IVF pregnancies were matched by maternal age. Multivariate analysis adjusted for maternal age. |
| Multiple gestation | Yes | Subgroup analyses of singleton and multiple pregnancies. |
| Other | Yes | OD and IVF pregnancies were matched by date of embryo transfer (<3 months) and ZIP code. Multivariate analysis adjusted for donor age, socio-economic status, ethnicity and parity. |
| **Information bias** | | |
| **Information of interest** | | **Acquired by** |
| Exposure: mode of conception | | Information on fertility treatment was retrieved from medical records retrospectively. The time period and community of the control IVF group is not stated. As the origin of the control group is not known, it could have contained women pregnant after OD that did not reveal the origin of their conception. |
| Outcome: hypertensive disorders of pregnancy | | PIH is defined as at least one measurement of a diastolic blood pressure ≥90 mm Hg. PE is defined as PIH with proteinuria (>300 g of protein in the urine per day). Pregnancy outcome data was extracted from The Netherlands Perinatal Registry (PRN). This registry contains population-based information of 96% of all pregnancies in the Netherlands. The PRN is a database containing linked and validated data from three professional registries: the obstetric database for midwives, the obstetric database for gynaecologists, and the neonatal/paediatric database. There is no information whether women pregnant after OD were equally treated as women pregnant with NC pregnancies. |
| **Selection bias** | | |
| Selection of participants | | Non-anonymous OD subjects and matched IVF subjects were included. The OD cohort included all women who underwent OD treatment in the Erasmus MC Medical Centre between 1992 and 2009. Cycles without embryo transfers were excluded. |
| Loss of follow-up or missing data | | There was a small number of loss to follow up clearly depicted in a diagram, unlikely to introduce bias. |

| **Wiggins, *et al.* 2005** | | |
| --- | --- | --- |
| **Bias due to confounding** | | |
| **Confounding factor** | **Adjusted** | **Explanation** |
| Maternal age | Yes | Stratification and multivariable logistic regression analysis adjusted for maternal age. |
| Multiple gestation | Yes | Multivariable logistic regression analysis adjusted for multiple gestation. |
| Other | Yes | Multivariable logistic regression analysis adjusted for parity. |
| **Information bias** | | |
| **Information of interest** | | **Acquired by** |
| Exposure: mode of conception | | IVF services were provided from several ART centers in the San Francisco area, assuring mode of conception. |
| Outcome: hypertensive disorders of pregnancy | | PIH is defined according to the ACOG guidelines (2002) as systolic blood pressure of ≥140 mmHg or diastolic blood pressure of ≥90 mmHg that occurs after 20 weeks of gestation in a woman with previously normal blood pressure. PE is defined as PIH with ≥300mg urine protein excretion in a 24-hour period or a protein/creatinine ratio of greater than or equal to 0.3 or urine protein dipstick 1+. Office and hospital records of all deliveries of all patients were reviewed. Patients in both groups were given the same standard high-risk obstetric care under the care of the same group of obstetricians. Perinatology consultants were involved whenever there were additional high-risk factors such as hypertension, diabetes, or preterm labor. |
| **Selection bias** | | |
| Selection of participants | | All OD pregnancies between 1990 and May 2004 in different fertility centres in San Francisco were identified. The control group were autologous IVF pregnancies that conceived at the same centres during the same period. Once their obstetric care was established, all patients were under the care of board certified obstetrician/gynecologists in 1 of 3 related private practice medical groups at California Pacific Medical Center, San Francisco, California. |
| Loss of follow-up or missing data | | No loss of follow-up or missing data. |

| **Wolff, *et al.* 1997** | | |
| --- | --- | --- |
| **Bias due to confounding** | | |
| **Confounding factor** | **Adjusted** | **Explanation** |
| Maternal age | Yes | Multivariable logistic regression analysis adjusted for maternal age. |
| Multiple gestation | Yes | Multivariable logistic regression analysis adjusted for fetal number, and subgroup analysis on singleton pregnancies. |
| Other | Yes | Multivariable logistic regression analysis adjusted for gravidity, parity, previous abortions, ethnicity, intoxications and relevant medical history. |
| **Information bias** | | |
| **Information of interest** | | **Acquired by** |
| Exposure: mode of conception | | Data on OD treatment was retrieved from the two participating hospitals, assuring mode of conception. However, the NC cohort could have contained women pregnant after OD that did not reveal the origin of their conception. |
| Outcome: hypertensive disorders of pregnancy | | No definition on PIH and PE is given. Study data was collected using a questionnaire. In 50% of the respondents, specific questions required verification by telephone interviews with the patient or her obstetrician. The prenatal care and deliveries were managed by various obstetricians, both in academic and private practice settings. |
| **Selection bias** | | |
| Selection of participants | | All OD pregnancies that conceived between 1992 and 1995 at either the University of North Carolina Hospitals or Duke University Medical Centre were identified. The control group were spontaneous pregnancies in women >38 yrs obtained from a perinatal database maintained by the University of North Carolina Hospital between January 1988 and April 1996. |
| Loss of follow-up or missing data | | No loss of follow-up or missing data. |

| **Woo, *et al.* 2017** | | |
| --- | --- | --- |
| **Bias due to confounding** | | |
| **Confounding factor** | **Adjusted** | **Explanation** |
| Maternal age | Yes | Logistic regression analysis with an exchangeable covariance structure to account for birth outcomes to the same woman and differences in age. |
| Multiple gestation | Yes | Only singleton pregnancies included. |
| Other | Yes | The use of gestational surrogates as their own control group was done to keep confounders specific to the carrier constant. |
| **Information bias** | | |
| **Information of interest** | | **Acquired by** |
| Exposure: mode of conception | | Data on ART treatment was retrieved from the two participating hospitals, assuring mode of conception. However, |
| Outcome: hypertensive disorders of pregnancy | | Clinical diagnosis of PIH and PE was based on the discretion of the primary obstetrical provider. Because there was a wide range of providers, specific definitions used to establish a diagnosis of obstetrical complication was not obtained and we assumed that standard of care was practiced. Data on perinatal outcomes were collected both by means of the electronic survey instrument and through a detailed review of medical records. Medical records were obtained from the gestational surrogacy agencies and from USC Fertility. There is no information whether women pregnant after gestational surrogacy were equally treated as in their previous NC pregnancies. |
| **Selection bias** | | |
| Selection of participants | | Gestational surrogates who achieved clinical pregnancies from commissioned embryos from January 1995 to December 2010 were identified at two large California-based surrogacy agencies. Gestational surrogates who achieved a clinical pregnancy from January 1990 to December 2014 at the University of Southern California Fertility Center were also identified. All antecedent pregnancies that were spontaneously achieved by these women were included as control. |
| Loss of follow-up or missing data | | Demographic information of 25% of the surrogates was missing. |

| **Yadav, *et al.* 2018** | | |
| --- | --- | --- |
| **Bias due to confounding** | | |
| **Confounding factor** | **Adjusted** | **Explanation** |
| Maternal age | Yes | Multivariable logistic regression analysis adjusted for maternal age. |
| Multiple gestation | Yes | Subgroup analyses for singleton and multiple pregnancies. |
| Other | No |  |
| **Information bias** | | |
| **Information of interest** | | **Acquired by** |
| Exposure: mode of conception | | ART treatment data was retrieved from the ART institute of the participating center, assuring mode of conception. |
| Outcome: hypertensive disorders of pregnancy | | PE is defined as blood pressure ≥140/90 mmHg with proteinuria after 20-week gestation. There is no information on how data was retrieved. During pregnancy, both groups were followed up in antenatal clinic of the institute. However, there is no information whether women pregnant after OD were equally treated as women pregnant with non-donor pregnancies. |
| **Selection bias** | | |
| Selection of participants | | All women between the age groups of 20 and 45 years who conceived from OD between December 12, 2010, and December 31, 2016, and compared with all women who underwent autologous IVF in during the same period at the center for ART of the institute. Women who underwent OD using siblings as donors prior to this period were all excluded. |
| Loss of follow-up or missing data | | No loss of follow-up or missing data. |

| **Yadav, *et al.* 2022** | | |
| --- | --- | --- |
| **Bias due to confounding** | | |
| **Confounding factor** | **Adjusted** | **Explanation** |
| Maternal age | Yes | Multivariable logistic regression analysis adjusted for maternal age. |
| Multiple gestation | No |  |
| Other | No |  |
| **Information bias** | | |
| **Information of interest** | | **Acquired by** |
| Exposure: mode of conception | | No information is given on the retrieval of OD treatment data. The NC cohort could have contained women pregnant after OD that did not reveal the origin of their conception. |
| Outcome: hypertensive disorders of pregnancy | | No definition is given for PIH and PE. Obstetric and perinatal profiles were taken from the hospital database. There is no information whether women pregnant after OD were equally treated as women pregnant with NC pregnancies. |
| **Selection bias** | | |
| Selection of participants | | All women between the ages of 20-45 years who conceived from OD between December 1 2011 to September 30 2017. As control groups, all women who had spontaneous conception were selected in the same time period in a ratio of 1:3. There is no information about the center of inclusion. Women who underwent OD using siblings as donors prior to this period were all excluded. |
| Loss of follow-up or missing data | | No loss of follow-up or missing data. |

| **Yamada, *et al.* 2015 (conference abstract)** | | |
| --- | --- | --- |
| **Bias due to confounding** | | |
| **Confounding factor** | **Adjusted** | **Explanation** |
| Maternal age | No | No information on adjustment for confounders in conference abstract. |
| Multiple gestation | No |  |
| Other | No |  |
| **Information bias** | | |
| **Information of interest** | | **Acquired by** |
| Exposure: mode of conception | | ART treatment is conducted in St. Luke's International Hospital, Tokyo. However, the NC cohort could have contained women pregnant after OD that did not reveal the origin of their conception. |
| Outcome: hypertensive disorders of pregnancy | | No definition on PIH is stated in the conference abstract. Data of delivery were reviewed from the participating hospital. There is no information whether women pregnant after OD were equally treated as women pregnant with non-donor pregnancies. |
| **Selection bias** | | |
| Selection of participants | | Spontaneous pregnancies were compared to those with infertility treatments (such as clomiphene, gonadotropin, IUI, IVF, ICSI and OD) from St. Luke's International Hospital, Tokyo, Japan in 2014. Any cases of stillbirth, multiple pregnancy and uncertainty of their treatments were excluded. |
| Loss of follow-up or missing data | | No loss of follow-up or missing data. |

| **Zeman, *et al.* 2022** | | |
| --- | --- | --- |
| **Bias due to confounding** | | |
| **Confounding factor** | **Adjusted** | **Explanation** |
| Maternal age | No | No information on adjustment for confounders in conference abstract. |
| Multiple gestation | No |  |
| Other | No |  |
| **Information bias** | | |
| **Information of interest** | | **Acquired by** |
| Exposure: mode of conception | | ART treatment data was retrieved from the participating center, assuring mode of conception. |
| Outcome: hypertensive disorders of pregnancy | | No definition on PE is stated in the conference abstract. There is no information on how outcome data was retrieved. There is no information whether women pregnant after OD were equally treated as women pregnant with non-donor pregnancies. |
| **Selection bias** | | |
| Selection of participants | | This study included pregnant patients who performed combined first trimester screening for PE, FGR and aneuploidies between 2019 and 2021. These patients were divided in NC, IVF, and OD pregnancies. Multiple pregnancies, high risk for aneuploidies, and pregnancies after ART in another IVF center were excluded. |
| Loss of follow-up or missing data | | No loss of follow-up or missing data. |

# Supplementary Data File S3. Sensitivity and meta-regression analyses

## A. Sensitivity analyses – Addition of aggregated data

*
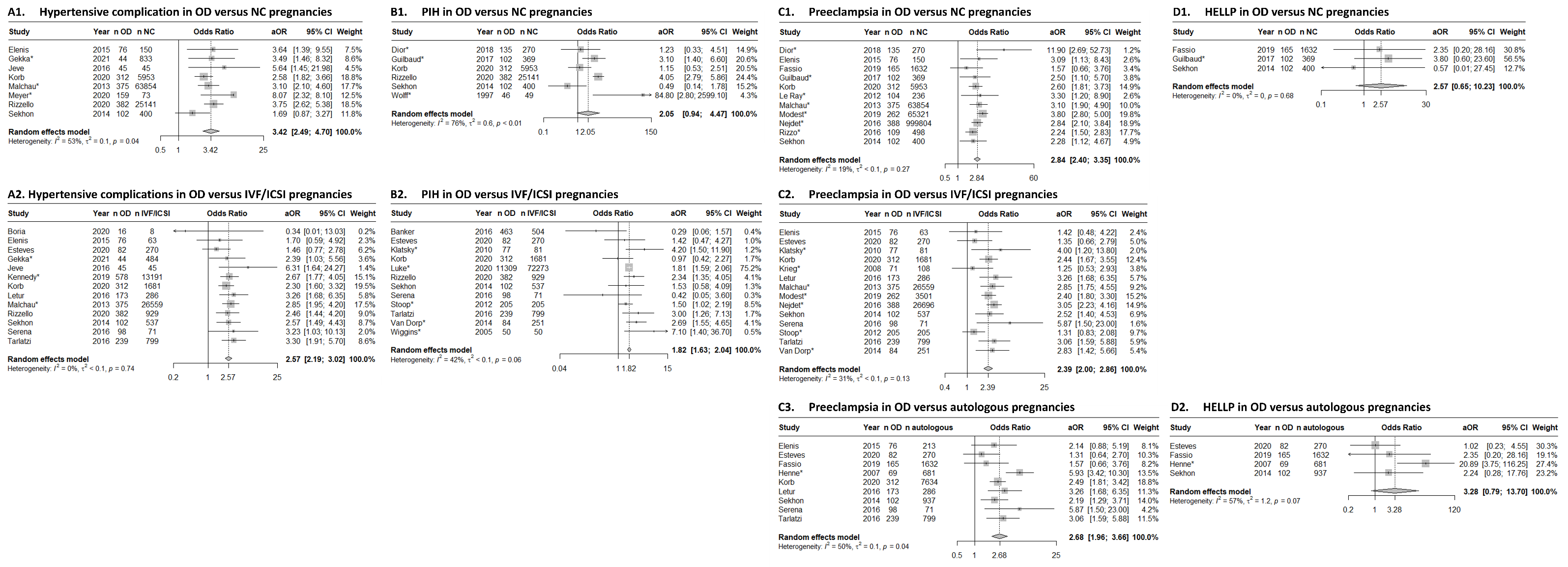
*

* Studies of which the aggregate data were used, as they did not provide individual participant data. The odds ratios at least adjusted for maternal age were extracted.


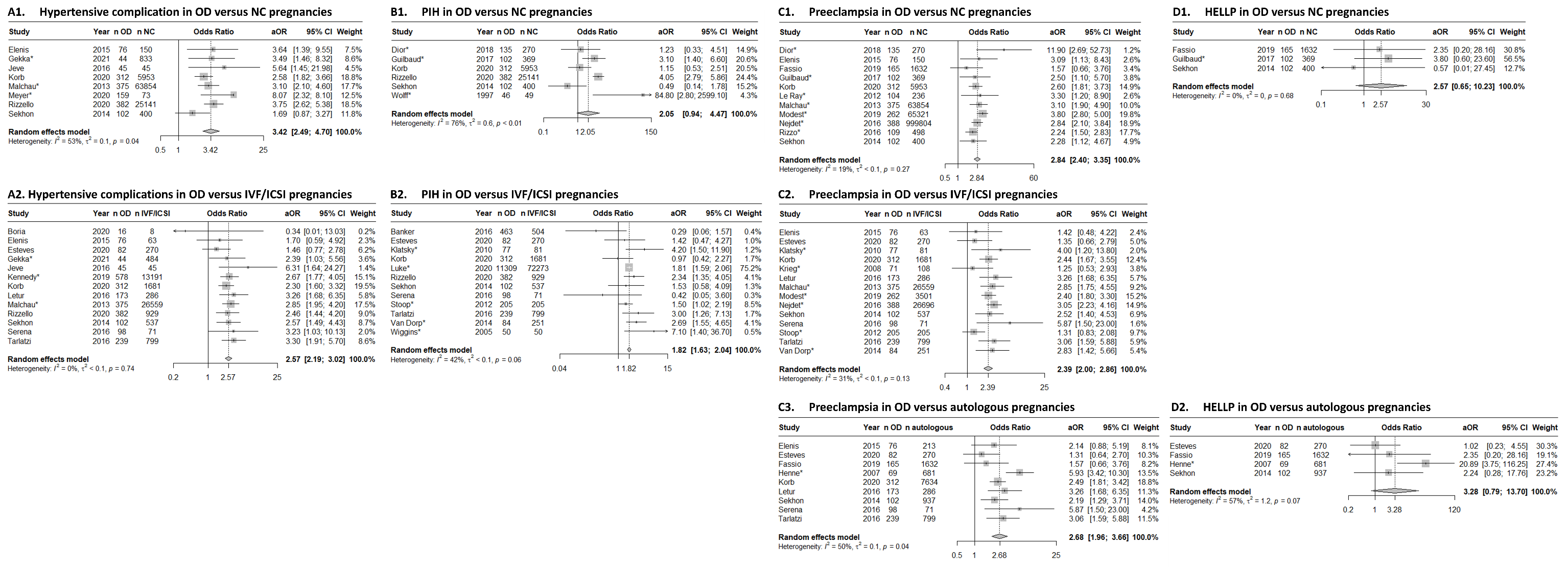


* Studies of which the aggregate data were used, as they did not provide individual participant data. The odds ratios at least adjusted for maternal age were extracted.

## B. Sensitivity analyses – Methodological quality

Only using studies with low to moderate risk of bias using the ROBINS-I tool.


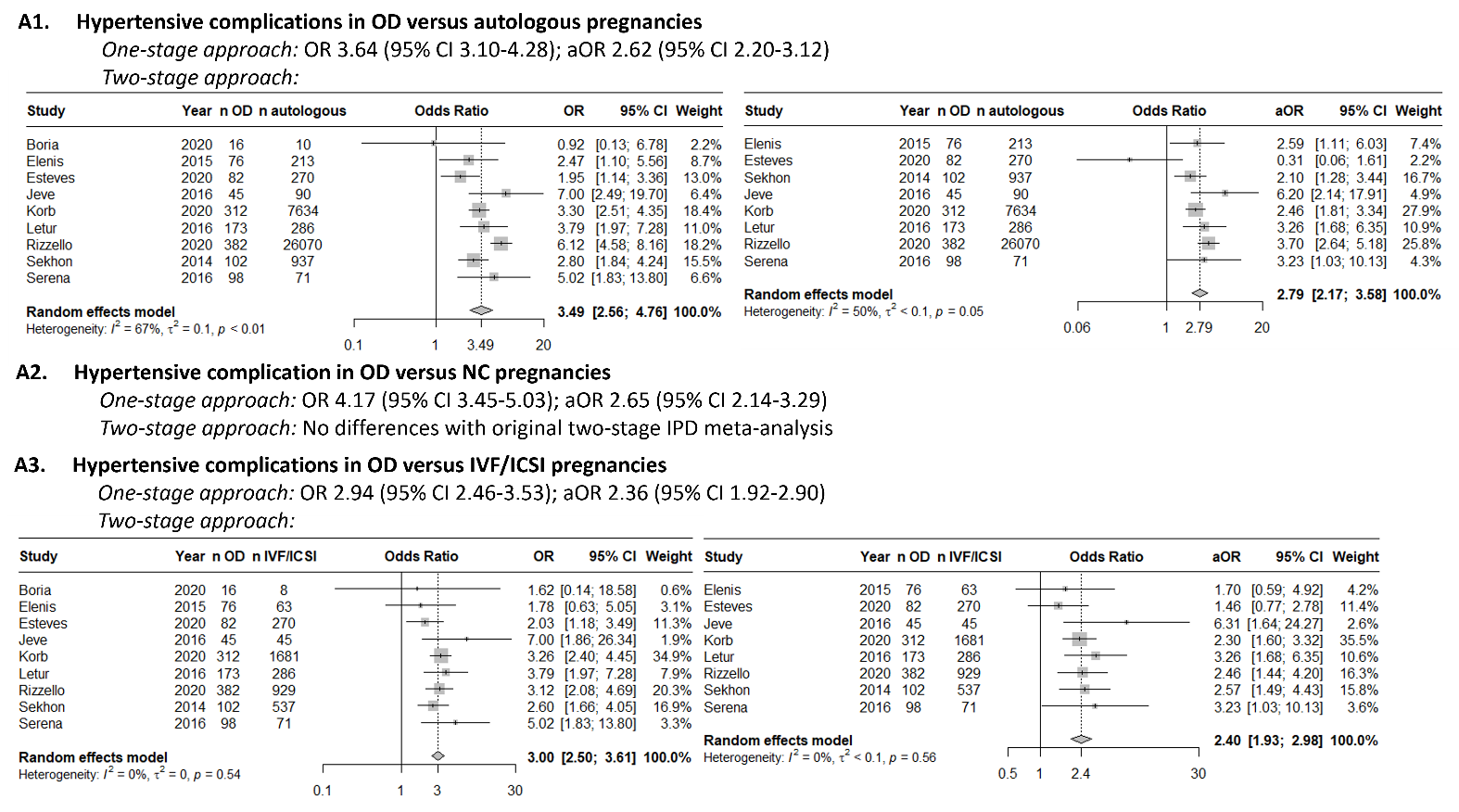


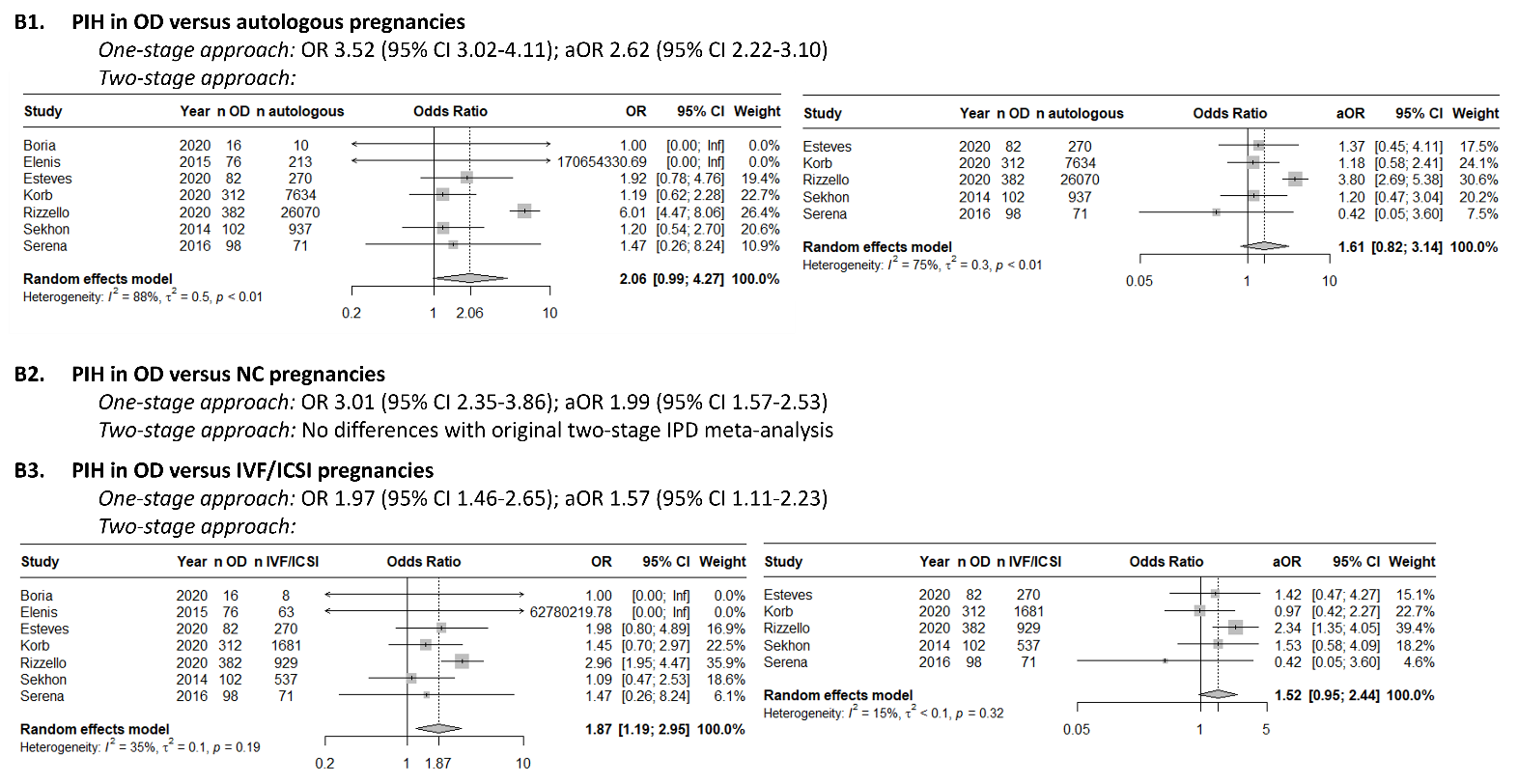


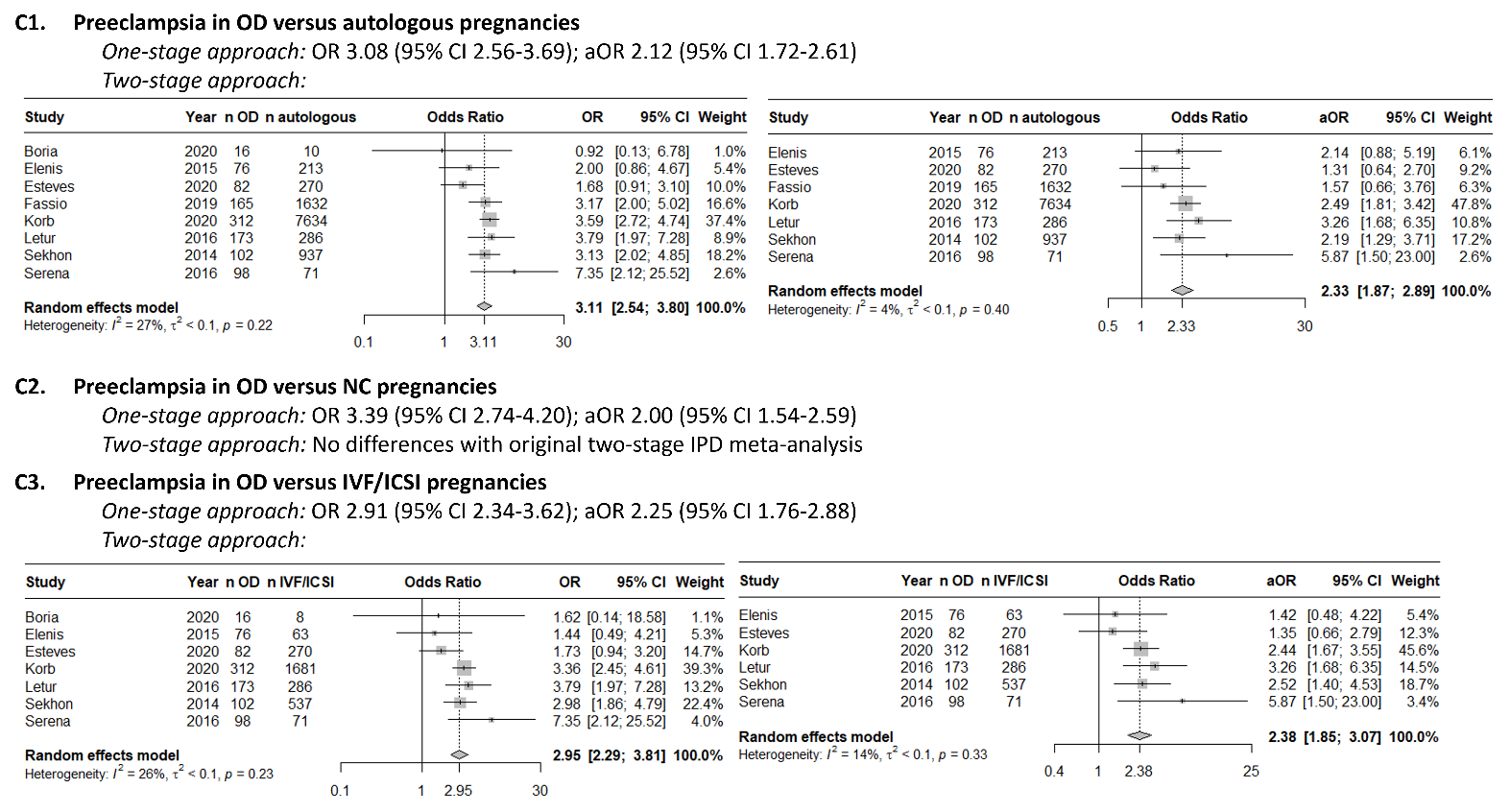


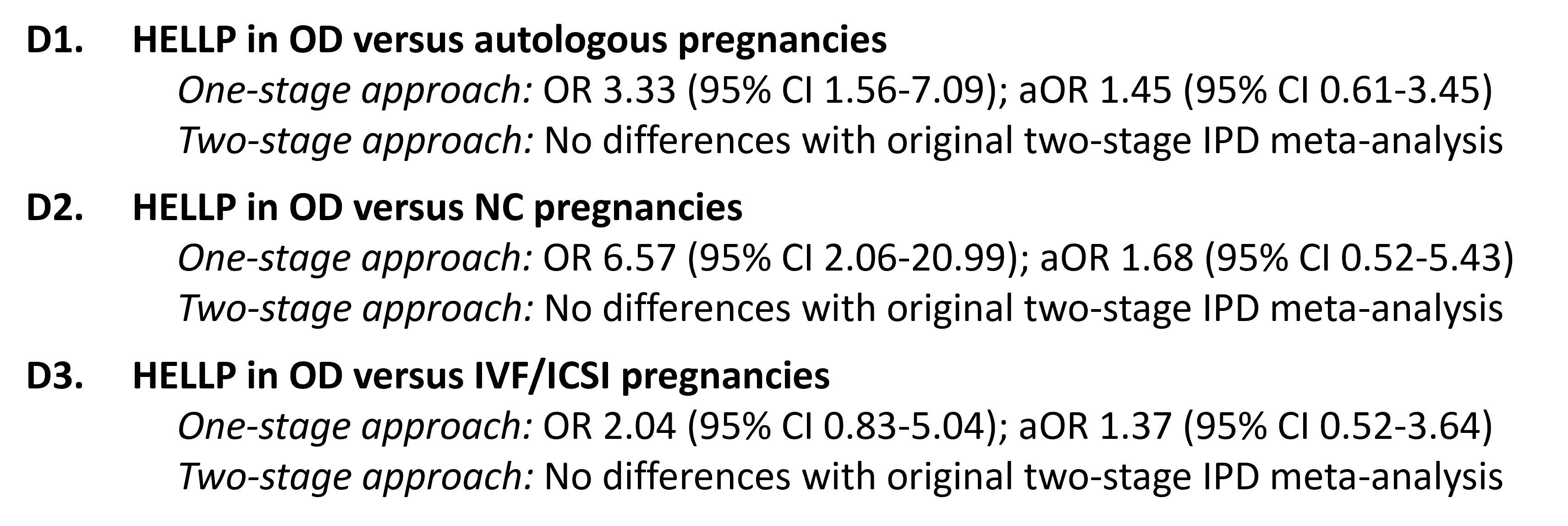


## C. Meta-regression analyses - Year of publication

### Hypertensive complications in total

*Hypertensive complications in OD versus autologous pregnancy*


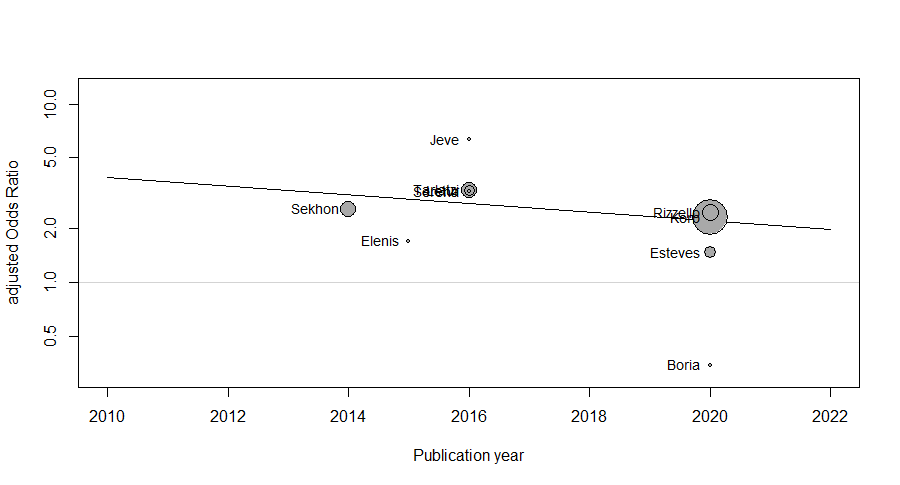


|  | OR | 95% CI |
| --- | --- | --- |
| Effect of year | 0.95 | 0.87 - 1.03 |

*Hypertensive complications in OD versus NC pregnancy*


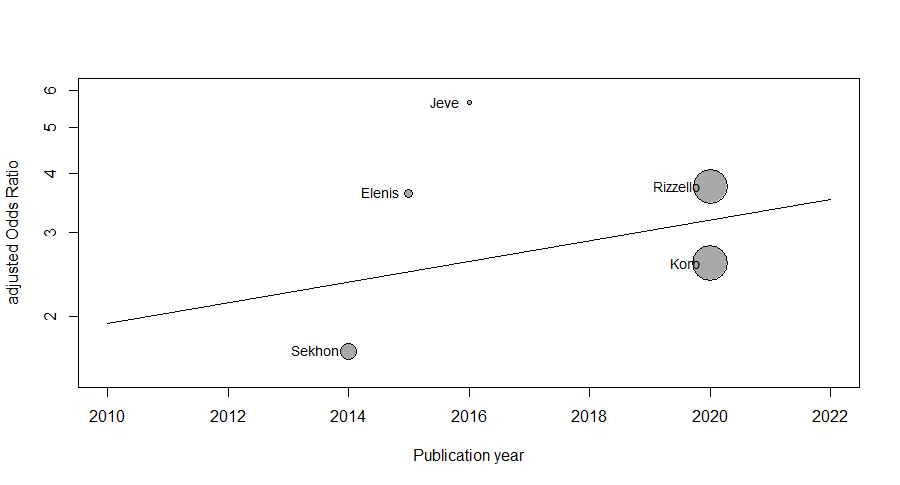


|  | OR | 95% CI |
| --- | --- | --- |
| Effect of year | 1.05 | 0.92 - 1.20 |

*Hypertensive complications in OD versus IVF/ICSI pregnancy*


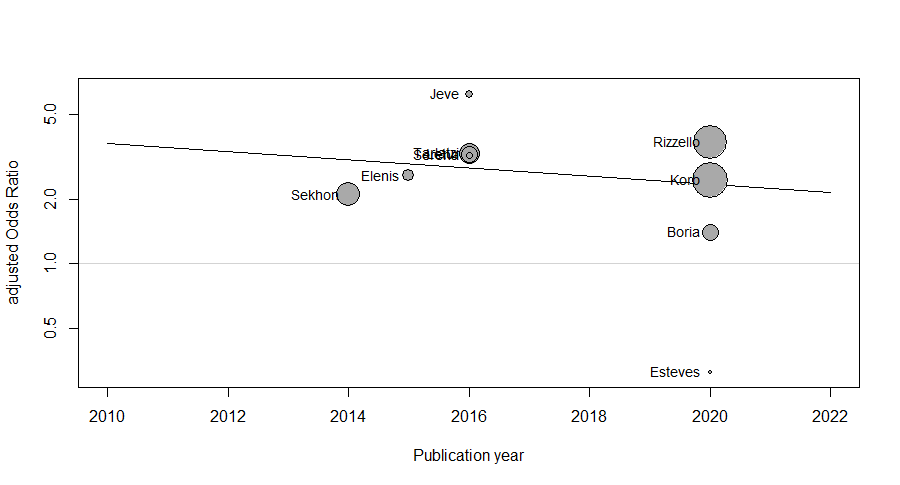


|  | OR | 95% CI |
| --- | --- | --- |
| Effect of year | 0.96 | 0.85 - 1.08 |

### Pregnancy-induced hypertension

*PIH in OD versus autologous pregnancy*


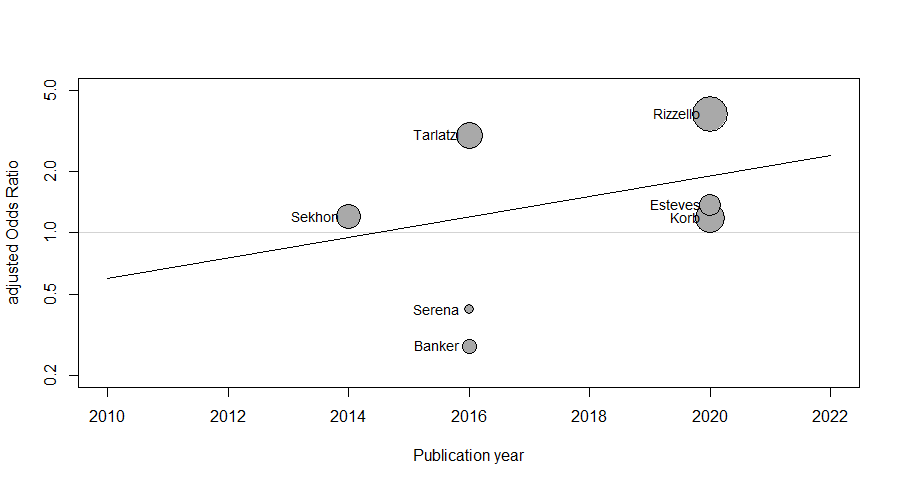


|  | OR | 95% CI |
| --- | --- | --- |
| Effect of year | 1.12 | 0.85 - 1.47 |

*PIH in OD versus NC pregnancy*


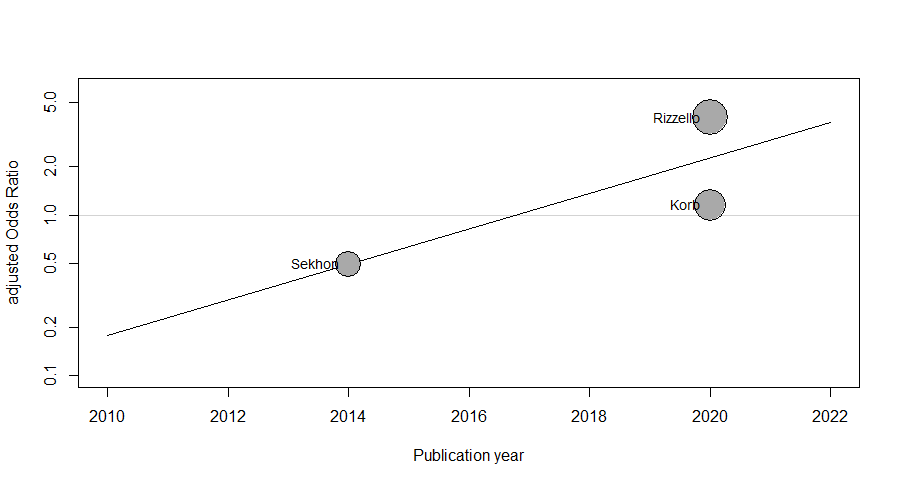


|  | OR | 95% CI |
| --- | --- | --- |
| Effect of year | 1.29 | 0.86 - 1.93 |

*PIH in OD versus IVF/ICSI pregnancy*


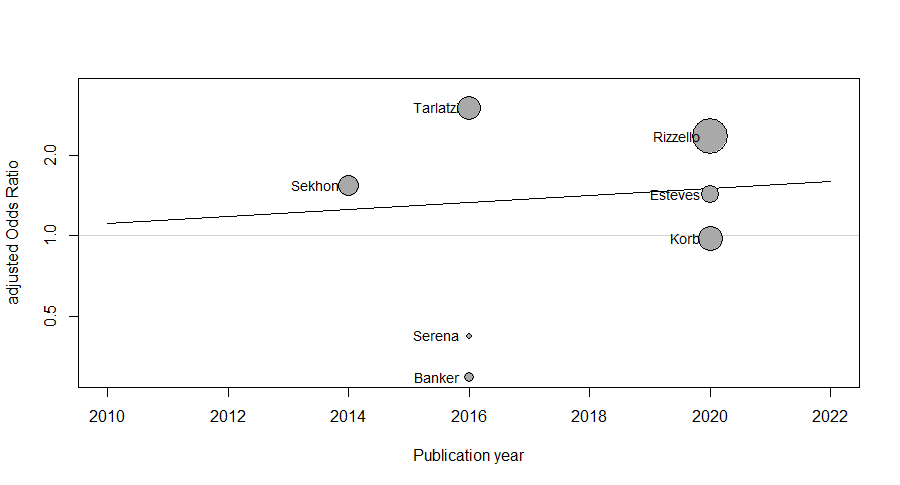


|  | OR | 95% CI |
| --- | --- | --- |
| Effect of year | 1.03 | 0.82 - 1.30 |

### Preeclampsia

*Preeclampsia in OD versus autologous pregnancies*


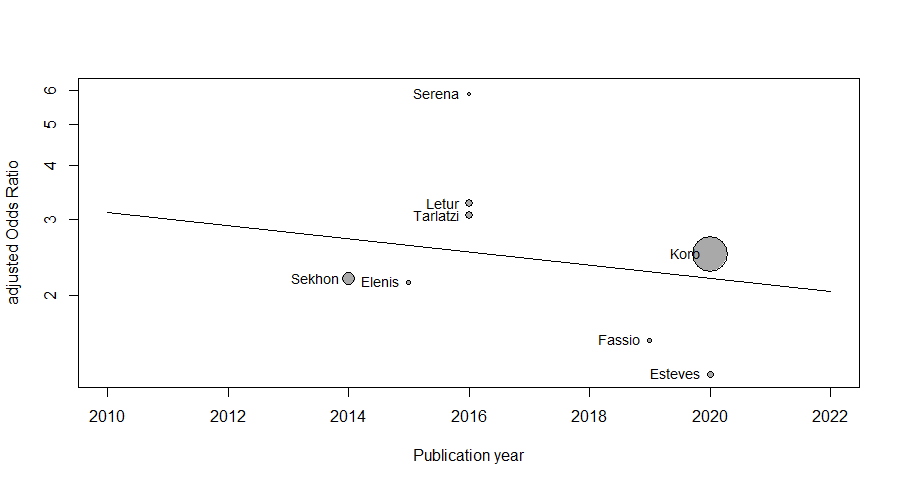


|  | OR | 95% CI |
| --- | --- | --- |
| Effect of year | 0.97 | 0.88 – 1.06 |

*Preeclampsia in OD versus NC pregnancies*


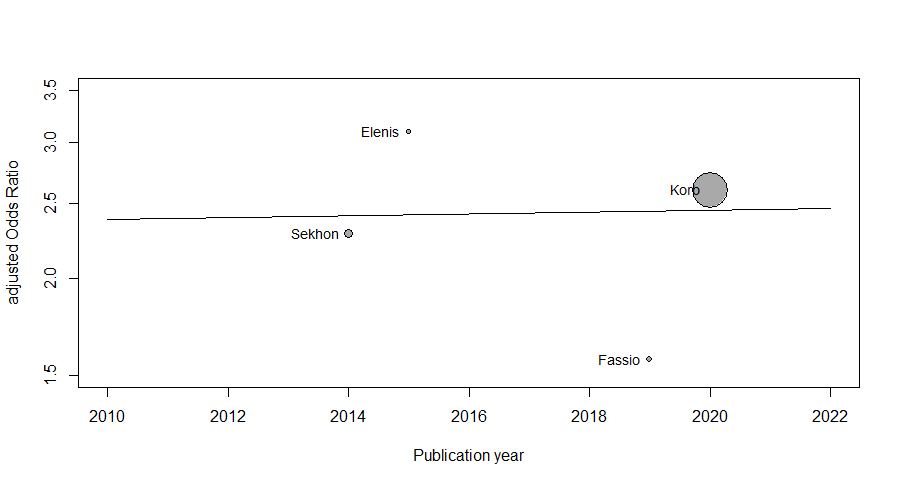


|  | OR | 95% CI |
| --- | --- | --- |
| Effect of year | 1.00 | 0.89 - 1.13 |

*Preeclampsia in OD versus IVF/ICSI pregnancies*


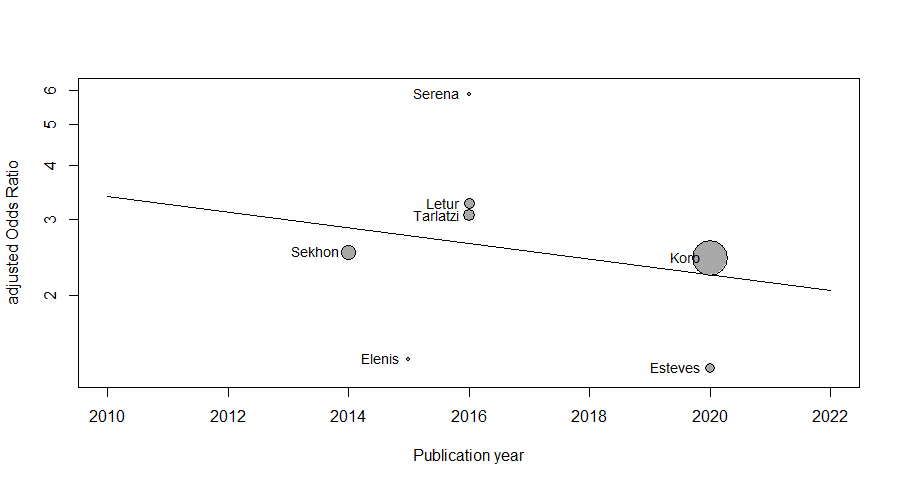


|  | OR | 95% CI |
| --- | --- | --- |
| Effect of year | 0.96 | 0.87 - 1.06 |

### HELLP

*HELLP in OD versus autologous pregnancy*


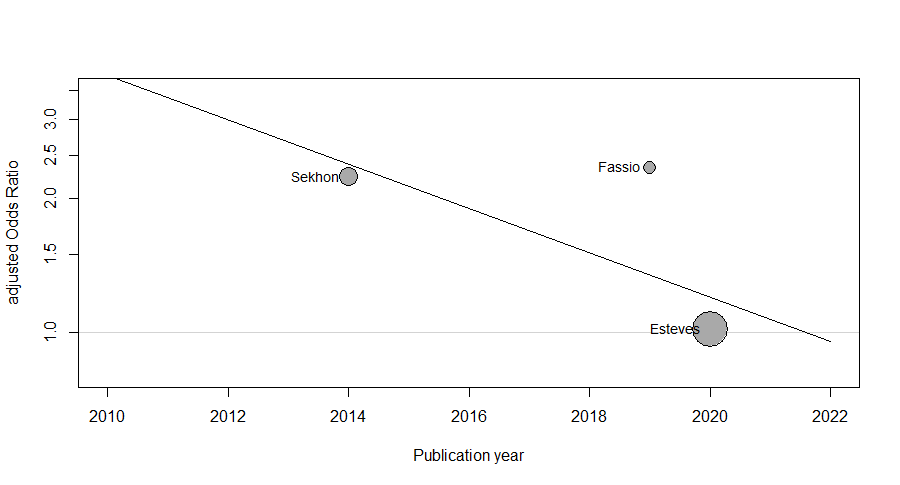


|  | OR | 95% CI |
| --- | --- | --- |
| Effect of year | 0.89 | 0.59 - 1.36 |

*Due to IPD of only two studies, the meta-regression for HELLP in OD versus NC and IVF/ICSI pregnancy is not possible.*

# Supplementary Figure S1. Publication bias

|  | **OD versus NC** | | | | **OD versus IVF/ICSI** | |
| --- | --- | --- | --- | --- | --- | --- |
|  | *IPD* | | *Aggregate data* | | *IPD* | *Aggregate data* |
| **PIH** | 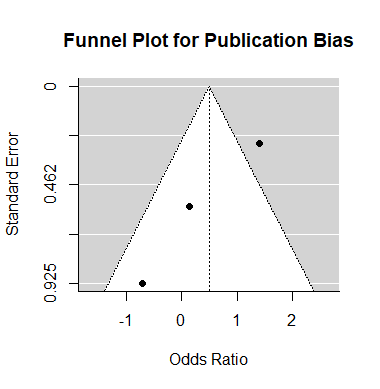  p = 0.01 | | 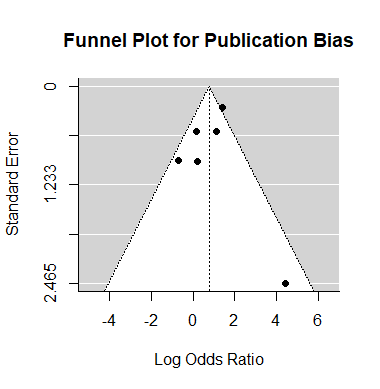  p = 0.79 | | 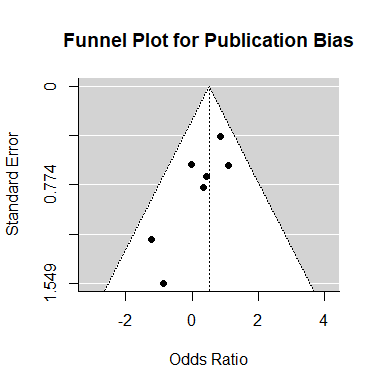  p = 0.08 | 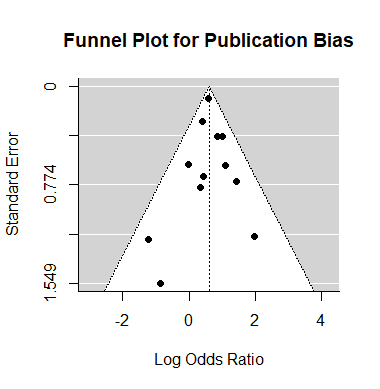  p = 0.95 |
| **PE** | 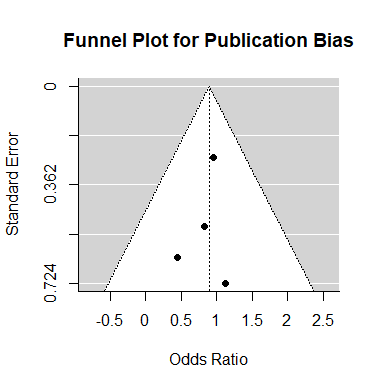  p = 0.74 | | 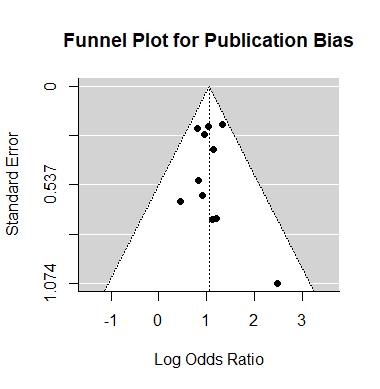  p = 0.87 | | 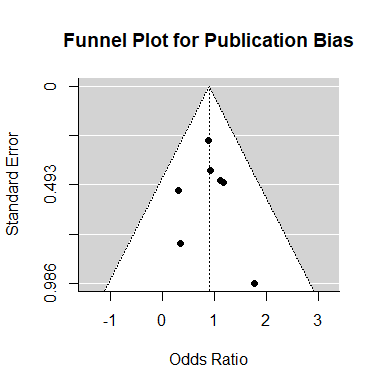  p = 0.92 | 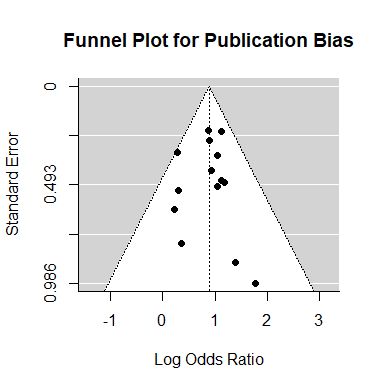  p = 0.83 |
| **Hypertensive complications** | 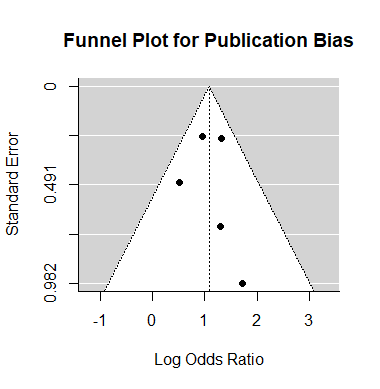  p = 0.85 | | 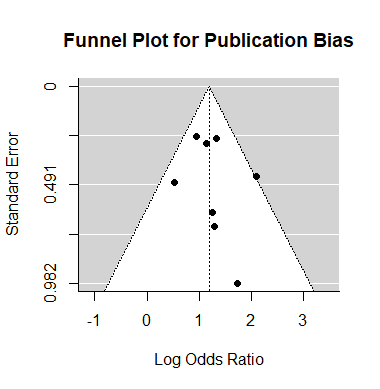  p = 0.50 | | 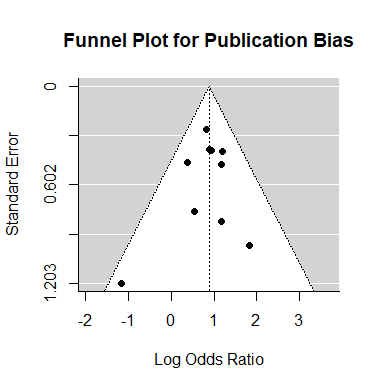  p = 0.66 | 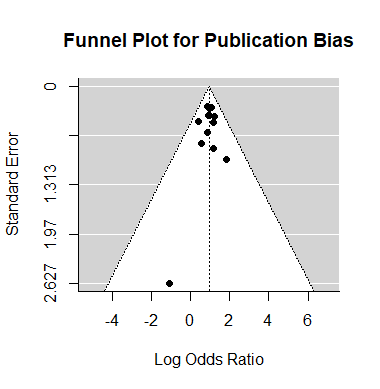  p = 0.82 |
|  |  | |  | |  |  |
|  | **OD versus autologous** | | |  |  |  |
|  | *IPD* | *Aggregate data* | |  |  |  |
| **PIH** | 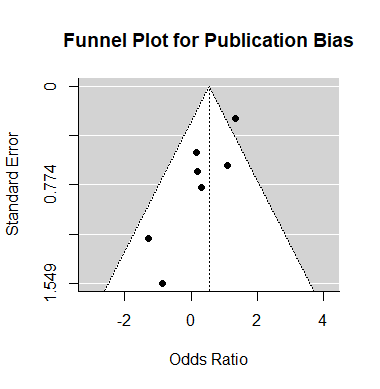  p = 0.003 | NA | |  |  |  |
| **PE** | 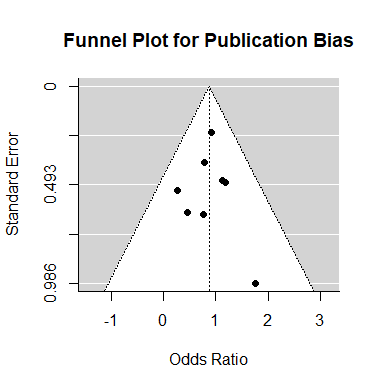  p = 0.98 | 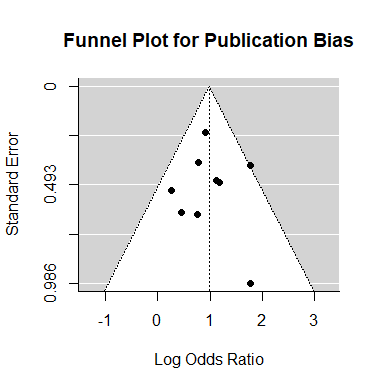  p = 0.99 | |  |  |  |
| **Hypertensive complications** | 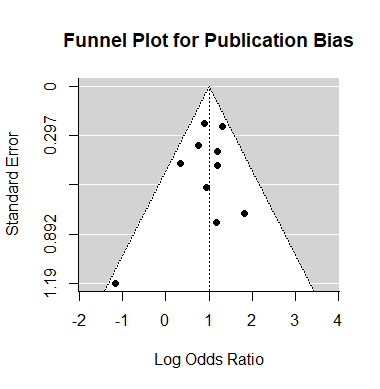  p = 0.43 | NA | |  |  |  |

OD = oocyte donation; NC = naturally conceived; IVF/ICSI = *in vitro* fertilization/intracytoplasmic sperm injection; PIH = pregnancy-induced hypertension; PE = preeclampsia; p = p-value calculated with the Egger’s test; NA = aggregate data not available
